# Antibacterial Activity of Endophytic Actinomycetes Isolated from the Medicinal Plant *Vochysia divergens* (Pantanal, Brazil)

**DOI:** 10.3389/fmicb.2017.01642

**Published:** 2017-09-06

**Authors:** Francielly M. W. R. Gos, Daiani C. Savi, Khaled A. Shaaban, Jon S. Thorson, Rodrigo Aluizio, Yvelise M. Possiede, Jürgen Rohr, Chirlei Glienke

**Affiliations:** ^1^Department of Basic Pathology, Federal University of Paraná Curitiba, Brazil; ^2^Department of Genetics, Federal University of Paraná Curitiba, Brazil; ^3^Department of Pharmaceutical Sciences, College of Pharmacy, University of Kentucky Lexington, KY, United States; ^4^Center for Pharmaceutical Research and Innovation, College of Pharmacy, University of Kentucky Lexington, KY, United States; ^5^Department of Biology, Federal University of Mato Grosso do Sul Campo Grande, Brazil

**Keywords:** actinomycetes, endophytes, *Vochysia divergens*, pantanal, MRSA, secondary metabolites

## Abstract

Endophytic actinomycetes from medicinal plants produce a wide diversity of secondary metabolites (SM). However, to date, the knowledge about endophytes from Brazil remains scarce. Thus, we analyzed the antimicrobial potential of 10 actinomycetes isolated from the medicinal plant *Vochysia divergens* located in the Pantanal sul-mato-grossense, an unexplored wetland in Brazil. Strains were classified as belonging to the *Aeromicrobium, Actinomadura, Microbacterium, Microbispora, Micrococcus, Sphaerisporangium, Streptomyces*, and *Williamsia* genera, through morphological and 16S rRNA phylogenetic analyzes. A susceptibility analysis demonstrated that the strains were largely resistant to the antibiotics oxacillin and nalidixic acid. Additionally, different culture media (SG and R5A), and temperatures (28 and 36°C) were evaluated to select the best culture conditions to produce the active SM. All conditions were analyzed for active metabolites, and the best antibacterial activity was observed from metabolites produced with SG medium at 36°C. The LGMB491 (close related to *Aeromicrobium ponti*) extract showed the highest activity against methicillin-resistant *Staphylococcus aureus* (MRSA), with a MIC of 0.04 mg/mL, and it was selected for SM identification. Strain LGMB491 produced 1-acetyl-β-carboline (**1**), indole-3-carbaldehyde (**2**), 3-(hydroxyacetyl)-indole (**4**), brevianamide F (**5**), and cyclo-(L-Pro-L-Phe) (**6**) as major compounds with antibacterial activity. In this study, we add to the knowledge about the endophytic community from the medicinal plant *V. divergens* and report the isolation of rare actinomycetes that produce highly active metabolites.

## Introduction

Endophytes are microorganisms that inhabit the internal tissues of plants without causing any negative effects, and actinomycetes isolated from plants have been widely studied due their ability to produce active metabolites (Kim et al., 2000; Zhao et al., 2011; Kadiri et al., 2014; Golinska et al., 2015; Savi et al., 2015a,b). Actinomycetes have been used for drug discovery for more than five decades, producing more than 10,000 bioactive compounds. Of these ~75% are produced by *Streptomyces*, the by far mostly explored actinomycete genus. The remaining 25% bioactive compounds were isolated from “rare actinomycetes”, i.e., actinomycetes isolated in lower frequency than *Streptomyces* (Rong and Huang, 2012; Tiwari and Gupta, 2012). Since, the rare actinomycetes are an underexplored group, the use of these organisms, and their compounds have gained great importance in drug discovery programs (Rong and Huang, 2012; Tiwari and Gupta, 2012), mainly to combat infections caused by resistant microorganisms. The widespread use of broad-spectrum antibiotics has created a strong selective pressure, resulting in survival, and spread of resistant bacteria (Davies and Davies, 2010). The increase in bacterial resistance is a major concern for public health (Ventola, 2015). Unfortunately, many pharmaceutical companies have reduced or eliminated their search for new antibiotics, due to economic reasons, exasperating the problem further (Borrero et al., 2014). In order to find microorganisms with potential to produce active metabolites our group has been searching endophytic microorganisms from medicinal plants located in underexplored environments, such as the Brazilian wetland regions (Savi et al., 2015a,b; Hokama et al., 2016; Peña et al., 2016; Santos et al., 2016; Tonial et al., 2017). The Brazilian Pantanal is the largest wetland in the world, and it is characterized by two seasons: flooding and the dry. Hence, the Pantanal has developed a peculiar biological diversity regarding its fauna and flora (Alho, 2008). According to Arieira and Cunha (2006), only 5% of the species of plants of the Pantanal can survive the stress caused by drought and flood periods. Among them is the medicinal plant *Vochysia divergens*, which is commonly used in form of syrups and teas for the treatment of colds, coughs, fever, pneumonia, and other diseases (Pott et al., 2004). In a study carried out with endophytes from *V. divergens*, Savi et al. (2015a) identified actinomycetes able to produce highly active metabolites. However, the study was performed with a small number of isolates, and the diversity of *V. divergens* remained little explored. Thus, the focus of this study is to identify endophytic actinomycetes from the medicinal plant *V. divergens* and to assay their secondary metabolites, dependent on different culture conditions, against clinical pathogens associated with antibiotic resistance.

## Materials and methods

### Sample collection

*V. divergens* leaves with no marks or injuries were collected from 21 plants located in the Pantanal sul-mato-grossense/Brazil, specifically in two regions of the Pantanal of Miranda, Abobral (19°30′09.5″S, 57°02′32.2″W) and São Bento (19°28′53.9″S, 57°02′36.9″W).

### Isolation of actinomycetes

The leaves from *V. divergens* were subjected to surface sterilization according to a protocol described by Petrini (1986). The leaves were fragmented (8 × 8 mm) and deposited on petri dishes containing starch casein agar (SCA) (Mohseni et al., 2013), with nalidixic acid (50 μg/mL) and cycloheximide (50 μg/mL). The plates were incubated at 28°C for 30 days, and were examined daily for the presence of colonies. The actinomycetes isolates were deposited in the Laboratório de Genética de Microrganismos (LabGeM) culture collection, Federal University of Paraná, Brazil (http://www.labgem.ufpr.br/).

### Identification

#### Morphological analysis

Four different culture media were used to access the macro-morphological characteristics, ISP2—Agar yeast-malt extract; ISP3—Oat Agar; ISP4—Agar Starch and inorganic salts; ISP5—Glycerol Asparagine Agar (Shirling and Gottlieb, 1966). The isolates were streaked on the plates and incubated at 28°C for 21 days. The characteristics evaluated were growth rate, the formation and color of aerial spore mass and substrate mycelia.

#### Molecular taxonomy

Total genomic DNA was extracted from 3 day old cultures using the method described by Raeder and Broda (1985). Partial sequence of the 16S rRNA gene was amplified using primers 9F (5′GAGTTTGATCCTGGCTCAG3′) and 1541R (5′AAGGAGGTGATCCAGCC3′), as described by Savi et al. (2016). The PCR product was purified using Exo1 and FastAP enzymes (GE Healthcare, USA), and sequenced using the BigDye® Terminator v3.1 Kit. The products were purified with SephadexG50 and submitted to an ABI3500® automated sequencer (Applied Biosystems, Foster City, CA, USA). Consensus sequences were analyzed and aligned using Mega 6.0 (Tamura et al., 2013) and BioEdit, and compared to sequences available in the GenBank database (http://www.ncbi.nlm.nih.gov/BLAST/). Type strain sequences were found through search in the List of Prokaryotic Names with Standing Nomenclature database (http://www.bacterio.net/). All sequences obtained were deposited in the GenBank, the accession numbers are listed in Table 1. For Bayesian inference analysis, a Markov Chain Monte Carlo (MCMC) algorithm was used to generate phylogenetic trees with posterior probabilities using MrBayesv3.2.6 x86 (Ronquist et al., 2011). GRT evolutionary model was determined using the Akaike Information Criterion (AIC) in R software (R Core Team, 2017) and the phangorn package (Schliep, 2011). Comparisons of sequences with respect to their percentile similarity were estimated using the R software (R Core Team, 2017) and the pegas package (Paradis, 2010).

**Table 1:** Identification, place, and source of isolation and morphological characteristic of endophytic actinomycetes isolates, morphological characteristics 21 days after inoculation in four different culture media at 28°C.

**Strain genera**	**NCBI genbank accession n°**	**Place/Source of isolation**	**ISP2—Agar yeast-malt extract**			**ISP3—Oat agar**			**ISP4—Agar starch and inorganic salts**			**ISP5—Glycerol asparagine agar**		
			**Aerial spore mass**	**Substrate mycelium**	**Grown**	**Aerial spore mass**	**Substrate mycelium**	**Grown**	**Aerial spore mass**	**Substrate mycelium**	**Grown**	**Aerial sporemass**	**Substrate mycelium**	**Grown**
*Actinomadura* sp. LGMB466	KY458125	Abobral Leaf	Moderated: White	Brown	+++	Abundant: White	Yellow	+++	Low: White	Yellow	+	Low: White	Pink	++
*Actinomadura* sp. LGMB487	KY421547	Abobral Leaf	Moderated: White	Ivory-white	+++	Abundant: White	Yellow	+++	Low: White	Yellow	+	Low: White	Ivory-white	++
*Aeromicrobium ponti* LGMB491	KY411896	Abobral Leaf	None	Yellow	++	None	Yellow	++	None	Yellow	+++	None	Yellow	+++
*Microbacterium* sp. LGMB471	KY423334	São Bento Leaf	None	Yellow	+++	None	Yellow	+++	None	Ivory-white	++	None	Ivory-white	+++
*Microbispora* sp. LGMB461	KY411900	São Bento Stem	Abundant: White	Ivory-white	+++	Abundant: White	Ivory-white	+++	Abundant: White	Ivory-white	+	Abundant: White	White	+++
*Microbispora* sp. LGMB465	KY411898	São Bento Stem	Moderated: White	Ivory-white	+++	Abundant: White	Ivory-white	+++	Abundant: White	Ivory-white	+	Abundant: White	Ivory-white	++
*Micrococcus* sp. LGMB485	KY423496	Abobral Leaf	None	White	+++	None	White	+++	None	White	+++	None	White	+++
*Sphaerisporangium* sp. LGMB482	KY458126	Abobral Stem	Abundant: White	Brown	+++	Abundant: White	Pink	+++	Abundant: Pink	Red/Ivory-white	+++	Abundant: White	Ivory-white	++
*S. thermocarboxydus* LGMB483	KY423333	Abobral Stem	Abundant: Gray	Gray	+++	Moderated: White	Ivory-white	+++	Abundant: White	Gray	+++	Abundant: White	Brown	+++
*Williamsia serinedens* LGMB479	KY421546	Abobral Stem	None	Orange	+++	None	Light orange	+++	None	Orange	+++	None	Orange	+++

*+++, Abundant; ++, Moderated; +, low*.

#### Antibiotic sensitivity

The susceptibility of the endophytes to 11 antibiotics, oxacilin (a penicillin), vancomycin (a glycopeptide), chloramphenicol (an amphionicol), meropenem (a carbapenem), streptomycin (an aminoglycoside), tetracycline (a tetracycline), gentamicin (another aminoglycoside), rifampicin (a macrolactam), ampicillin (another penicillins), ceftazidime (a third generation cephalosporin), and nalidixic acid (a quinolone) were evaluated as described by Passari et al. (2015). The analysis was performed considering the isolate sensitive (S) with an inhibition zone > 20 mm, intermediate (I) with an inhibition zone of 10–19.9 mm and resistant (R), if the inhibition zone was between 0.0–9.9 mm (Williams et al., 1989).

### Biological activity

#### Screening of culture conditions

Isolates were inoculated in 50 mL of SG medium (Shaaban et al., 2011), incubated for 3 days at 36°C and 180 rpm. Subsequently, 1 mL from the pre-culture was inoculated in SG and R5A media (100 mL) (Fernandez et al., 1998), and incubated for 10 days at two different temperatures, 28 and 36°C, and 180 rpm. The culture was filtered-off on Whatmann 4 filters, the water fraction was extracted with EtOAc (3 × 100 mL). The combined organics were evaporated *in vacuo* at 40°C and diluted in methanol at 10 mg/mL.

#### Antibacterial activity–disk diffusion assays

The antibacterial activity of crude extracts and the isolated compounds **1–9** was evaluated against methicillin-sensitive *Staphylococcus aureus* (MSSA) (ATCC 25923), methicillin-resistant *S. aureus* (MRSA) (BACHC-MRSA), *Pseudomonas aeruginosa* (ATCC 27853), *Candida albicans* (ATCC 10231), *Acinetobacter baumannii* (*BACHC-ABA*), *Klebsiella pneumoniae*, the producer of the enzyme *KPC* (*K. pneumoniae* carbapenemase) (BACHC-KPC), *Stenotrophomonas maltophilia* (BACHC-SMA), and *Enterobacter cloacae* a producer of the enzyme VIM (Verona integron-encoded metallo-β-lactamase) (BACHC-VIM). The bacteria were cultivated for 12 h at 37°C, and diluted according to the McFarland standard 0.5 scale. Each test organism was streaked on a sterile Mueller-Hinton agar plate with a cotton swab. Extracts were aliquoted in 100 μg amounts per 6 mm sterile filter disc. The discs were placed on plates and incubated for 24 h at 37°C. The diameter halos were measured in millimeters. As a positive control, a disc with a standard antibiotic with activity against each of the bacteria was used, and pure methanol was used as negative control (CLSI, 2015; Savi et al., 2015b).

#### MIC–minimum inhibitory concentration and MBC–minimum bactericidal concentration

Extracts from strain LGMB491 that showed high antibacterial activity were selected to determine the minimum inhibitory concentration. The MIC of extracts against the clinical pathogens was performed as described by Ostrosky et al. (2008) and CLSI. The minimum bactericidal concentration was determined as described by Soltani and Moghaddam (2014).

#### Statistical analyses

The statistical analysis was performed using analysis of variance (ANOVA) to compare extract effects to their respective controls. We also performed *Post-hoc* tests using Tukey's honest significant difference. All tests premises were fulfilled; the significance level used was 0.05 (α).

#### Large-scale fermentation, extraction and isolation

A large-scale fermentation (10 L) of strain LGMB491 was performed using SG culture medium at 36°C for 10 days. The culture was subjected to extraction with EtOAc (3 × v/v), and the combined organic layers were evaporated *in vacuo* at 40°C to yield 653 mg of crude extract. The crude extract was subjected to reverse phase C_18_ column chromatography (20 × 8 cm, 250 g), eluted with a gradient of H_2_O-MeOH (100:0–0:100) to produce fractions FI-FV. The single fractions were subjected to HPLC and Sephadex LH-20 (MeOH; 1 × 20 cm) purifications to yield compounds **1–9** in pure form (**Figure 9**, Figure S9). NMR spectra were measured using a Varian (Palo Alto, CA) Vnmr 400 (^1^H, 400 MHz; ^13^C, 100 MHz) spectrometer, δ-values were referenced to the respective solvent signals (CD_3_OD, δ_H_ 3.31 ppm, δ_C_ 49.15 ppm; DMSO-*d*_6_, δ_H_ 2.50 ppm, δ_C_ 39.51 ppm). HPLC-MS analyses were accomplished using a Waters (Milford, MA) 2695 LC module (Waters Symmetry Anal C_18_, 4.6 × 250 mm, 5 μm; solvent A: H_2_O/0.1% formic acid, solvent B: CH_3_CN/0.1% formic acid; flow rate: 0.5 mL min^−1^; 0–4 min, 10% B; 4–22 min, 10–100% B; 22–27 min, 100% B; 27–29 min, 100–10% B; 29–30 min, 10% B). HPLC analyses were performed on an Agilent 1260 system equipped with a photodiode array detector (PDA) and a Phenomenex C_18_ column (4.6 × 250 mm, 5 μm; Phenomenex, Torrance, CA). Semi-preparative HPLC was accomplished using Phenomenex (Torrance, CA) C_18_ column (10 × 250 mm, 5 μm) on a Varian (Palo Alto, CA) ProStar Model 210 equipped with a photodiode array detector and a gradient elution profile (solvent A: H_2_O, solvent B: CH_3_CN; flow rate: 5.0 mL min^−1^; 0–2 min, 25% B; 2–15 min, 25–100% B; 15–17 min, 100% B; 17–18 min, 100–25% B; 18–19 min, 25% B). All solvents used were of ACS grade and purchased from the Pharmco-AAPER (Brookfield, CT). Size exclusion chromatography was performed on Sephadex LH-20 (25–100 μm; GE Healthcare, Piscataway, NJ).

## Results

### Isolation of endophytic actinomycetes

From 2,988 fragments analyzed, 10 endophytic actinomycetes were isolated (Table 1), thus the isolation frequency was 0.34%. From the 10 isolates, 70% (*n* = 7) were isolated from the Abobral, and 30% (*n* = 3) from the São Bento region. Five isolates were obtained from stems, and five from leaf tissues of the plant (Table 1).

### Morphological identification

A great macro-morphological diversity was observed, with white, ivory-white, pink, brown, gray, orange, and yellow colony colors. Most of isolates showed abundant to moderate growth after 21 days of incubation, and six isolates showed abundant to moderate spore formation on ISP2 and ISP3 media. Isolates LGMB461 and LGMB465 showed high morphological similarity, and probably represent the same species (Table 1).

### Molecular analysis

Using a BLAST analysis in the GenBank database, the isolates were classified as eight genera: *Aeromicrobium, Williamsia, Microbacterium, Sphaerisporangium, Micrococcus, Microbispora, Actinomadura*, and *Streptomyces*. Each genus was analyzed in a separate phylogenetic tree based on Bayesian inference.

#### *Actinomadura* (LGMB466 and LGMB487)

**Figure 1:**
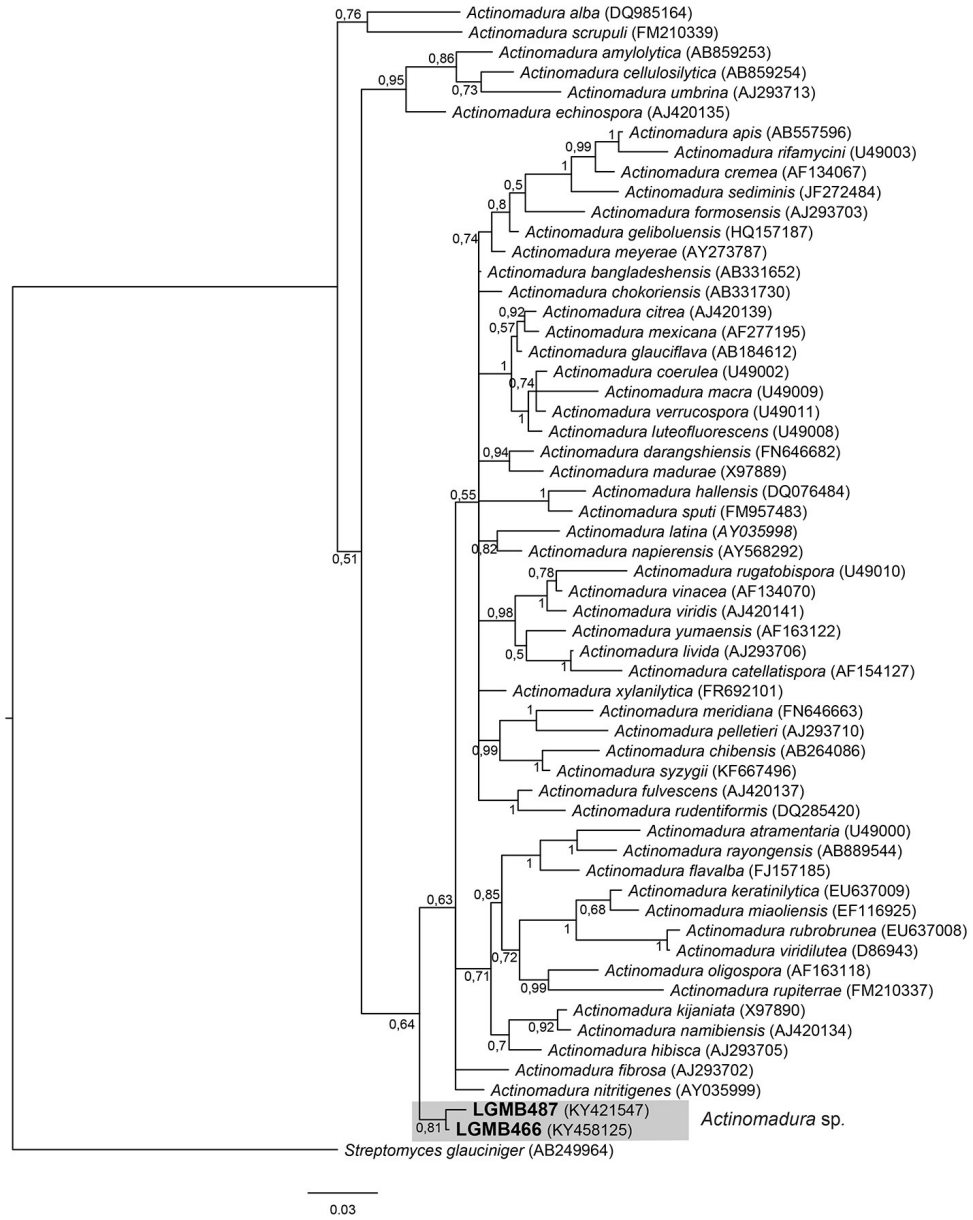
Bayesian phylogenic tree based on 16S rRNA gene of LGMB466, LGMB487, and the 53 type strain of *Actionomadura* genus. Values on the node indicate Bayesian posterior probabilities. The species *Streptomyces glauciniger* was used as out group. Scale bar indicates the number of substitutions per site.

The alignment consisted of strains LGMB466 and LGMB487, 55 type strains representative of *Actinomadura* genus, and *Streptomyces glauciniger* (AB249964) as out group taxa. The analysis comprises of 1,402 characters, 1,011 of these were conserved, 124 were parsimony informative and 131 were uninformative. Strains LGMB466 and LGMB487 showed high similarity among themselves (98.86%), and in the phylogenetic analysis these isolates did not cluster with any species from the *Actinomadura* genus (Figure 1, Table S1), and probably represent a new species.

#### *Aeromicrobium* (LGMB491)

**Figure 2:**
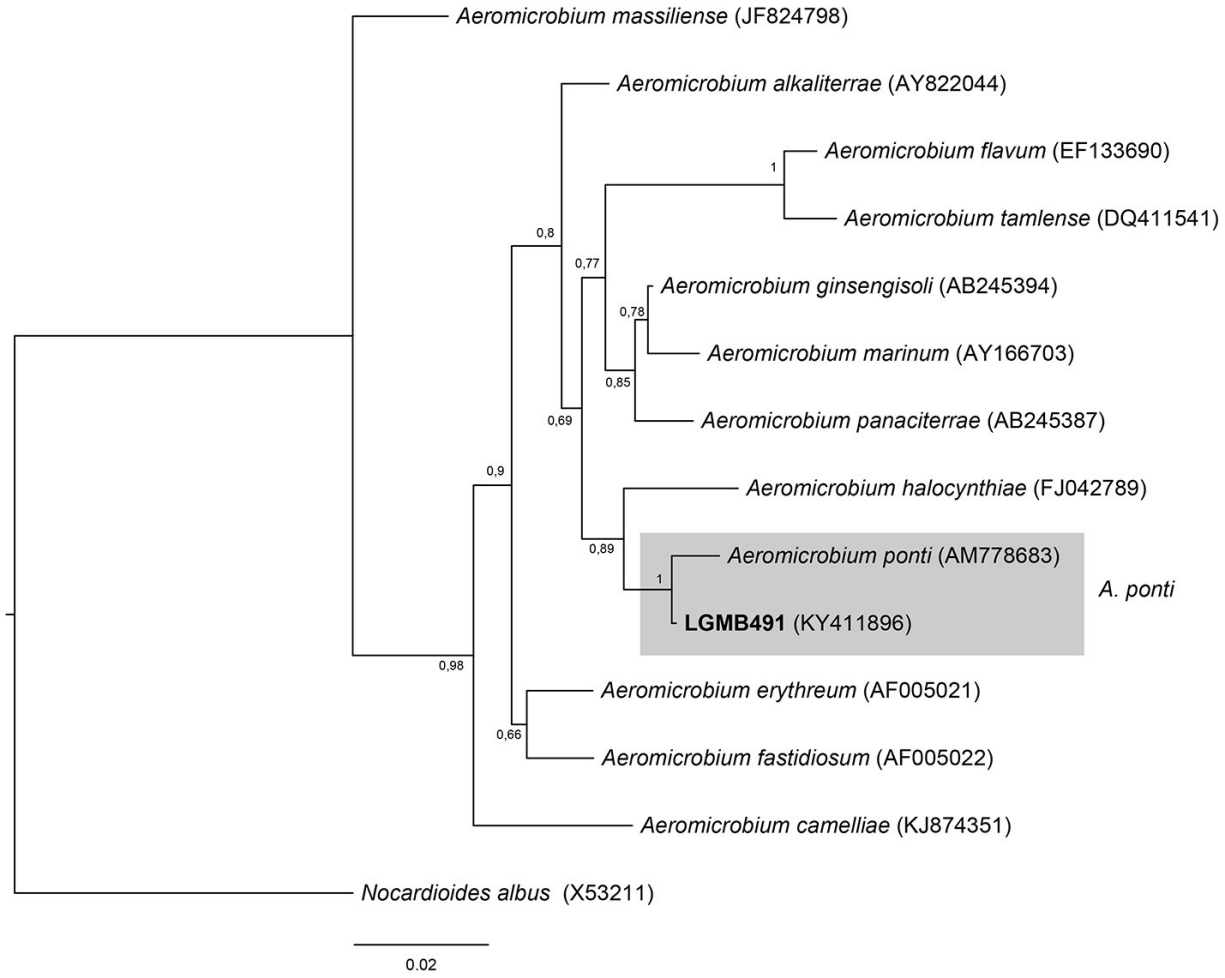
Bayesian phylogenic tree based on 16S rRNA gene of LGMB491 and the 12 type strain of *Aeromicrobium* genus. Values on the node indicate Bayesian posterior probabilities. The species *Nocardioides albus* was used as out group. Scale bar indicates the number of substitutions per site.

Strain LGMB491 was aligned with all type strains from the *Aeromicrobium* genus (12 species), and *Nocardioides albus* (X53211) was used as out group taxa. The alignment consisted of 1,336 characters, 1,164 of these were conserved, 89 were parsimony informative and 68 were uninformative. Based on this phylogenetic analysis, strain LGMB491 is close related to *Aeromicrobium ponti* (Figure 2), sharing high sequence similarity, 99.25 % (Table S2).

#### *Microbacterium* (LGMB471)

**Figure 3:**
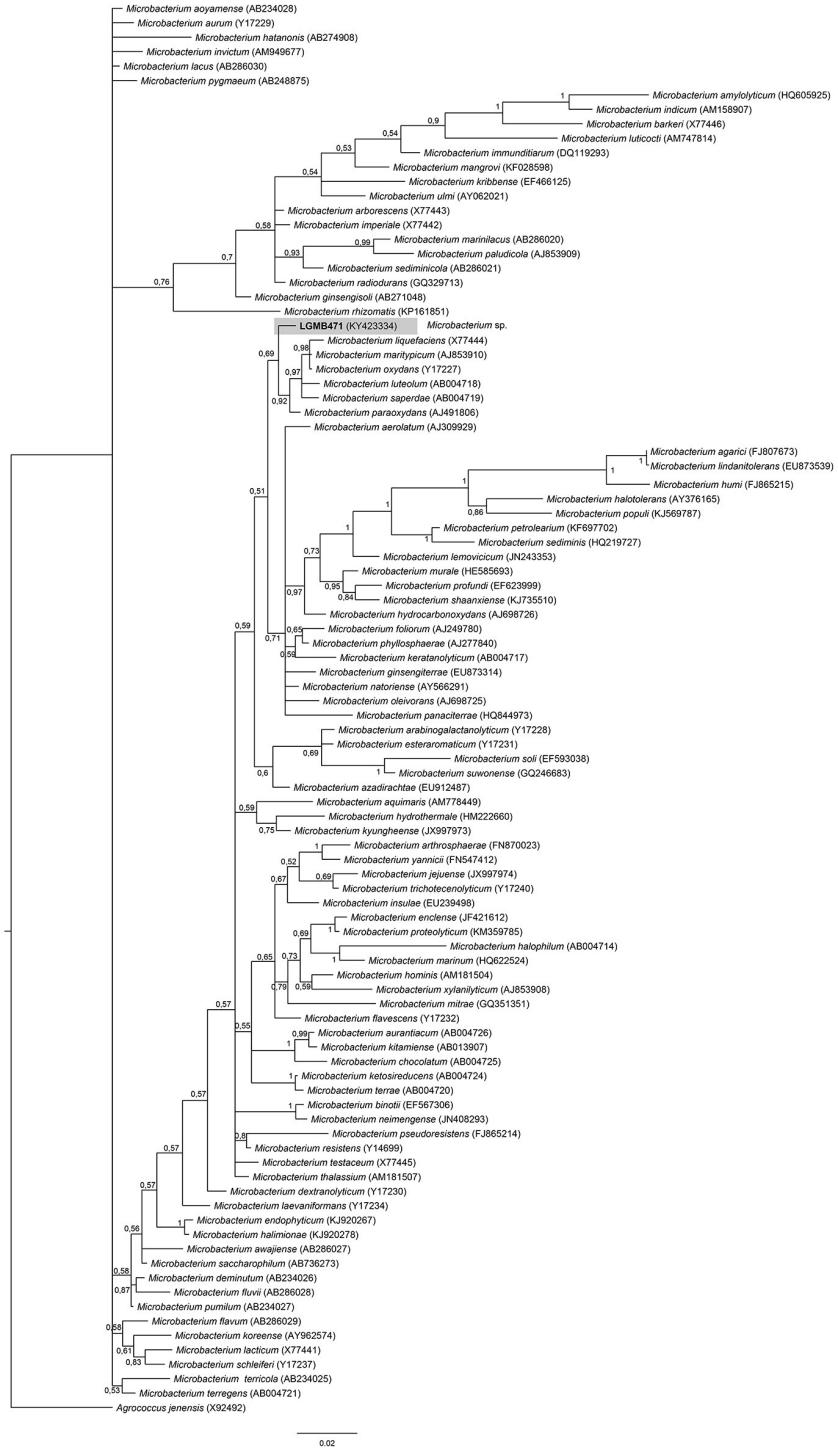
Bayesian phylogenic tree based on 16S rRNA gene of LGMB471 and the 94 type strain of *Microbacterium* genus. Values on the node indicate Bayesian posterior probabilities. The species *Agrococcus jenensis* was used as out group. Scale bar indicates the number of substitutions per site.

Strain LGMB471 was aligned with type strains from the *Microbacterium* genus, and *Agrococcus jenensis* (X92492) as out group taxa. The alignment comprised of 1,314 characters, of those 721 conserved sites, 122 were parsimony informative, and 57 uninformative. In the phylogenetic tree, isolate LGMB471 ended up in a single branch related to species *Microbacterium liquefaciens, Microbacterium maritypicum, Microbacterium oxydans, Microbacterium luteolum, Microbacterium saperdae, and Microbacterium paraoxydans* (Figure 3, Table S3).

#### *Microbispora* (LGMB461 and LGMB465)

**Figure 4:**
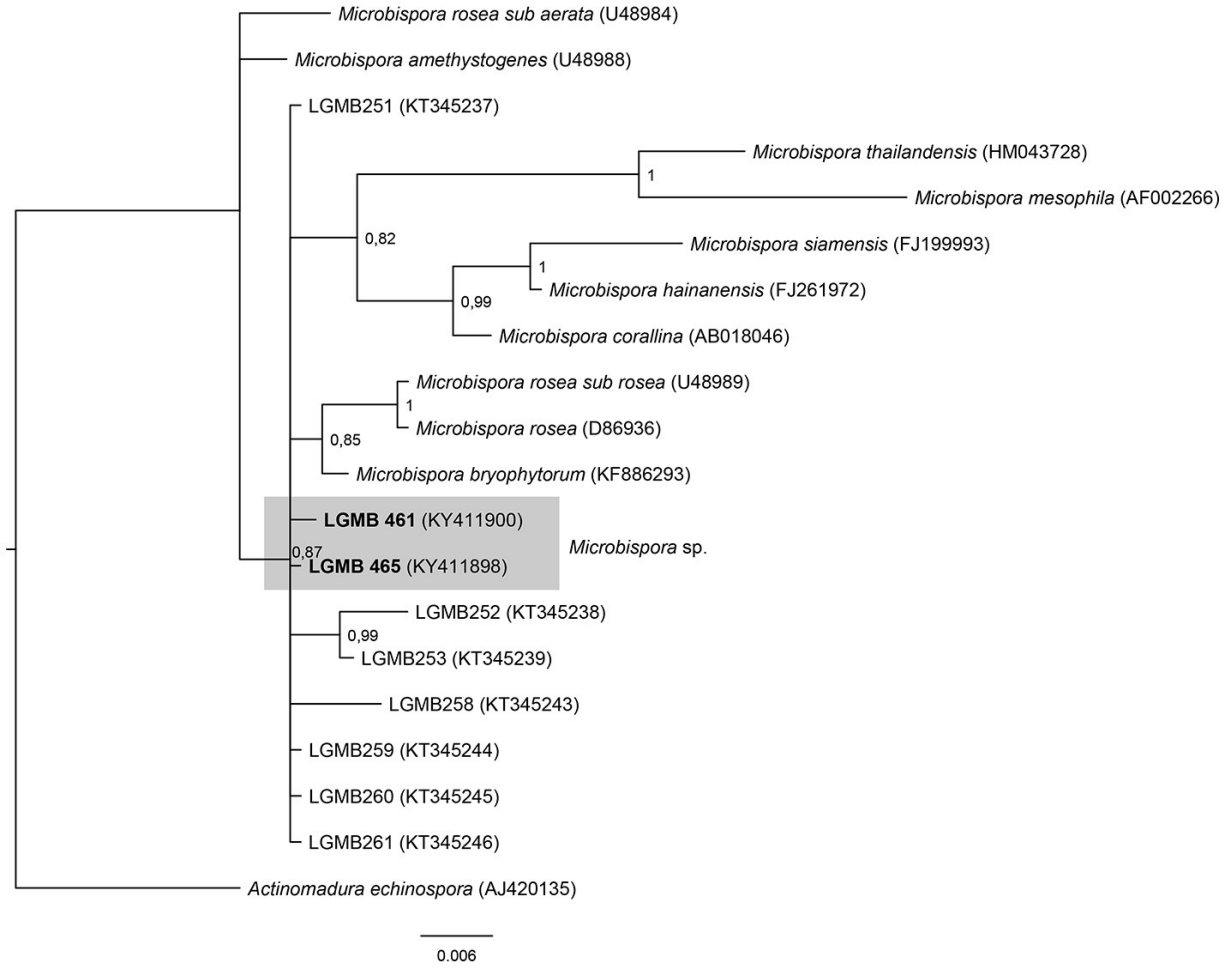
Bayesian phylogenic tree based on 16S rRNA gene of LGMB461, LGMB465, the 10 type strain of *Microbispora* genus, and 7 strains previously reported by Savi et al. (2016). Values on the node indicate Bayesian posterior probabilities. The species *Citricoccus parietis* was used as out group. Scale bar indicates the number of substitutions per site.

The analysis comprises of strains LGMB461 and LGMB465, 10 species accepted in *Microbispora* genus, and the isolates previously reported as *Microbispora* sp.1, *Microbispora* sp.2, and *Microbispora* sp.3 (Savi et al., 2016). *Actinomadura echinospora* (AJ420135) was used as out group taxa. The alignment consists of 1,371 characters, 1,309 of these were conserved, 33 were parsimony informative, and 29 uninformative. In the phylogenetic analysis strains LGMB461 and LGMB465 presented similarity with *Microbispora* sp.1 (LGMB259) with 99.84 and 100% f similarity, respectively (Figure 4, Table S4).

#### *Micrococcus* (LGMB485)

**Figure 5:**
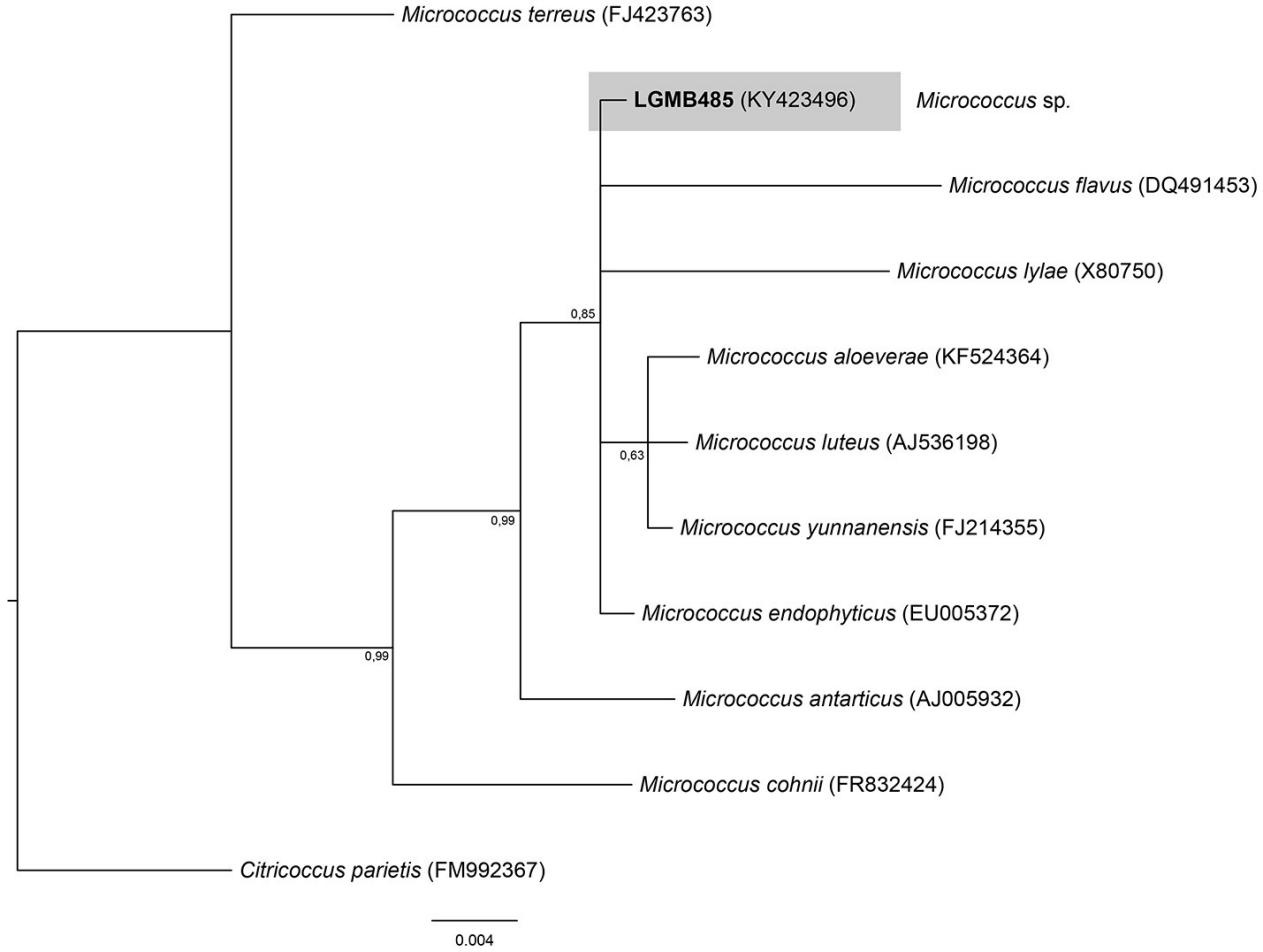
Bayesian phylogenic tree based on 16S rRNA gene of LGMB485 and the 9 type strain of *Micrococcus* genus. Values on the node indicate Bayesian posterior probabilities. The species *Citricoccus parietis* was used as out group. Scale bar indicates the number of substitutions per site.

The Bayesian analysis comprised of all *Micrococcus* type strains, strain LGMB485 and *Citricoccus parietis* (FM9923367) as out group taxa (Figure 5). The alignment consisted of 1,340 characters with 452 conserved sites, nine were parsimony informative and 19 uninformative. Since the sequences were very similar (Table S5) and the alignment had only nine parsimony informative sites, a species designation cannot be assigned, and isolate LGMB485 was identified as *Micrococcus* sp.

#### *Sphaerisporangium* (LGMB482)

**Figure 6:**
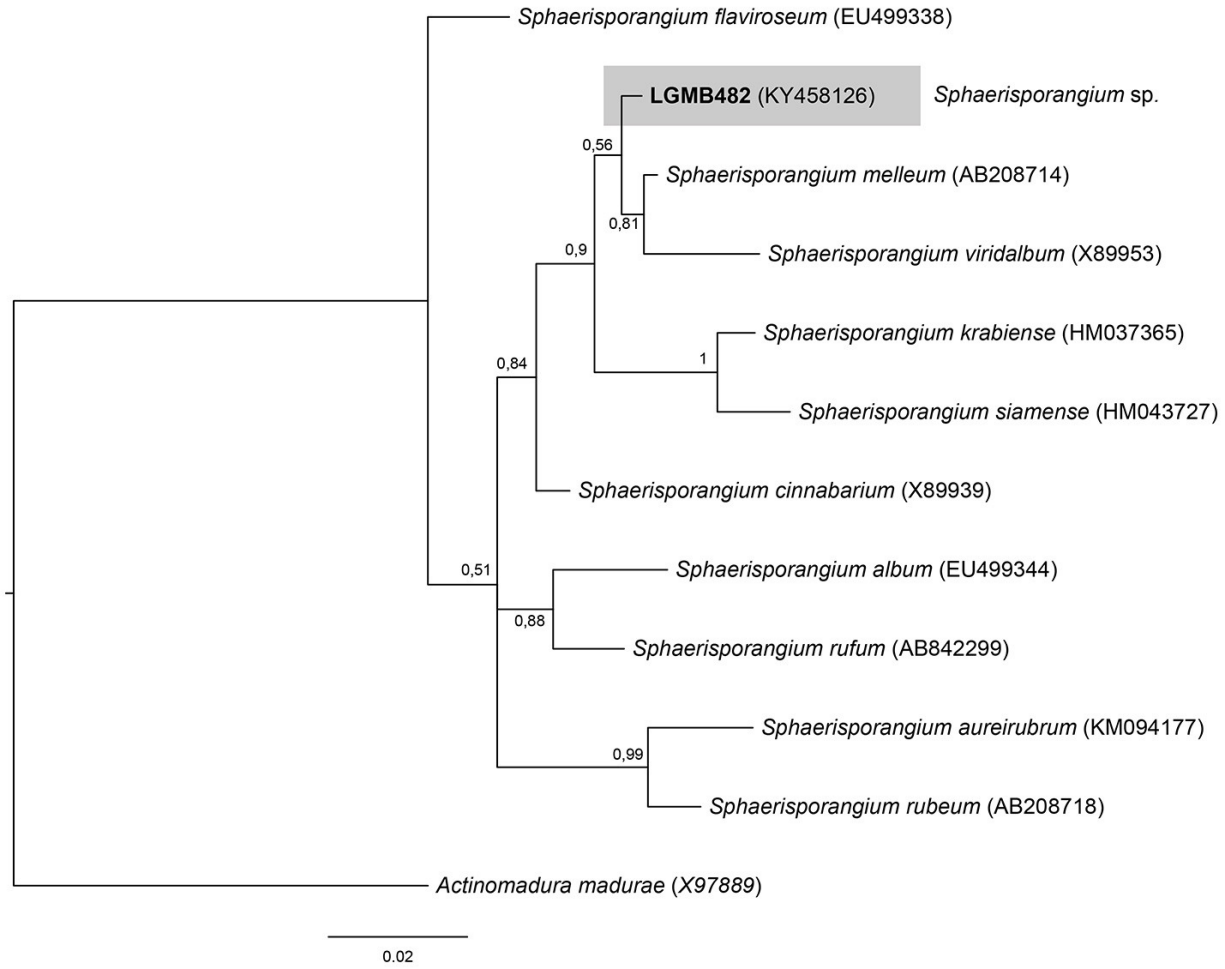
Bayesian phylogenic tree based on 16S rRNA gene of LGMB482 and the 10 type strain of *Sphaerisporangium* genus. Values on the node indicate Bayesian posterior probabilities. The species *Actinomadura madurae* was used as out group. Scale bar indicates the number of substitutions per site.

For the Bayesian analysis, the sequence from LGMB482 was aligned with strains of the *Sphaerisporangium* genus, and *Actinomadura madurae* (X97889) was used as out group taxa. The alignment consisted of 1,320 characters, 886 of these were conserved, 51 were parsimony informative and 47 were uninformative. Strain LGMB482 is closely related to *S. melleum* AB208714 (99.4% similarity) and *S. viridalbum* X89953 (97.89% similarity), however, it is in an isolated branch and may represent a new species of the *Sphaerisporangium* genus (Figure 6, Table S6).

#### *Streptomyces* (LGMB483)

**Figure 7:**
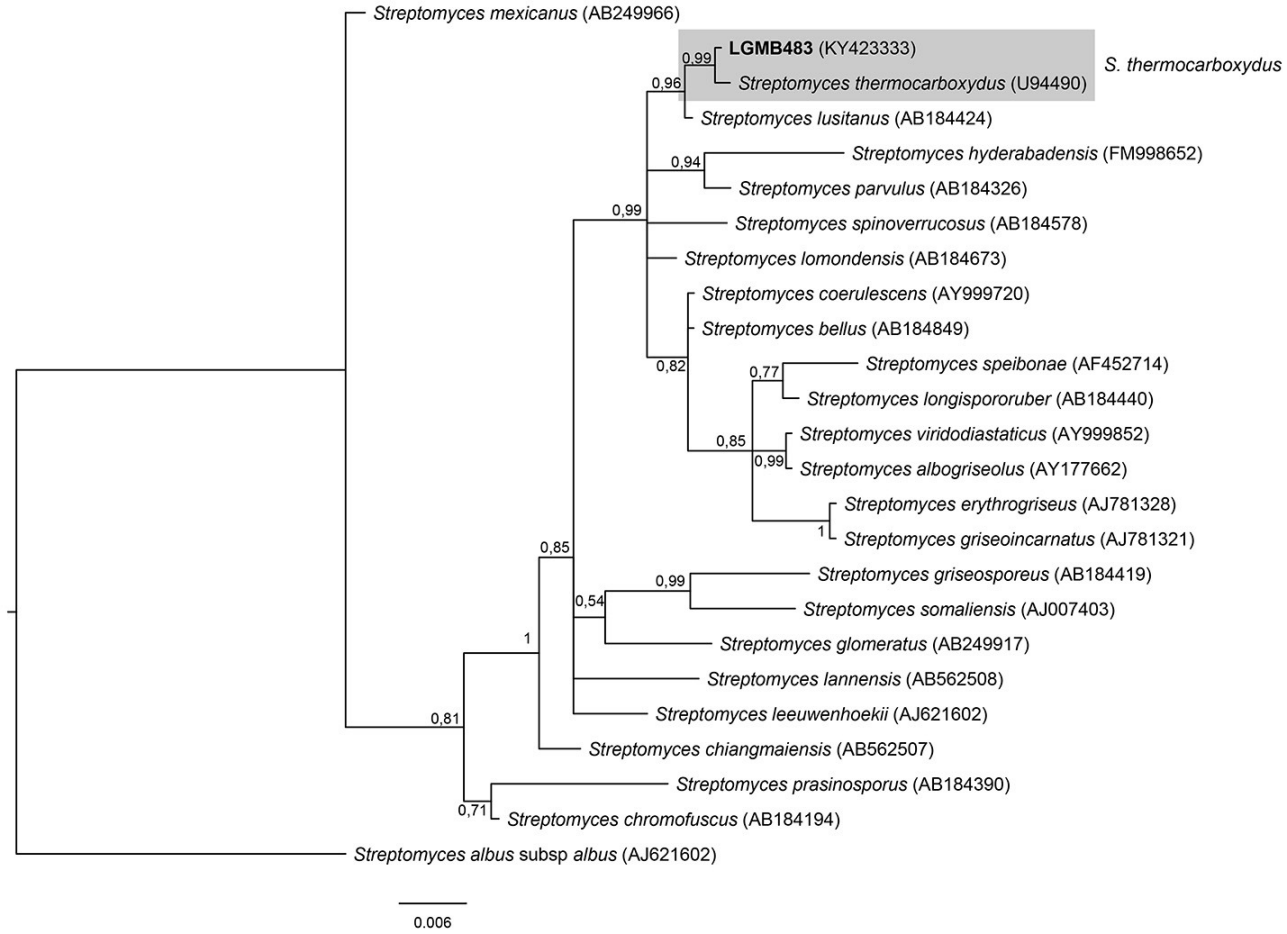
Bayesian phylogenic tree based on 16S rRNA gene of LGMB483 and the 33 type strain of *Streptomyces* genus. Values on the node indicate Bayesian posterior probabilities. The species *Streptomyces albus* subsp.*albus* was used as out group. Scale bar indicates the number of substitutions per site.

The phylogenetic analysis was performed using 23 type strains closely related with LGMB483; including *Streptomyces albus* subsp. *albus* (X53163) as out group taxa. The alignment consisted of 1,391 characters, with 1,291 conserved sites, 45 were parsimony informative, and 39 uninformative. In the phylogenetic tree, isolate LGMB483 grouped with *Streptomyces thermocarboxydus*, sharing 99.86% of similarity (Figure 7, Table S7), and thus we suggest this isolate may belongs to this species.

#### *Williamsia* (LGMB479)

**Figure 8:**
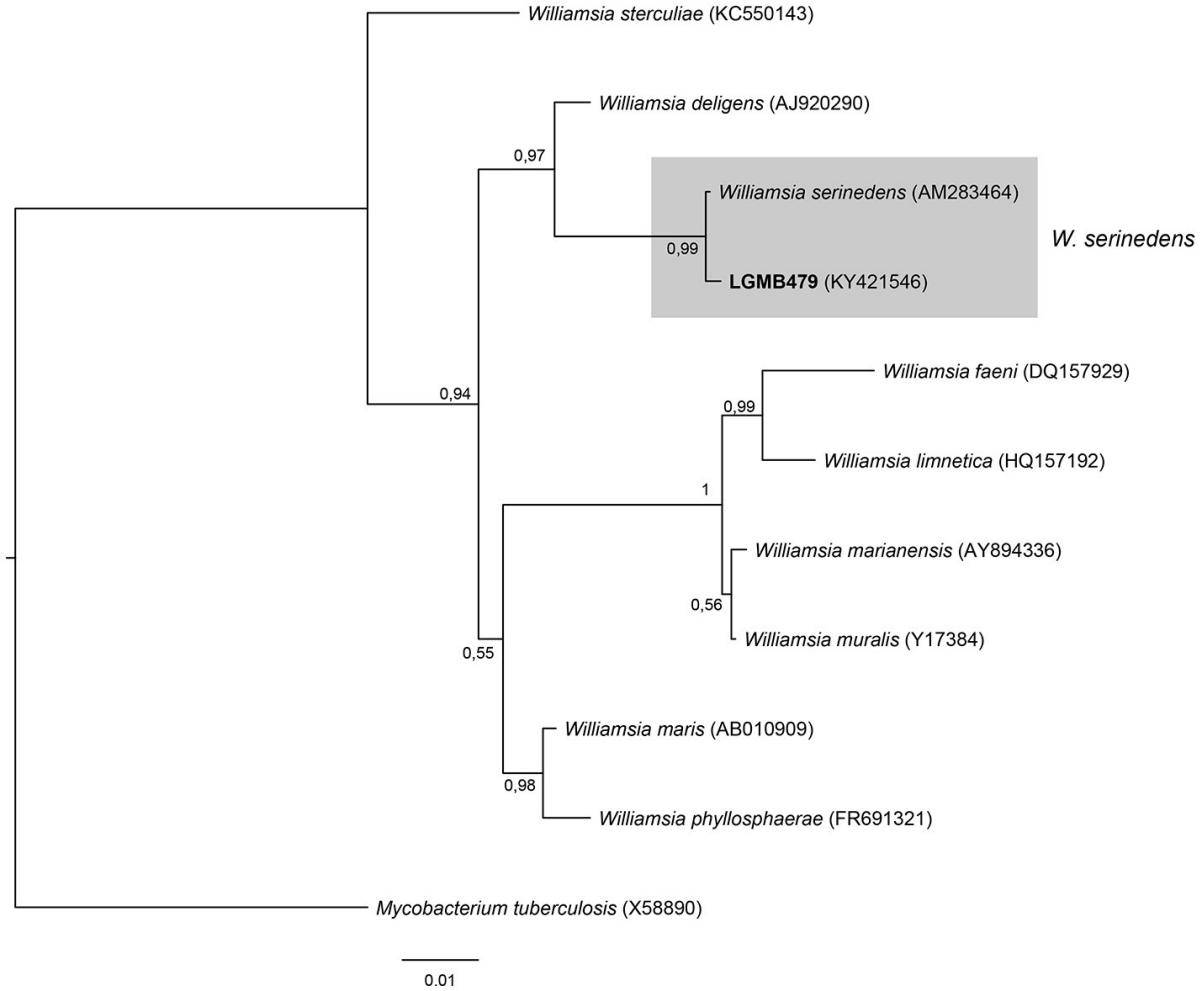
Bayesian phylogenic tree based on 16S rRNA gene of LGMB479 and the 9 type strain of *Willamsia* genus. Values on the node indicate Bayesian posterior probabilities. The species *Mycobacterium tuberculosis* was used as out group. Scale bar indicates the number of substitutions per site.

The analysis consists of 11 sequences, including all type strains of the *Williamsia* genus, the strain LGMB479, and *Mycobacterium tuberculosis* (X58890) was used as out group taxa. The alignment comprises of 1,346 characters, of these 1,185 were conserved, 81 were parsimony informative and 56 were uninformative. Strain LGMB479 was in the same clade with *Williamsia serinedens* (AM283464) (Figure 8) and share 99.85% sequence similarity (Table S8), and may belongs to this species.

### Antibiotic sensitivity test

In order to characterize the susceptibility profiles of the endophytes, 11 antibiotics with different mechanisms-of-action were utilized. Isolates were susceptible to vancomycin (80% sensitive and 20% intermediate), streptomycin (90% sensitive and 10% intermediate), tetracycline (70% sensitive and 30% intermediate), and gentamicin (80% sensitive and 20% intermediate). The two isolates of *Microbispora* sp. (LGMB461 and LGMB465) showed resistance to meropenem, and 90% of the isolates showed resistance to oxacillin, and nalidixic acid (Table 2).

**Table 2:** Antibiotic sensitivity pattern of endophytic actinomycetes.

	**Antibiotic sensitivity**										
**Strain/Genera**	**Oxa 1 μg**	**Van 30 μg**	**Clo 30 μg**	**Mer 10 μg**	**Est 10 μg**	**Tet 30 μg**	**Gen 10 μg**	**Rif 5 μg**	**Amp 10 μg**	**Caz 30 μg**	**Nal 30 μg**
*Actinomadura* sp. **LGMB466**	R	S	I	S	S	S	S	I	R	R	R
*Actinomadura* sp. **LGMB487**	S	I	R	S	S	S	S	S	I	S	S
*Aeromicrobium ponti* **LGMB491**	R	S	R	S	S	S	S	S	R	S	S
*Microbacterium* sp. **LGMB471**	R	S	I	S	S	S	S	I	R	R	R
*Microbispora* sp. **LGMB461**	R	S	R	R	S	S	S	I	R	R	R
*Microbispora* sp. **LGMB465**	R	S	R	R	S	S	I	I	R	R	R
*Micrococcus* sp. **LGMB485**	R	I	R	S	I	I	I	I	R	S	R
*Sphaerisporangium* sp**LGMB482**	R	S	I	S	S	S	S	I	R	R	R
*Streptomyces thermocarboxydus*. **LGMB483**	R	S	R	S	S	I	S	R	R	R	R
*Williamsia serinedens*. **LGMB479**	R	S	R	S	S	I	S	I	I	S	R

*Degree of susceptibility: >20 mm—Sensitive; 10–19.9 mm—intermediate; 0.0–9.9 mm resistant. Oxa, Oxacillin (1 μg/disc); Van, Vancomycin (30 μg/disc); Clo, Chloramphenicol (30 μg/disc); Mer, Meropenem (10 μg/disc); Est, Streptomycin (30 μg/disc); Tet, Tetracycline (30 μg/disc);.Gen, Gentamicin (10 μg/disc); Rif, Rifampicin (5 μg/disc); Amp, Ampicillin (10 μg/disc); Caz, Ceftazidime (30 μg/disc); Nal, Nalidixic acid (30 μg/disc)*.

### Antibacterial activity of crude extracts

All strains and culture conditions analyzed produced active extracts (Table 3, Table S9), however, the extract from LGMB491 (close related to *A. ponti*) cultured in SG medium at 36°C showed great antibacterial activity against *S. aureus* (22 mm) and MRSA (19.8 mm), and moderate activity against others clinical pathogens (Table 3, Figures S1–S8). The MIC and MBC of extract from LGMB491 against *S. aureus* and methicillin-resistant *S. aureus* were 0.02, and 0.04 mg/mL, respectively, and the MBC was 5 mg/mL for both bacteria (Table 4). In addition, the crude extract from LGMB491 had an MIC of 0.63 mg/mL against gram-negative bacteria associated with antibiotic resistance, *K. pneumoniae KPC, S. maltophilia*, and *E. cloacae* VIM, and a MIC of 0.31 mg/mL against *A. baumannii* and *P. aeruginosa*, respectively (Table 4).

**Table 3:** Antibacterial activity of the extracts from endophytic actinomycetes in two culture media (SG and R5A) and two temperatures (28°C and 36°C) against Clinical pathogens.

**Strain/Genera**	**Antimicrobial activity (inhibition zone in mm)**											
	***Staphylococcus aureus*** **(Figure S1)**				***Meticilin-resistant S. aureus*** **(Figure S1)**				***Escherichia coli*** **(Figure S2)**			
	**Medium SG**		**Medium R5A**		**Medium SG**		**Medium R5A**		**Medium SG**		**Medium R5A**	
	**28°C**	**36°C**	**28°C**	**36°C**	**28°C**	**36°C**	**28°C**	**36°C**	**28°C**	**36°C**	**28°C**	**36°C**
*Actinomadura* sp. LGMB466	9.75 ± 0.5	11.25 ± 0.5	11.25 ± 0.5	9 ± 0.8	9.5 ± 0.58	9.25 ± 0.5	9.5 ± 0.6	9.5 ± 0.6	12, 0	12.75 ± 0.5	12.75 ± 0.5	10.75 ± 0.5
*Actinomadura* sp. LGMB487	9.75 ± 0.5	11.75 ± 1	11.75 ± 1	9.5 ± 0.6	10.5 ± 1	9.5 ± 0.58	9.5 ± 0.6	9 ± 0.8	11.0	11.5 ± 0.6	12.25 ± 1	11.0
*Aeromicrobium ponti* LGMB491	20.5 ± 0.6	22 ± 1.3	19.25 ± 1.3	15.75 ± 1.7	24.2 ± 2.06	19.8 ± 0.5	11.5 ± 1.3	17.25 ± 1.7	11.5 ± 1	12 ± 0.8	10.5 ± 0.6	10.25 ± 0.5
*Microbacterium* sp. LGMB471	11.25 ± 1	9.5 ± 1	9.5 ± 1	11 ± 1.2	11.25 ± 0.96	8.25 ± 1.3	11.75 ± 1.5	9.25 ± 0.5	11.25 ± 0.5	12.25 ± 0.5	13 ± 0.8	11.0
*Microbispora* sp. LGMB461	8.75 ± 1.5	9.5 ± 1.0	9.5 ± 1.0	9.25 ± 1.5	9.75 ± 1.7	8.5 ± 1	10.75 ± 0.5	8.75 ± 0.5	12.75 ± 0.5	9.5 ± 0.6	12.25 ± 1	12.5 ± 0.6
*Microbispora* sp. LGMB465	10 ± 0.8	9.25 ± 0.5	9.25 ± 0.5	9.5 ± 0.6	9.5 ± 1	9.75 ± 0.96	8.0	11.5 ± 0.6	13.25 ± 1	11.5 ± 0.6	8.0	10.5 ± 0.6
*Micrococcus* sp. LGMB485	10.25 ± 0.5	12.5 ± 1.7	12.5 ± 1.7	10.5 ± 1.3	10 ± 0.82	11.2 ± 1.26	10 ± 1.4	8.75 ± 0.5	10.5 ± 0.6	10.75 ± 0.5	12.75 ± 0.5	10 ± 0.8
*Sphaerisporangium* sp. LGMB482	10.5 ± 1.3	10.25 ± 1.7	10.25 ± 1.7	11, 0	9.5 ± 0.58	10.5 ± 1.3	9.5 ± 0.6	9.5 ± 0.6	11 ± 0.8	9.75 ± 1	12 ± 0.8	11 ± 0.8
*S. thermocarboxydus* LGMB483	10.75 ± 1.5	10.0	10.0	11.25 ± 1.0	11.2 ± 0.96	11.2 ± 0.96	8.75 ± 1.5	8.5 ± 1	11.5 ± 1.3	11 ± 0.8	11, 0	8.25 ± 0.5
*Williamsia serinedens* LGMB479	12 ± 1.8	9.75 ± 2.2	9.75 ± 2.2	10.25 ± 0.5	12 ± 1.83	10.2 ± 0.96	10.0	10.25 ± 0.96	11.5 ± 0.6	9.5 ± 0.6	13.5 ± 1.3	12.25 ± 1
**Strain/Genera**	**Antimicrobial activity (inhibition zone in mm)**											
	***P.aeruginosa*** **(Figure S3)**				***A.baumanni*** **(Figure S4)**				***C. albicans*** **(Figure S5)**			
	**Medium SG**		**Medium R5A**		**Medium SG**		**Medium R5A**		**Medium SG**		**Medium R5A**	
	**28°C**	**36°C**	**28°C**	**36°C**	**28°C**	**36°C**	**28°C**	**36°C**	**28°C**	**36°C**	**28°C**	**36°C**
*Actinomadura* sp. LGMB466	9.25 ± 1	10 ± 0.8	9 ± 0.8	8.5 ± 1.3	10.5 ± 0.6	10 ± 0.8	10.5 ± 0.6	9.75 ± 1.3	9.75 ± 1	10.25 ± 0.5	9.75 ± 1.3	10 ± 0.8
*Actinomadura* sp. LGMB487	10.25 ± 1	7.75 ± 1	9.25 ± 0.5	7.75 ± 1	9.25 ± 0.9	10 ± 0.8	9.25 ± 1.9	9.5 ± 0.6	8 ± 0.8	7.5 ± 0.6	10.75 ± 1.9	7.75 ± 0.5
*Aeromicrobium ponti* LGMB491	10.5 ± 1	10.5 ± 2.9	9 ± 1.2	13 ± 1.8	12.5 ± 1	10.5 ± 2.1	9 ± 0.8	11.75 ± 1	10.25 ± 1.3	8.5 ± 1	8 ± 1.4	10.00
*Microbacterium* sp. LGMB471	9.0	10 ± 0.8	10.25 ± 1	10 ± 1.4	9.5 ± 0.6	10.5 ± 1.7	9.75 ± 0.5	9.25 ± 1	13.5 ± 0.6	10.75 ± 1.3	11.00	9.75 ± 0.5
*Microbispora* sp. LGMB461	8.25 ± 0.5	9.0	11.25 ± 1.5	9.0	8.5 ± 1	9.75 ± 0.5	9.00	10.75 ± 1	8.75 ± 1.5	9.25 ± 1	8.5 ± 0.6	8.75 ± 0.5
*Microbispora* sp. LGMB465	11 ± 1.4	10 ± 0.8	9.5 ± 0.6	10.5 ± 1	11.00	9 ± 0.8	10.25 ± 1	11.00	10.75 ± 0.5	10.25 ± 1	9.75 ± 0.5	11 ± 2
*Micrococcus* sp. LGMB485	9.0	10 ± 1.2	8.75 ± 1	9.0	10 ± 0.8	9.5 ± 1	9.25 ± 1.5	11.25 ± 0.5	11.25 ± 1	10.25 ± 0.5	7.5 ± 0.6	9.5 ± 1
*Sphaerisporangium* sp. LGMB482	10.5 ± 1.7	8.75 ± 0.5	10.25 ± 1	9.75 ± 1	11.25 ± 0.9	9.75 ± 1.7	10 ± 0.8	10 ± 0.8	11.75 ± 1.5	10.75 ± 1	13 ± 0.8	11.75 ± 1.5
*S. thermocarboxydus* LGMB483	11 ± 1.4	11.25 ± 1.5	7.5 ± 0.6	8.5 ± 0.6	9.75 ± 0.5	11 ± 1.4	7.75 ± 1	8.75 ± 0.9	10 ± 1.4	8.75 ± 0.5	7.75 ± 0.5	7.5 ± 0.6
*Williamsia serinedens* LGMB479	10.75 ± 2.1	10 ± 1.2	10 ± 1.2	9.5 ± 0.6	12.5 ± 1.9	9.5 ± 0.6	10.5 ± 1	10.75 ± 1.3	11 ± 2	11 ± 2	12.25 ± 1.7	10.25 ± 1.5
**Strain/Genera**	**Antimicrobial activity (inhibition zone in mm)**											
	***E. Cloacae*** **producer of** ***VIM*** **(Figure S6)**				***S. malthophilia*** **(Figure S7)**				***Klebissiella pneumoniae*** **producer of KPC (Figure S8)**			
	**Medium SG**		**Medium R5A**		**Medium SG**		**Medium R5A**		**Medium SG**		**Medium R5A**	
	**28°C**	**36°C**	**28°C**	**36°C**	**28°C**	**36°C**	**28°C**	**36°C**	**28°C**	**36°C**	**28°C**	**36°C**
*Actinomadura* sp. LGMB466	9.75 ± 0.5	10 ± 0.8	10.25 ± 0.5	10.5 ± 0.6	10.5 ± 1.3	11.75 ± 1	9.5 ± 0.6	9.25 ± 0.5	10.75 ± 0.5	10.5 ± 0.6	11.25 ± 1	10.25 ± 0.5
*Actinomadura* sp. LGMB487	11.25 ± 0.5	10.5 ± 0.6	10.75 ± 0.5	9.5 ± 0.6	11.25 ± 1	9 ± 0.8	11 ± 0.8	9.00	9.75 ± 1	8.25 ± 0.5	10.25 ± 0.5	8.5 ± 0.6
*Aeromicrobiumponti* LGMB491	10.75 ± 1	10.5 ± 1.3	12.25 ± 1.7	12.25 ± 1	13.5 ± 0.6	13.25 ± 1.5	9.00	12.25 ± 0.5	10 ± 0.8	10.75 ± 1	8.75 ± 0.5	10.5 ± 1
*Microbacterium* sp. LGMB471	11.25 ± 0.5	10.00	10.00	10.25 ± 0.5	12.5 ± 0.6	9.75 ± 0.5	10.75 ± 0.5	10.5 ± 0.6	11.75 ± 0.5	9.75 ± 1	11.25 ± 0.5	10 ± 0.8
*Microbispora* sp. LGMB461	8.75 ± 1	10.25 ± 1	10.5 ± 1.3	10.75 ± 1	10.25 ± 1.3	10.5 ± 1.3	11 ± 0.8	10.25 ± 0.5	11 ± 1.8	10.25 ± 0.5	9.25 ± 0.5	11.00
*Microbispora* sp. LGMB465	10.25 ± 1	9.5 ± 0.6	10.75 ± 0.5	10.25 ± 1.3	9.25 ± 1	9.75 ± 0.5	9.5 ± 1	9.5 ± 0.6	9.75 ± 0.9	11.5 ± 0.6	10 ± 0.8	9.25 ± 0.5
*Micrococcus* sp. LGMB485	12.25 ± 1.3	11.5 ± 0.6	10.75 ± 1	10 ± 1.4	11.75 ± 0.5	10.00	10.5 ± 0.6	10.5 ± 0.6	9.5 ± 0.6	11.25 ± 0.5	8.5 ± 0.6	10.00
*Sphaerisporangium* sp. LGMB482	11.5 ± 0.6	10.5 ± 0.6	10.75 ± 0.5	9.00	11.5 ± 1	10.75 ± 0.5	11.00	10.25 ± 1	12.75 ± 1.2	10.75 ± 0.5	12.00	9.75 ± 0.5
*S.thermocarboxydus* LGMB483	11.5 ± 0.6	12.00	10.25 ± 0.5	8.5 ± 0.6	11.5 ± 0.6	11.75 ± 1	9.75 ± 1	10.75 ± 1	11.00	12 ± 0.8	9.75 ± 0.9	8.5 ± 0.6
*Williamsia serinedens* LGMB479	10.75 ± 0.5	10.5 ± 0.6	11.25 ± 0.5	9.25 ± 0.5	12.00	12.25 ± 1	12 ± 1.2	11.25 ± 1	12.75 ± 1.2	12.25 ± 1	12 ± 0.8	12.25 ± 1

*Mean (±SD)*.

**Table 4:** Minimum Inhibitory and Minimum Bactericidal Concentrations of the extract from strain *Aeromicrobium ponti* LGMB491.

**Microrganism**	**MIC (mg/mL)**	**MBC**
Methicillin-sensitive *S. aureus* (MSSA)	0.02	5.0
Methicillin-resistant *S. aureus* (MRSA)	0.04	5.0
*Acinetobacter baumannii*	0.31	0.63
*Pseudomonas aeruginosa*	0.31	0.63
*Enterobacter cloacae* producer of VIM	0.63	1.25
*Klebsiella pneumoniae* producer of KPC	0.63	1.25
*Stenotrophomonas maltophilia*	0.63	1.25

### Structure determination of secondary metabolites from strain LGMB491

**Figure 9:**
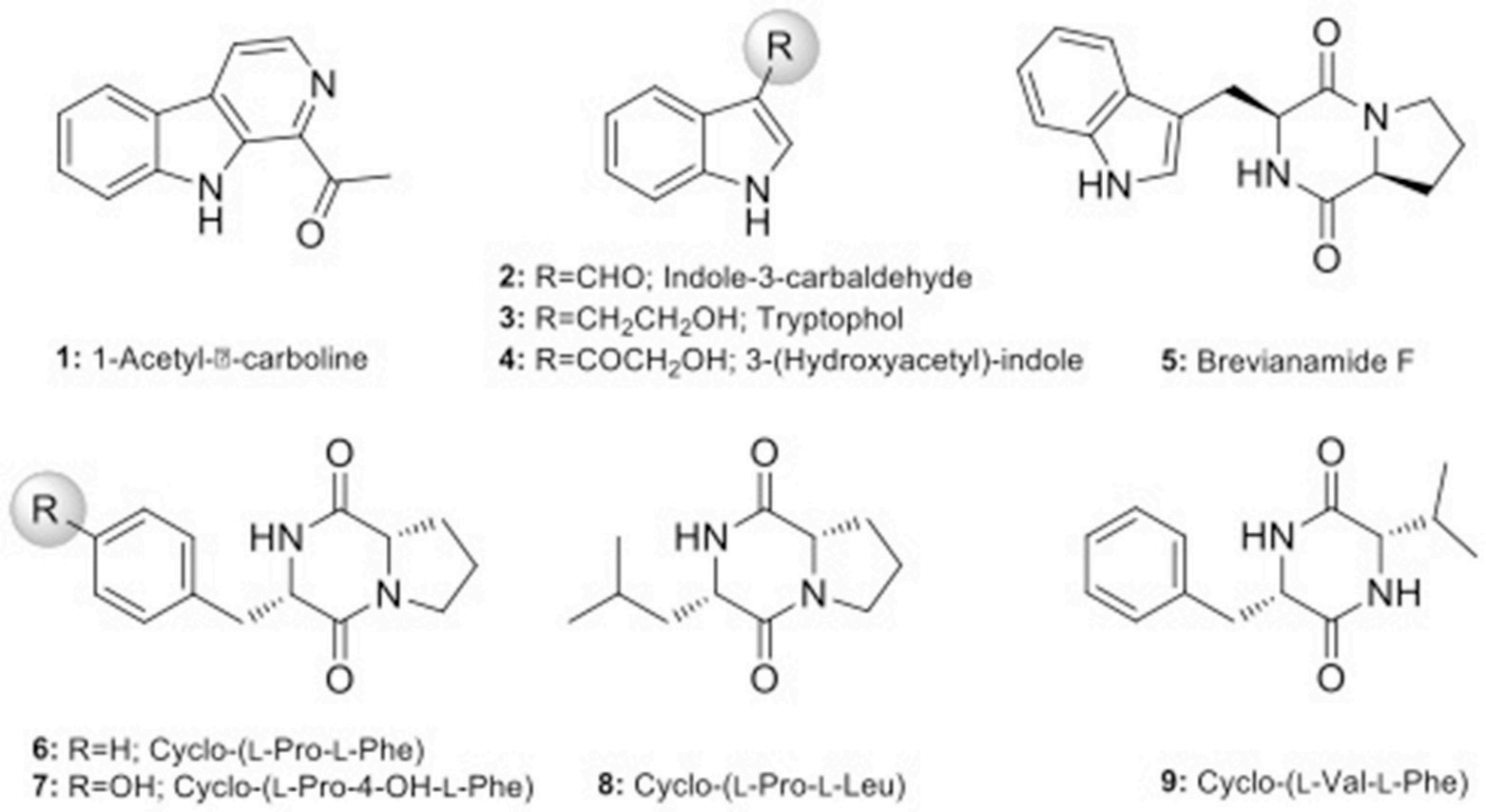
Chemical structure of compounds isolated from strain *Aeromicrobium ponti* LGMB491.

Scale-up fermentation of strain LGMB491 (10 L) using SG medium, followed by extraction afforded 653 mg of crude extract. Fractionation, isolation and purification of the obtained extract using various chromatographic techniques resulted in compounds **1–9** in pure forms (Figure S9). Thorough analyses of the HPLC/UV, ESIMS and NMR spectroscopy data (Figure S10–S43), and by comparison with literature data (Laatsch, 2012), the compounds were identified as 1-acetyl-β-carboline (**1**) (Shaaban et al., 2007; Savi et al., 2015b), indole-3-carbaldehyde (**2**) (Zendah et al., 2012; Savi et al., 2015b), tryptophol (**3**) (Rayle and Purves, 1967), 3-(hydroxyacetyl)-indole (**4**) (Zendah et al., 2012), brevianamide F (**5**) (Shaaban, 2009), cyclo-(L-Pro-L-Phe) (**6**) (Barrow and Sun, 1994), cyclo-(L-Pro-L-Tyr) (**7**) (Barrow and Sun, 1994), cyclo-(L-Pro-L-Leu) (**8**) (Yan et al., 2004), and cyclo-(L-Val-L-Phe) (**9**) (Pickenhagen et al., 1975) (Figure 9). In order to determine the compounds responsible for the biological activity observed for the crude extract of strain LGMB491, we evaluated the antibacterial activity of compounds **1–9** against *S. aureus* and methicillin-resistant *S. aureus*. 1-Acetyl-β-carboline (**1**) showed an equivalent activity as the antibiotic methicillin against *S. aureus*, however, different from this antibiotic, compound **1** also showed activity against MRSA (Table 5). In addition, compounds **2**, **4–6** also showed moderate activity against both MSSA and MRSA.

**Table 5:** Inhibition zone (mm) growth of methicillin-sensitive *Staphylococcus aureus* (MSSA) and methicillin-resistant *S. aureus* (MRSA) of compounds **1**–**9** (100 μg/disk).

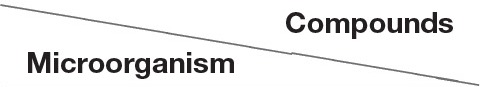	**1**	**2**	**3**	**4**	**5**	**6**	**7**	**8**	**9**	**Methicillin**
MSSA	18	10	10	10	11	10	–	–	–	20
MRSA	15	9	–	8	9	9	–	–	–	–

## Discussion

### Endophytes isolation and identification

Actinomycetes from medicinal plants are the source of several secondary metabolites with biological activity (Qin et al., 2015; Savi et al., 2015b), and their metabolites may be associated with the medicinal properties of the plant host (Kusari et al., 2013; Santos et al., 2015). We explored the endophytes from the medicinal plant *V. divergens*, in order to catalog the species richness and biological properties. A low frequency of isolation (0.34%), compared with the isolation of terrestrial actinomycetes, was observed, in agreement with literature data (Passari et al., 2015). However, despite the lower isolation frequency a higher richness of genera was observed (Passari et al., 2015; Saini et al., 2016). We reported for the first time the isolation of strains close related to the species *A. ponti* (LGMB491) and *Williamsia serinedens* (LGMB479) as endophytes. *A. ponti* was originally isolated from seawater (Lee and Lee, 2008), and has been found in this environment (Jiang et al., 2010; Claverias et al., 2015). *W. serinedens* was first isolated from an oil-contaminated soil sample and it is common isolated from different types of soil (Yassin et al., 2007). In addition, species *S. thermocarboxydus* was isolated from soil (Kim et al., 2000), and was recently described as endophyte from a medicinal plant in India (Passari et al., 2015). Based on the 16S rRNA phylogenetic analysis we suggest that strains LGMB471 and LGMB482 may represent new species within the *Microbacterium* and *Sphaerisporangium* genera, respectively (Figures 3, 6), and isolates LGMB466 and LGMB487 seem to be a new species within the *Actinomadura* genus (Figure 1). Isolates LGMB461 and LGMB465 belong to genus *Microbispora*, and showed high sequence similarity with strains belonging to *Microbispora* sp.1 group, previously isolated from *V. divergens* (Savi et al., 2016). However, sequencing others genes than 16S rRNA, and DNA-DNA hybridization would be required for species description (Meyers, 2014). *Microbacterium, Sphaerisporangium*, and *Micrococcus* species are common associated with medicinal plants in different regions, and climate conditions (Kim et al., 2000; Kamil et al., 2014; Xing et al., 2015). However, none of these has been isolated from wetland regions. Savi et al. (2015a) performed the first report about actinomycetes from the medicinal plant *V. divergens*. However, despite the higher number of isolates, the authors then just identified three genera as endophytes from this plant, *Microbispora, Micromonospora*, and *Streptomyces*. In addition to those genera previously mentioned (*Microbispora* and *Streptomyces*) we isolated species belonging to *Actinomadura, Aeromicrobium, Microbacterium, Sphaerisporangium, Micrococcus*, and *Williamsia* (Figures 1–8), thereby significantly increasing the knowledge regarding endophytes from *V. divergens*.

### Antibiotic sensitivity assay

In order to characterize the susceptibility profile as well as to suggest antibiotics to be used in actinomycete isolation, we evaluated the susceptibility profile of endophytes. We detected significant resistance to antibiotics oxacillin and nalidixic acid, only strain *Actinomadura* LGMB487 was sensitive to both compounds (Table 2). Nalidixic acid is the antibiotic used to inhibit bacterial growth during actinomycete isolation, however, even with the use of this compound, the presence of contaminating bacteria was common (Baskaran et al., 2011; Kadiri et al., 2014). Therefore, based on the high resistance to oxacillin observed in this study, we suggest the use of this antibiotic to inhibit bacterial growth during the isolation of actinomycetes. Strains LGMB466 and LGMB487, both characterized as *Actinomadura* sp., showed a complete different sensitivity pattern: strain LGMB487 was resistant only to chloramphenicol, and LGMB466 showed resistance to four antibiotics, and intermediate resistant to chloramphenicol, and rifampicin, suggesting that the resistance profile of isolates is not associated with the intrinsic factors of *Actinomadura* genus. The resistance observed in these strains can result from the presence of plasmids, which contributes to the well-known problem of antibiotic resistance (Wintersdorff et al., 2016). In addition, vancomycin, streptomycin, tetracycline, and gentamicin were previously reported from actinomycetes (Gonzalez and Spencer, 1998; Chopra and Roberts, 2001; Levine, 2006; Zumla et al., 2013), however, all strains evaluated here showed some sensitivity level to these antibiotics, which suggest that these compounds are not present as secondary metabolites from our isolates.

### Biological activity and secondary metabolites identification

All isolates and conditions analyzed produced active secondary metabolites, ratios superior than observed in previous studies (Higginbotham and Murphy, 2010; Passari et al., 2015; Tonial et al., 2016), suggesting the high biotechnological potential of the evaluated strains. This may be related to the culture conditions used to obtain the secondary metabolites. Extracts from LGMB491 (close related to *A. ponti*) showed great activity against MRSA, with inhibition zones higher than caused by vancomycin, the clinical antibiotic used for the treatment of this resistant bacterium (Table 3). In addition, extracts from strain LGMB491 also had considerable MIC, and MBC values against *S. aureus*, MRSA, *K. pneumoniae* KPC, *S. maltophilia, A. baumannii, P. aeruginosa*, and *E. cloacae* VIM. These data suggest the presence of metabolites with broad spectrum activity (Smith et al., 2011). Compounds with broad spectrum activity are required to treat multidrug resistant pathogens, such as MRSA, *S. maltophilia, P. aeruginosa*, and *A. baumannii* (Bonomo and Szabo, 2006; Çıkman et al., 2016), bacteria that are considered one of the most urgent issues in modern healthcare (Paulus et al., 2017). Therefore, due to the good activity observed, and the absence of studies about metabolites with biological activity from *A. ponti* species, we decided to characterize the major compounds produced by strain LGMB491. From the nine secondary metabolites isolated, 1-acetyl-β-carboline (**1**) turned out to be the compound responsible for the antibacterial activity of the LGMB491 extract. The compound displayed high activity against the MRSA (Table 5). β-carbolines are normally isolated from plants with a large spectrum of biological activity (Lee et al., 2013). Savi et al. (2015b) reported the production of four β-carbolines by the *Microbispora* sp. 1 also isolated from *V. divergens*. The authors isolated as the major metabolite the compound 1-vinyl-β-carboline-3-carboxylic acid, and attributed the vinyl chain as the likely responsible structural feature causing the antibacterial activity of this natural product. However, 1-acetyl-β-carboline (**1**), found during this study, showed also high biological activity, which is unlikely associated with the acetyl chain in position 1. Several studies demonstrated great activity of compound **1** against MRSA, and suggest the use of this compound for an effective treatment of this resistant bacterium (Shin et al., 2010; Lee et al., 2013). In addition to 1-acetyl-β-carboline (**1**), compounds **2**–**6** displayed moderate antibacterial activity, and may act synergistically with compound (**1**), contributing for the activity observed. Brevianamide F (**5**), an alkaloid, was isolated for the first time from *Penicillium brevicompactum* (Birsh and Wright, 1969), and has nematocidal (Shiomi and Omura, 2004), anti-inflammatory (Rand et al., 2005), and antibacterial activity against methicillin-sensitive and resistant *S. aureus* (Kumar et al., 2014; Alshaibani et al., 2016). Cyclo-(L-Pro-L-Phe) (**6**) is a diketopiperazine, i.e., a member of these cyclic dipeptides commonly isolated from microorganisms that have been associated with antimicrobial activity, and plant growth regulation (Zhang et al., 2013; Kalinovskaya et al., 2017). Interesting, several diketopiperazines, including cyclo-(L-Pro-L-Phe), were previously isolated from *Aspergillus fumigatus* from a soil sample of the Pantanal, and showed high antibacterial activity against *S. aureus* (Furtado et al., 2005), which supports the idea of synergism of the compounds produced by strain LGMB491. The indoles isolated from strain LGMB491 are commonly produced by plants and endophytic microorganism (Braga et al., 2016). 3-(Hydroxyacetyl)-indole (**4**) showed a broad-spectrum antibacterial activity against methicillin-resistant *S. aureus*, and against vancomycin-sensitive or resistant *Enterococci*, attributed to disruption of cell membrane (Sung and Lee, 2007). In plants, indole-3-carbaldehyde (**3**) is associated with the innate immunity to microbial pathogen infections (Stahl et al., 2016). This compound was also produced by *Microbispora* sp. 1 previously isolated from the medicinal plant *V. divergens* (Savi et al., 2015b). Some studies suggested that indole compounds play an important role in plant-microorganism interaction and plant defense (Gamir et al., 2012; Lin and Xu, 2013; Jeandet et al., 2014).

## Conclusion

In this study, we increased the knowledge regarding the endophytic community of the medicinal plant *V. divergens*, through the isolation of rare actinomycetes, some of which were never described as endophytes. We identified for the first time some secondary metabolites produced by one strain close related to the species *A. ponti*, and demonstrated that this species is able to produce indoles, β-carbolines, brevianamide, and diketopiperazines. Future studies to evaluate the potential of these compounds in animal models are required to better understand the potential of compound 1-acetyl-β-carboline as an alternative to treat MRSA infections. Our results indicate that actinomycetes from *V. divergens* have biotechnological potential as producer of bioactive compounds.

## Author contributions

All the authors contributed to the experimental design of the work; as well as to the acquisition, analysis, and interpretation of the obtained results; moreover, all the authors contributed to the writing and the critical revision of the manuscript.

### Conflict of interest statement

The authors declare that the research was conducted in the absence of any commercial or financial relationships that could be construed as a potential conflict of interest.
