# Supplementary material for: Antibacterial Activity of Endophytic Actinomycetes Isolated from the Medicinal Plant Vochysia divergens (Pantanal, Brazil)
Source: Front Microbiol. 2017 Sep 6;8:1642. doi: 10.3389/fmicb.2017.01642 (PMC5592219; doi:10.3389/fmicb.2017.01642)
Supplement: Supplementary file 1 [file DataSheet1.DOCX]

**Supplementary Information**

**Antibacterial activity of endophytic actinomycetes isolated from the medicinal plant *Vochysia divergens* (Pantanal, Brazil)**

Francielly Maria Wilke Ramos Gos^1#^, Daiani Cristina Savi^2#^, Khaled A. Shaaban^3,4#^, Jon S. Thorson^3,4^, Rodrigo Aluizio^2^, Yvelise Maria Possiede^5^, Jürgen Rohr^3*^ and Chirlei Glienke^2*^

^1^Federal University of Paraná, Department of Basic Pathology, Curitiba, Brazil. ^2^Federal University of Paraná, Department of Genetics, Box 19031, 81531–990, Curitiba, PR, Brazil. ^3^Department of Pharmaceutical Sciences, College of Pharmacy, University of Kentucky, Lexington, Kentucky 40536, United States. ^4^Center for Pharmaceutical Research and Innovation, College of Pharmacy, University of Kentucky, Lexington, Kentucky 40536, United States. ^5^Federal University of Mato Grosso do Sul, Department of Biology, Box: 79070900, Campo Grande, MS, Brazil

^#^Authors contributed equally to this work.

*Corresponding author: Chirlei Glienke, +5533611562, chglienke@gmail.com; Jürgen Rohr, +1 859 323 5031, jrohr2@email.uky.edu;

**Running title:** Antibacterial activity of endophytic actinomycetes

| **Contents** | **Page** | |
| --- | --- | --- |
| **Figure S1-**Inhibition zone (mm) of Methicillin-resistant *S. aureus (*MRSA) by extracts from endophytic actinomycetes in two media (SG and R5A) at two temperatures (28°C and 36°C). | | 3 |
| **Figure S2-**Inhibition zone (mm) of *Escherichia coli* by extracts from endophytic actinomycetes in two media (SG and R5A) at two temperatures (28°C and 36°C). | | 4 |
| **Figure S3-**Inhibition zone (mm) of *Pseudomonas aeruginosa* by extracts from endophytic actinomycetes in two media (SG and R5A) at two temperatures (28°C and 36°C) | | 4 |
| **Figure 4-**Inhibition zone (mm) of *Acinetobacter baumannii* by extracts from endophytic actinomycetes in two media (SG and R5A) at two temperatures (28°C and 36°C). | | 5 |
| **Figure S5-**Inhibition zone (mm) of *Candida albicans* by extracts from endophytic actinomycetes in two media (SG and R5A) at two temperatures (28°C and 36°C). | | 5 |
| **Figure S6-**Inhibition zone (mm) of *Enterobacter cloacae* producer enzyme VIM (Verona integron-encoded metallo-β-lactamase) by extracts from endophytic actinomycetes in two media (SG and R5A) at two temperatures (28°C and 36°C). | | 6 |
| **Figure S7-**Inhibition zone (mm) of *Stenotrophomonas maltophilia* by extracts from endophytic actinomycetes in two media (SG and R5A) and two temperatures (28°C and 36°C). | | 6 |
| **Figure S8-**Inhibition zone (mm) of *Klebissiella pneumoniae* producer enzyme KPC (*K. pneumoniae* carbapemase) by extracts from endophytic actinomycetes in two media (SG and R5A) at two temperatures (28°C and 36°C) | | 7 |
| **Firure S9-** Work-up scheme of the metabolites produced by LGMB491 | | 8 |
| **Figure S10-** HPLC/UV analyses of 1-acetyl-β-carboline (1) | | 9 |
| **Figure S11-** HPLC/MS analyses of 1-acetyl-β-carboline (1) | | 10 |
| **Figure S12-** ^1^H NMR spectrum (DMSO-*d*_6_, 400 MHz) of 1-acetyl-β-carboline (**1**) | | 11 |
| **Figure S13-** ^13^C NMR spectrum (DMSO-*d*_6_, 100 MHz) of 1-acetyl-β-carboline (**1**) | | 12 |
| **Figure S14-** HPLC/UV analyses of indole-3-carbaldehyde (**2**) | | 13 |
| **Figure S15-** HPLC/MS analyses of indole-3-carbaldehyde (**2**) | | 14 |
| **Figure S16-** ^1^H NMR spectrum (CD_3_OD, 400 MHz) of indole-3-carbaldehyde (**2**) | | 15 |
| **Figure S17-** ^13^C NMR spectrum (CD_3_OD, 100 MHz) of indole-3-carbaldehyde (**2**) | | 16 |
| **Figure S18-** HPLC/UV analyses of tryptophol (**3**) | | 17 |
| **Figure S19-** HPLC/MS analyses of tryptophol (**3**) | | 18 |
| **Figure S20-** ^1^H NMR spectrum (CD_3_OD, 400 MHz) of tryptophol (**3**) | | 19 |
| **Figure S21-** ^13^C NMR spectrum (CD_3_OD, 100 MHz) of tryptophol (**3**) | | 20 |
| **Figure S22-** HPLC/UV analyses of 3-(hydroxyacetyl)-indole (**4**) | | 21 |
| **Figure S23-** HPLC/MS analyses of 3-(hydroxyacetyl)-indole (**4**) | | 22 |
| **Figure S24-** ^1^H NMR spectrum (CD_3_OD, 400 MHz) of 3-(hydroxyacetyl)-indole (**4**) | | 23 |
| **Figure S25-** ^13^C NMR spectrum (CD_3_OD, 100 MHz) of 3-(hydroxyacetyl)-indole (**4**) | | 24 |
| **Figure S26-** HPLC/UV analyses of brevianamide F (**5**) | | 25 |
| **Figure S27-** HPLC/MS analyses of brevianamide F (**5**) | | 26 |
| **Figure S28-** ^1^H NMR spectrum (CD_3_OD, 400 MHz) of brevianamide F (**5**) | | 27 |
| **Figure S29-** ^13^C NMR spectrum (CD_3_OD, 100 MHz) of brevianamide F (**5**) | | 28 |
| **Figure S30-** HPLC/UV analyses of cyclo-(l-Pro-l-Phe) (**6**) | | 29 |
| **Figure S31-** HPLC/MS analyses of cyclo-(l-Pro-l-Phe) (**6**) | | 30 |
| **Figure S32-** ^1^H NMR spectrum (CD_3_OD, 400 MHz) of cyclo-(l-Pro-l-Phe) (**6**) | | 31 |
| **Figure S33-** ^13^C NMR spectrum (CD_3_OD, 100 MHz) of cyclo-(l-Pro-l-Phe) (**6**) | | 32 |
| **Figure S34-** HPLC/UV analyses of cyclo-(l-Pro-l-Tyr) (**7**) | | 33 |
| **Figure S35-** HPLC/MS analyses of cyclo-(l-Pro-l-Tyr) (**7**) | | 34 |
| **Figure S36-** ^1^H NMR spectrum (CD_3_OD, 400 MHz) of cyclo-(l-Pro-l-Tyr) (**7**) | | 35 |
| **Figure S37-** ^13^C NMR spectrum (CD_3_OD, 100 MHz) of cyclo-(l-Pro-l-Tyr) (**7**) | | 36 |
| **Figure S38-** HPLC/MS analyses of cyclo-(l-Pro-l-Leu) (**8**) | | 37 |
| **Figure S39-** ^1^H NMR spectrum (CD_3_OD, 400 MHz) of cyclo-(l-Pro-l-Leu) (**8**) | | 38 |
| **Figure S40-** ^13^C NMR spectrum (CD_3_OD, 100 MHz) of cyclo-(l-Pro-l-Leu) (**8**) | | 39 |
| **Figure S41-** HPLC/MS analyses of cyclo-(l-Val-l-Phe) (**9**) | | 40 |
| **Figure S42-** ^1^H NMR spectrum (CD_3_OD, 400 MHz) of cyclo-(l-Val-l-Phe) (**9**) | | 41 |
| **Figure S43-** ^13^C NMR spectrum (CD_3_OD, 400 MHz) of cyclo-(l-Val-l-Phe) (**9**) | | 42 |
| **Table S1**- Similarity percentage matrix for the gene 16S rRNA, of LGMB466 and 487- *Actinomadura* sp | | 43 |
| **Table S2**- Similarity percentage matrix for the gene 16S rRNA, of LGMB491-  *Aeromicrobium* sp. | | 49 |
| **Table S3**- Similarity percentage matrix for the gene 16S rRNA, of LGMB471-  *Microbacterium* sp | | 50 |
| **Table S4**- Similarity percentage matrix for the gene 16S rRNA, of LGMB461 and 465- *Microbispora* sp. | | 51 |
| **Table S5**- Similarity percentage matrix for the gene 16S rRNA, of LGMB485- *Micrococcus* sp | | 52 |
| **Table S6**- Similarity percentage matrix for the gene 16S rRNA, of LGMB482- *Sphaerisporangium* sp. | | 53 |
| **Table S7**- Similarity percentage matrix for the gene 16S rRNA, of LGMB483- *Streptomyces* sp | | 52 |
| **Table S8**- Similarity percentage matrix for the gene 16S rRNA, of LGMB479- *Williamsia* sp | | 53 |
| **Table S9**. Dry weight obtained from extracts of endophytic actinomycetes in two media (SG and R5A) and two temperatures (28 °C and 36 °C) | | 56 |

Methicillin-sensitive *Staphylococcus aureus*(MSSA)


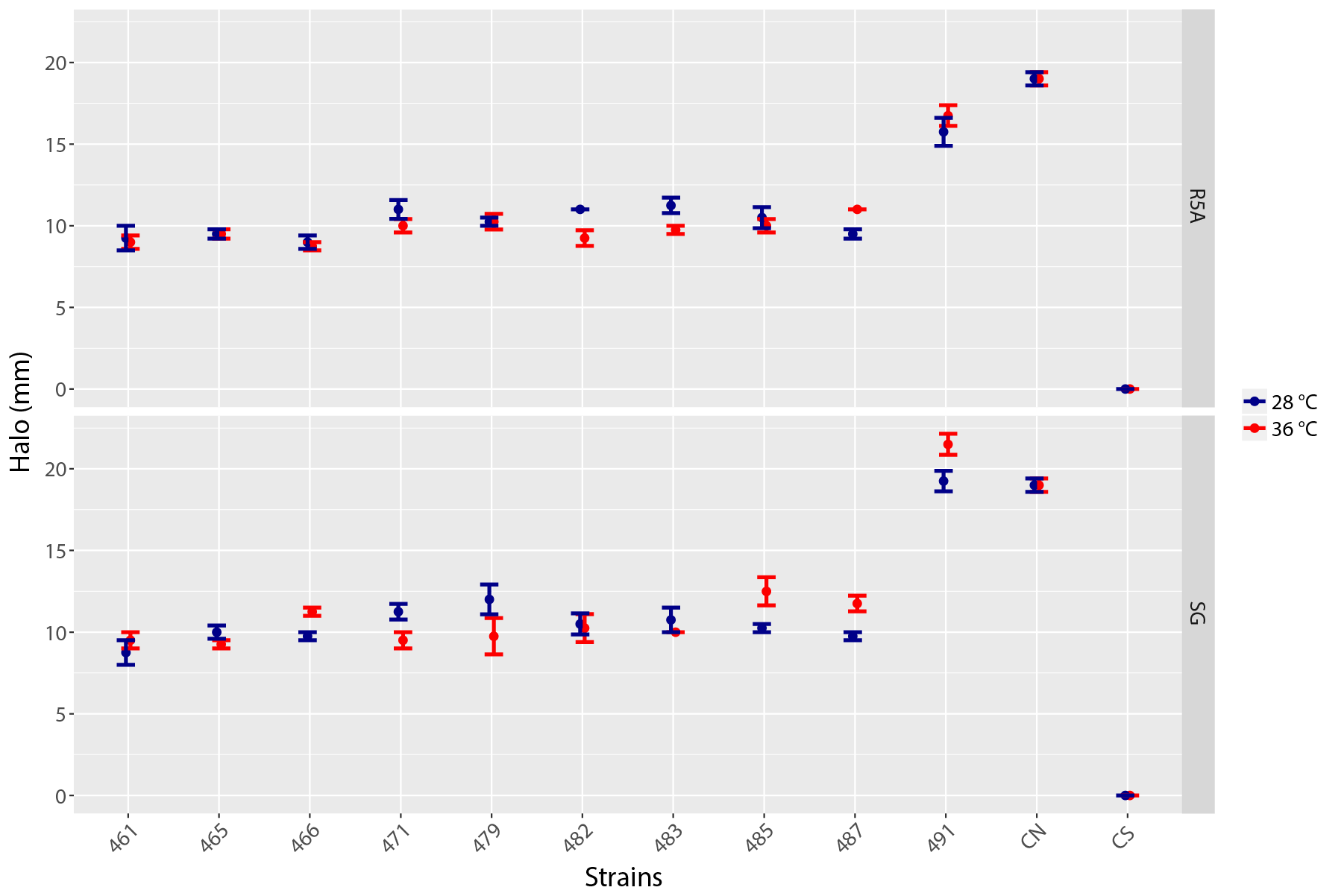


Methicillin-resistant *Staphylococcus aureus* (MRSA)


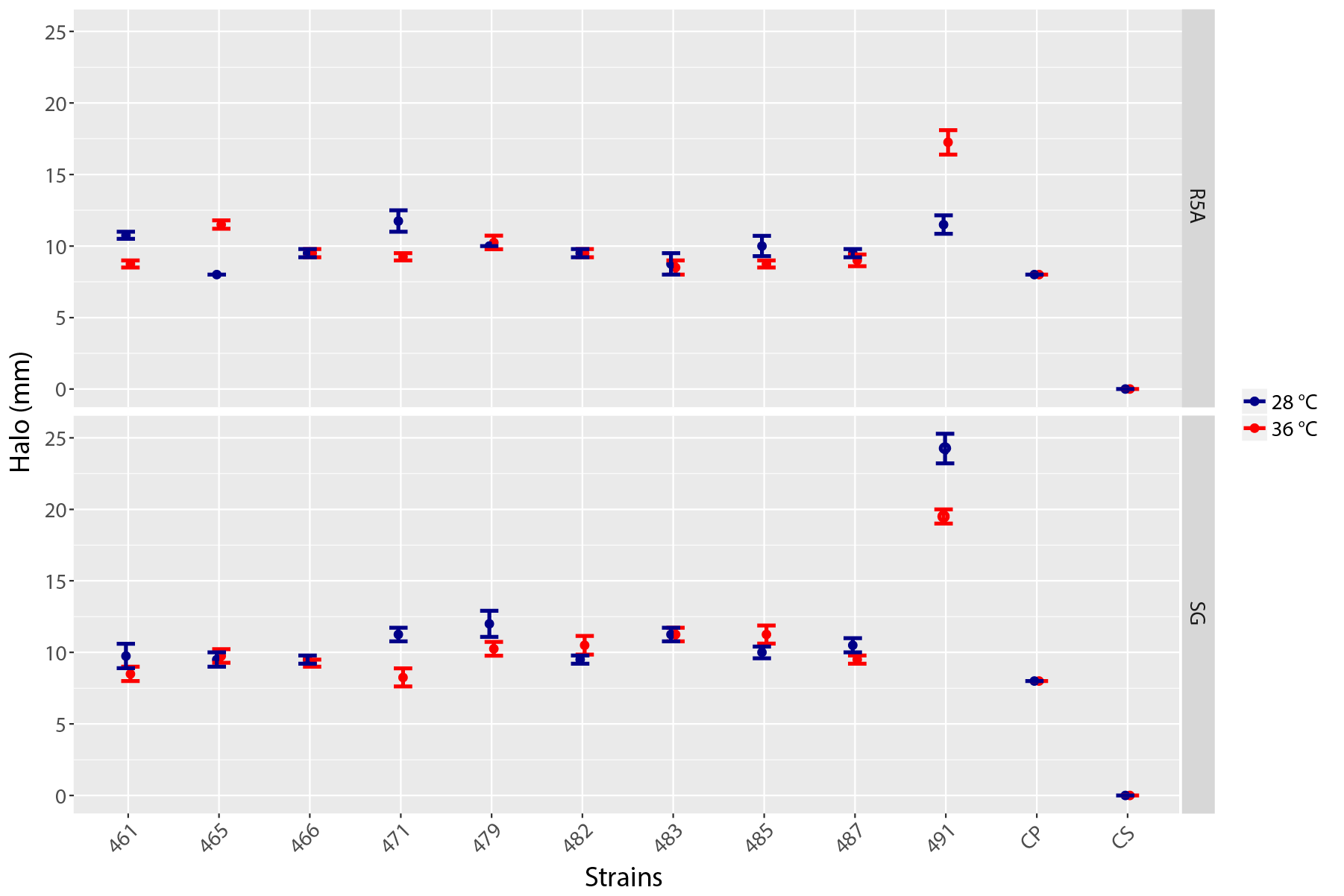


CS

CN

**Figure S1-**Inhibition zone (mm) of Methicillin-resistant *S. aureus (*MRSA) by extracts from endophytic actinomycetes in two media (SG and R5A) at two temperatures (28 °C and 36 °C).

Mean (±SD), CS: Methanol, CN: negative control


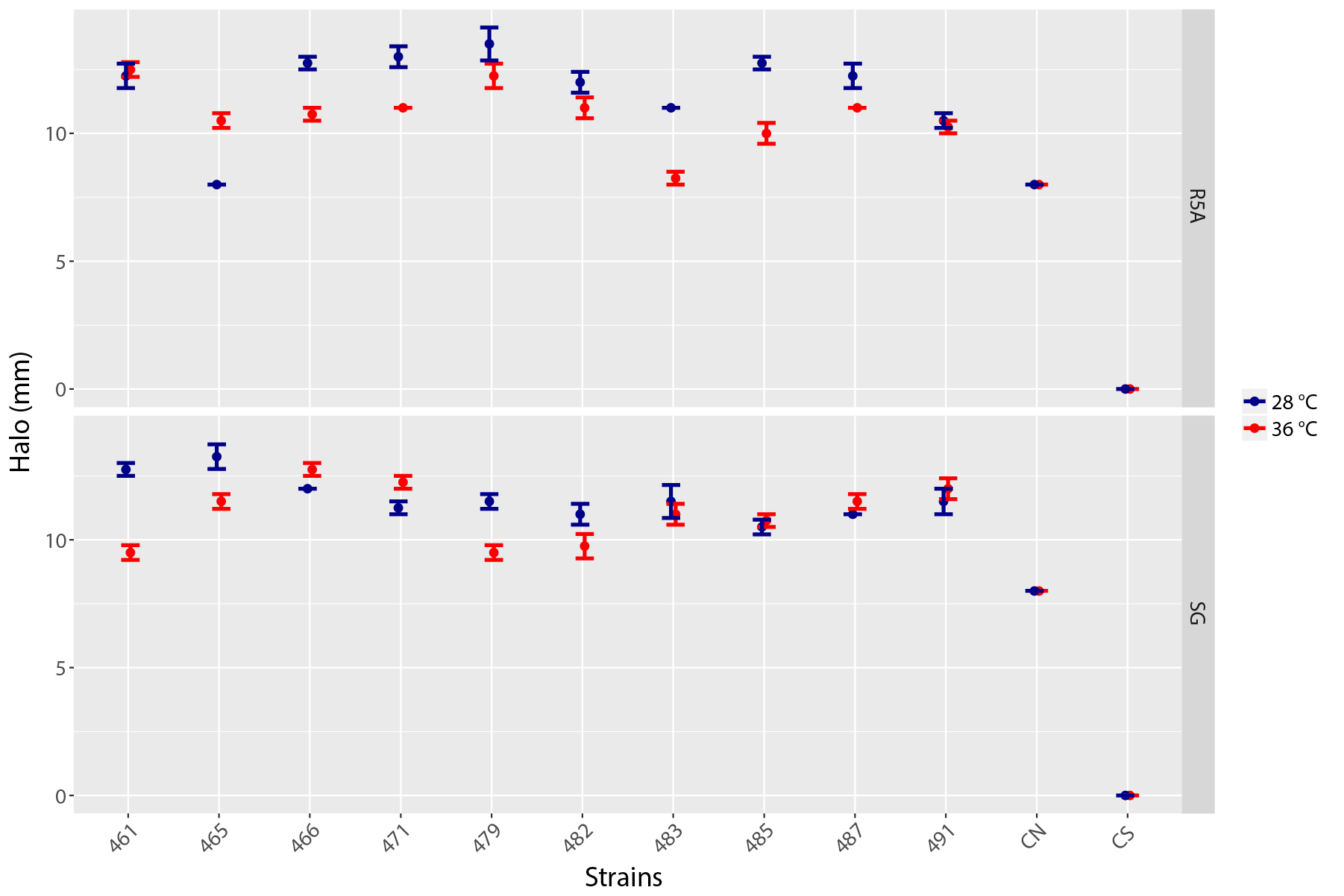


*Escherichia coli*

**Figure S2-**Inhibition zone (mm) of *Escherichia coli* by extracts from endophytic actinomycetes in two media (SG and R5A) at two temperatures (28 °C and 36 °C).

Mean (±SD), CS: Methanol, CN: negative control

*Pseudomonas aeruginosa*


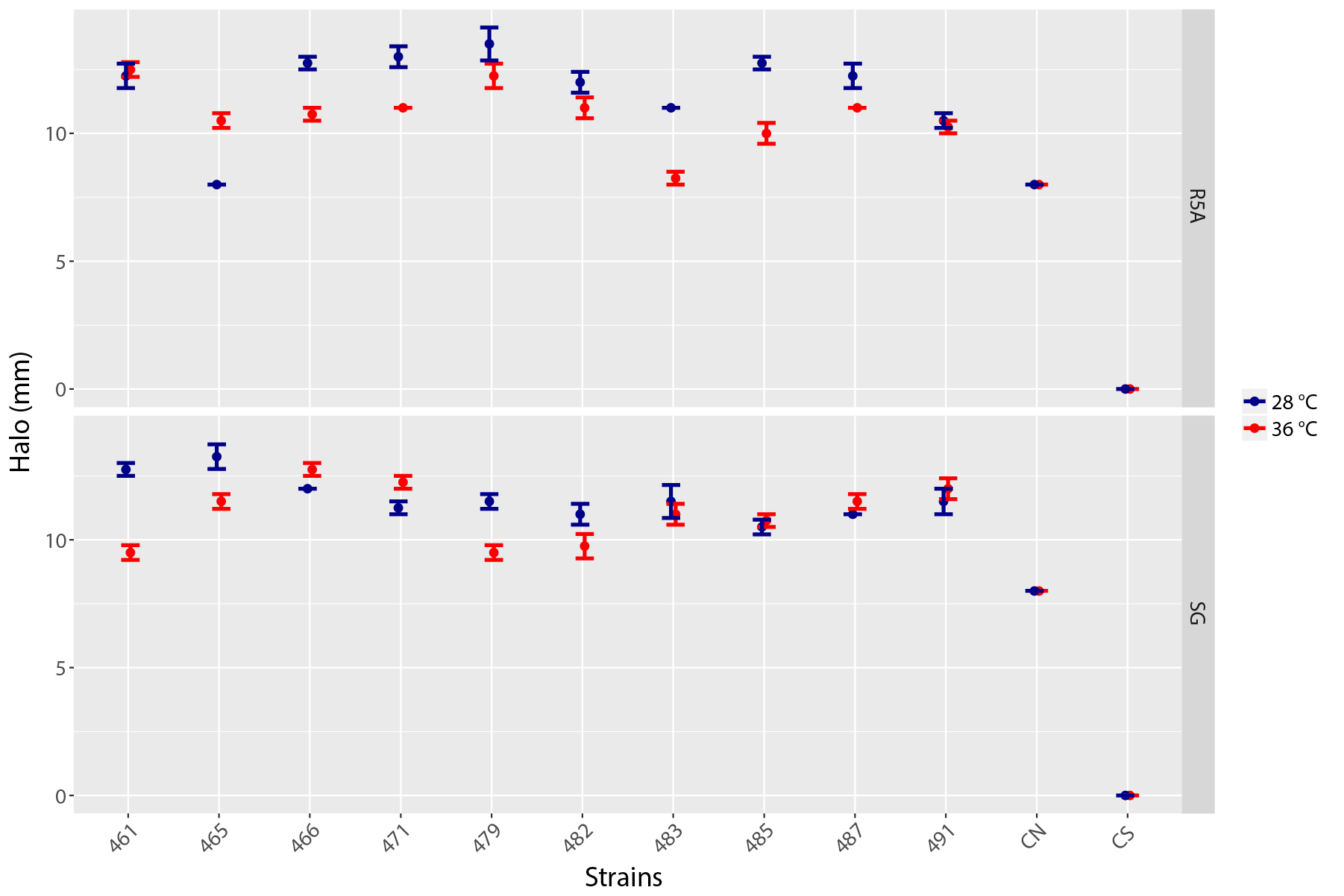


**Figure S3-**Inhibition zone (mm) of *Pseudomonas aeruginosa* by extracts from endophytic actinomycetes in two media (SG and R5A) at two temperatures (28 °C and 36 °C).

Mean (±SD), CS: Methanol, CN: negative control


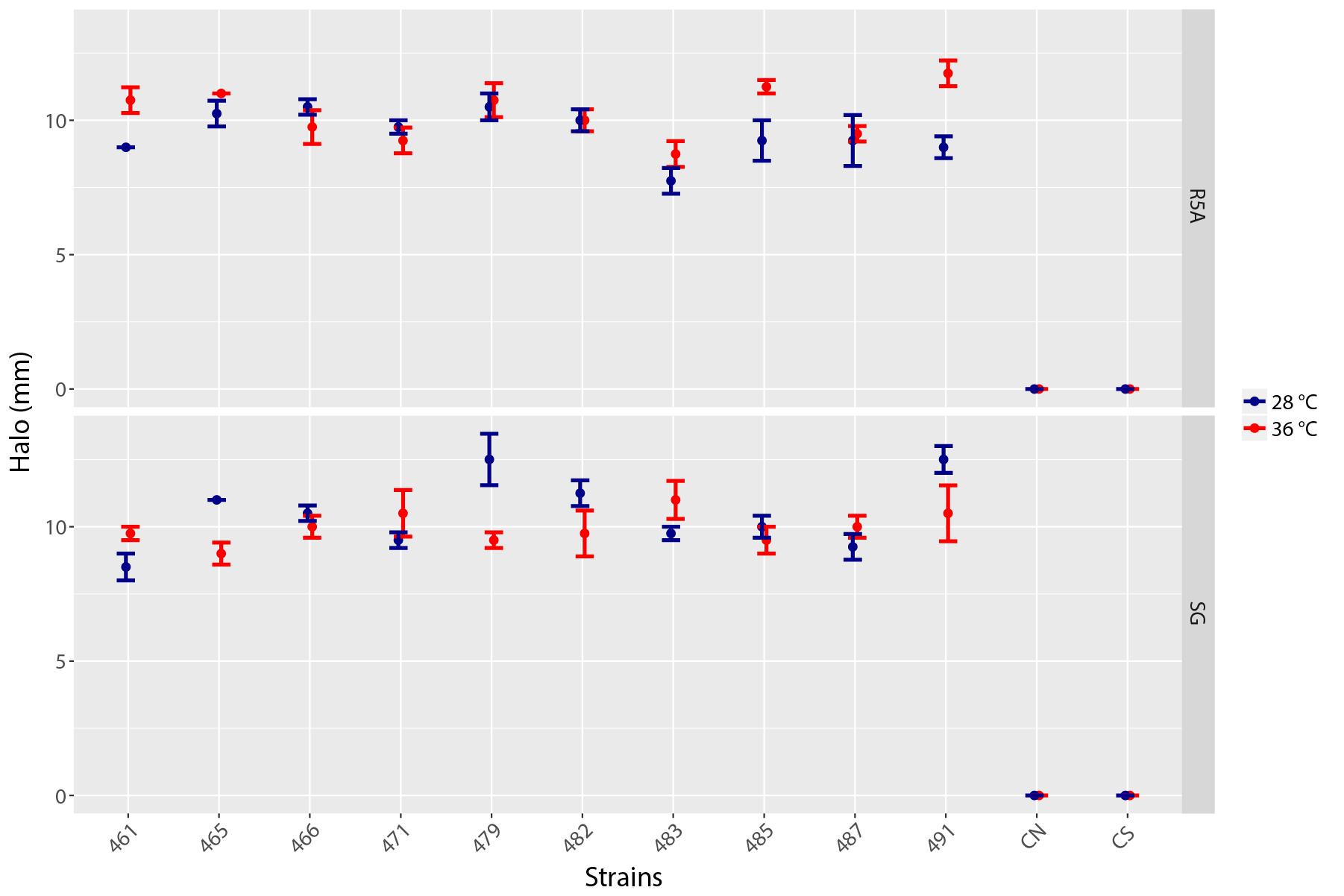


*Acinetobacter baumannii*

**Figure S4-**Inhibition zone (mm) of *Acinetobacter baumannii* by extracts from endophytic actinomycetes in two media (SG and R5A) at two temperatures (28 °C and 36 °C).

Mean (±SD), CS: Methanol, CN: negative control

*Candida albicans*


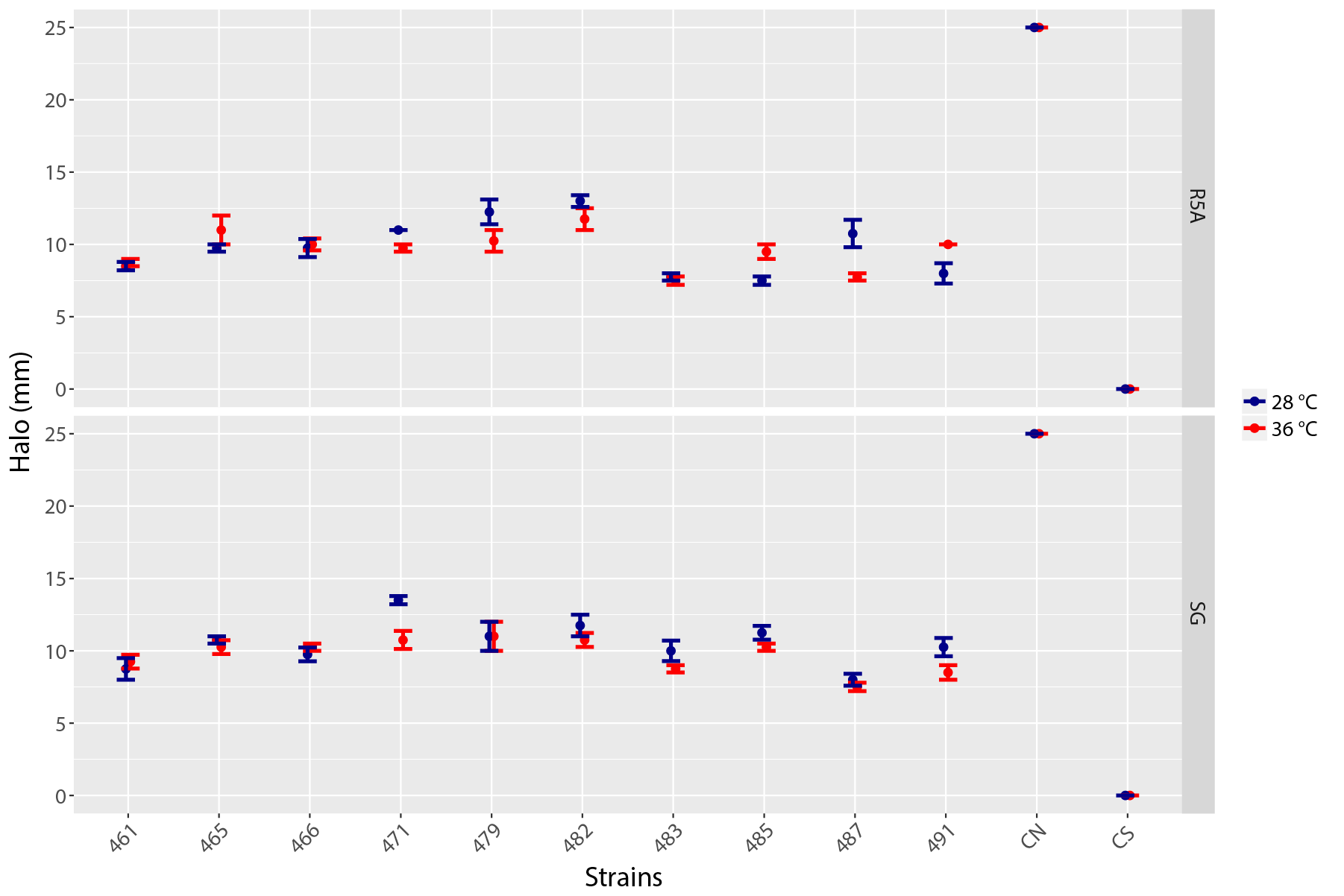


**Figure S5-**Inhibition zone (mm) of *Candida albicans* by extracts from endophytic actinomycetes in two media (SG and R5A) at two temperatures (28 °C and 36 °C).

Mean (±SD), CS: Methanol, CN: negative control


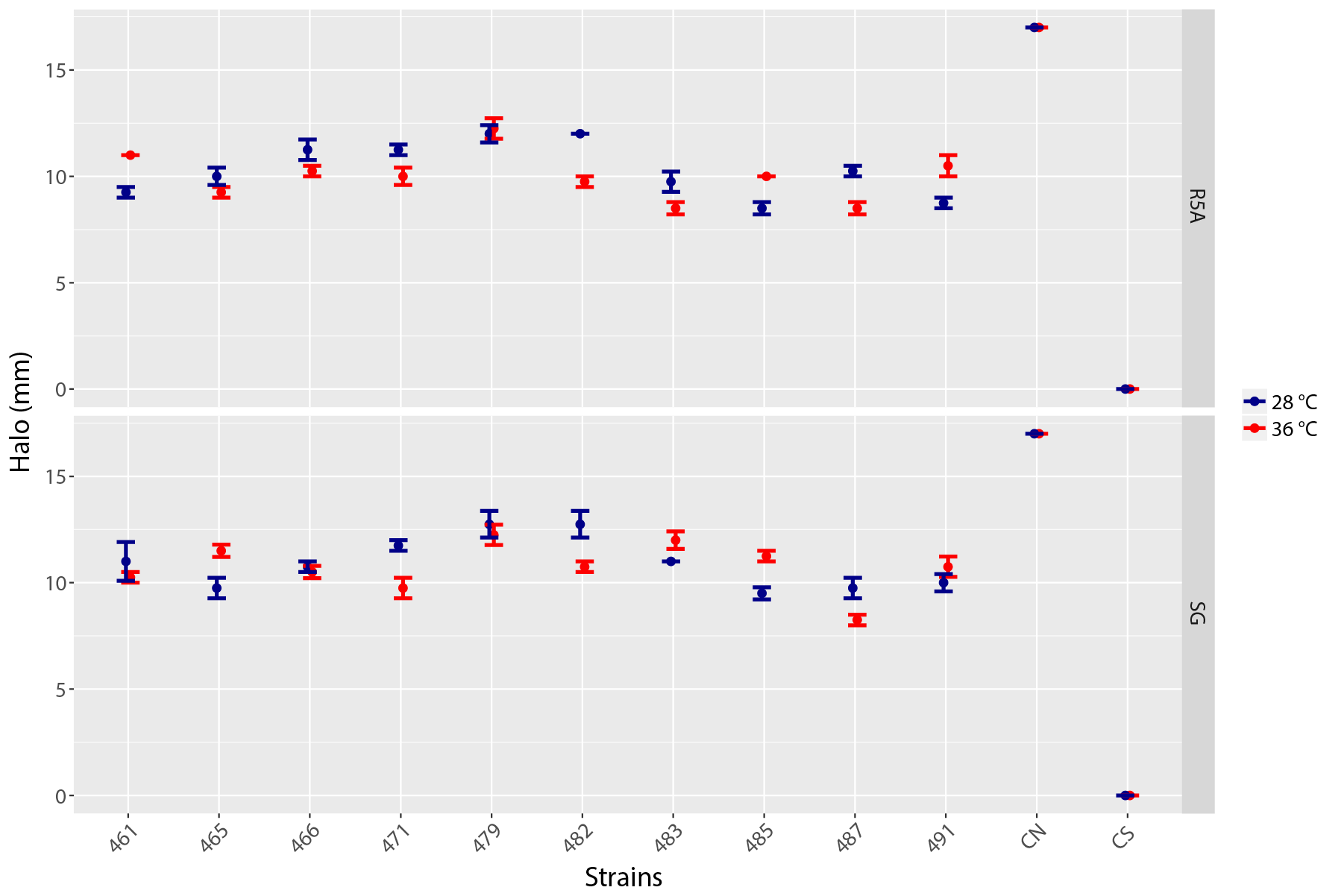


**Figure S6-**Inhibition zone (mm) of *Enterobacter cloacae* producer enzyme VIM (Verona integron-encoded metallo-β-lactamase) by extracts from endophytic actinomycetes in two media (SG and R5A) at two temperatures (28 °C and 36 °C).

*Enterobacter cloacae* produtor da enzima *VIM*

Mean (±SD), CS: Methanol, CN: negative control

*Stenotrophomonas maltophilia*


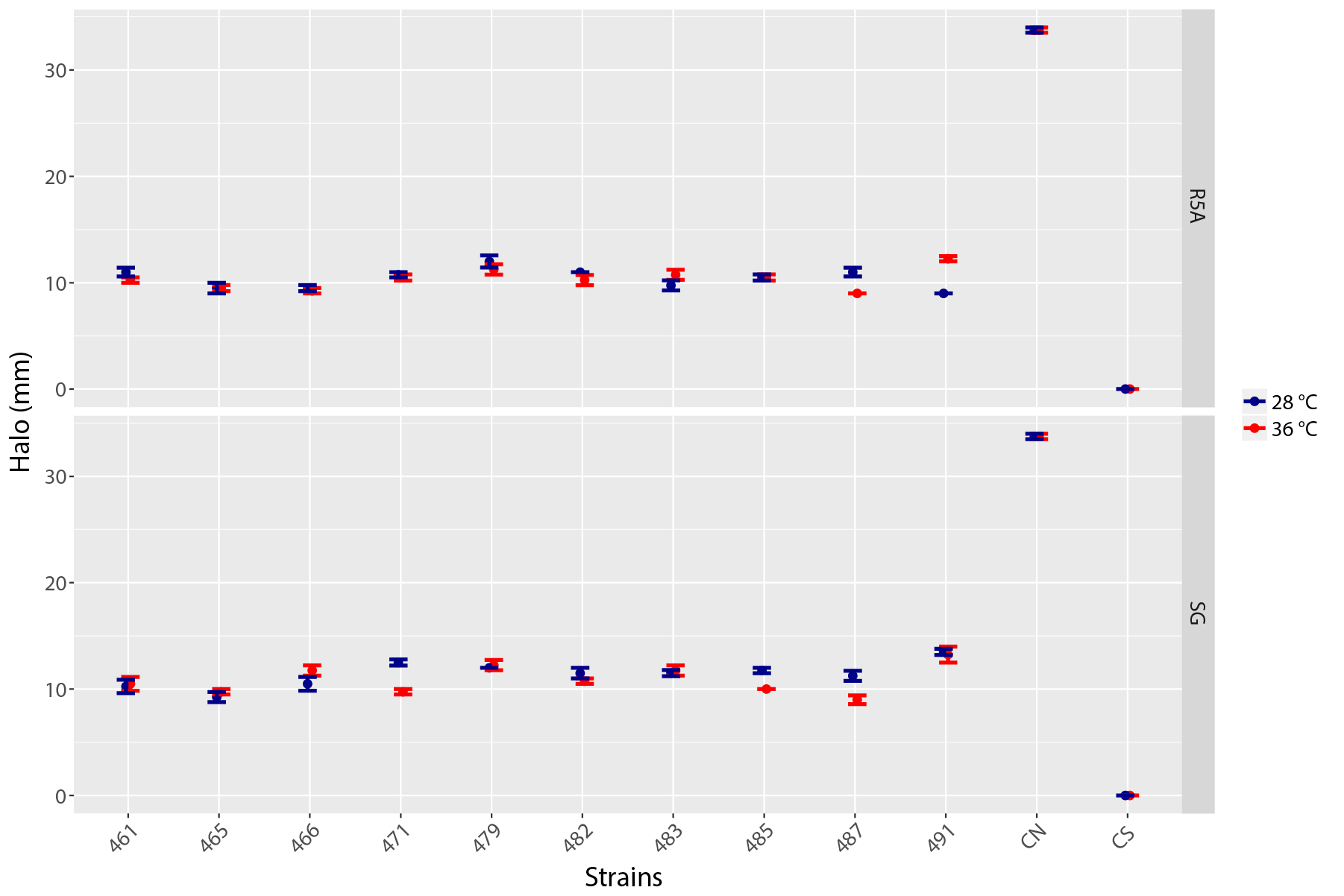


**Figure S7-**Inhibition zone (mm) of *Stenotrophomonas maltophilia* by extracts from endophytic actinomycetes in two media (SG and R5A) and two temperatures (28 °C and 36 °C).

Mean (±SD), CS: Methanol, CN: negative control


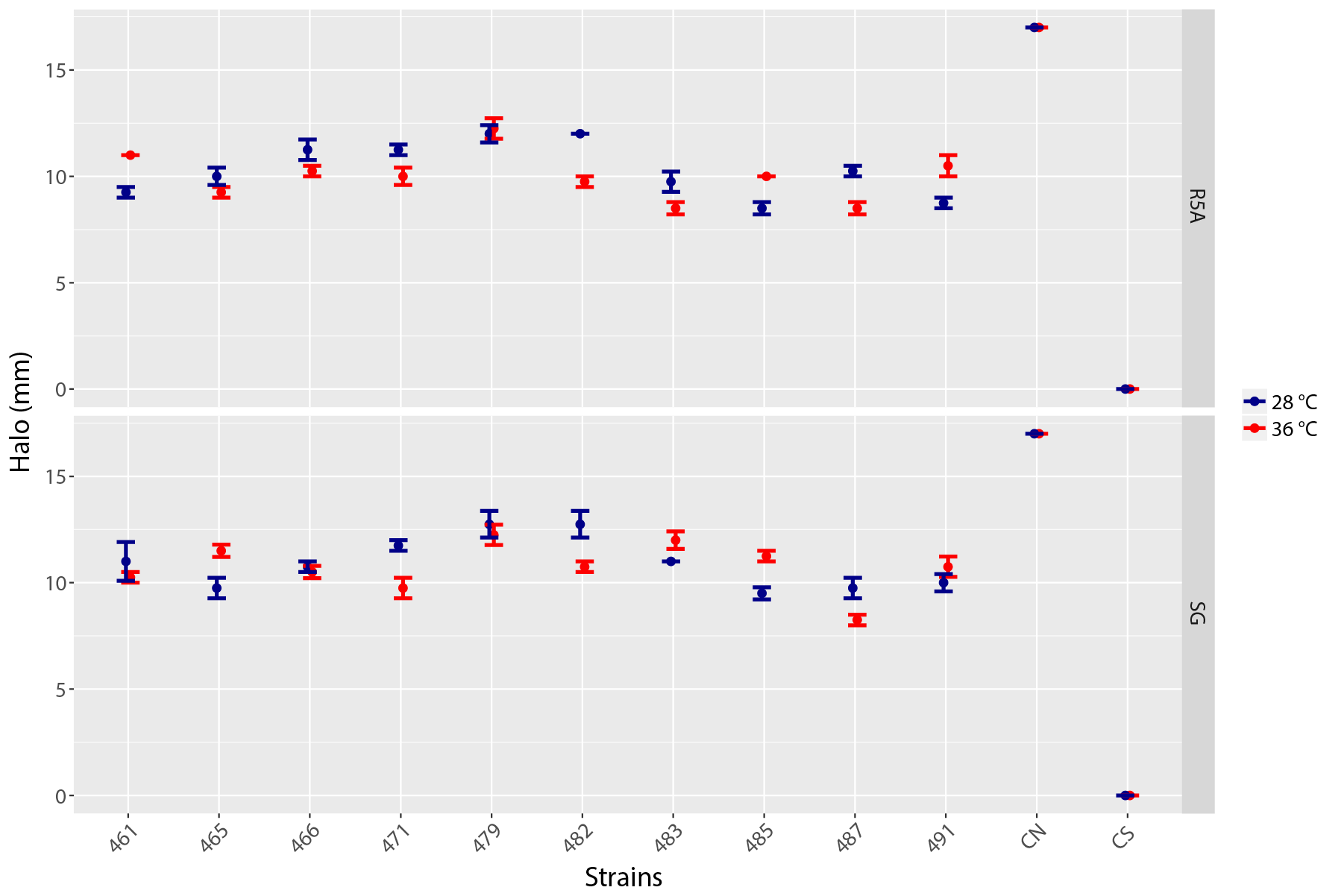


**Figure S8-**Inhibition zone (mm) of *Klebissiella pneumoniae* producer enzyme KPC (*K. pneumoniae* carbapemase) by extracts from endophytic actinomycetes in two media (SG and R5A) at two temperatures (28 °C and 36 °C).

*Klebsiella pneumoniae* producer of enzyme *KPC*

Mean (±SD), CS: Methanol, CN: negative control

**Fig. S9.** Work-up scheme of the metabolites produced by LGMF491


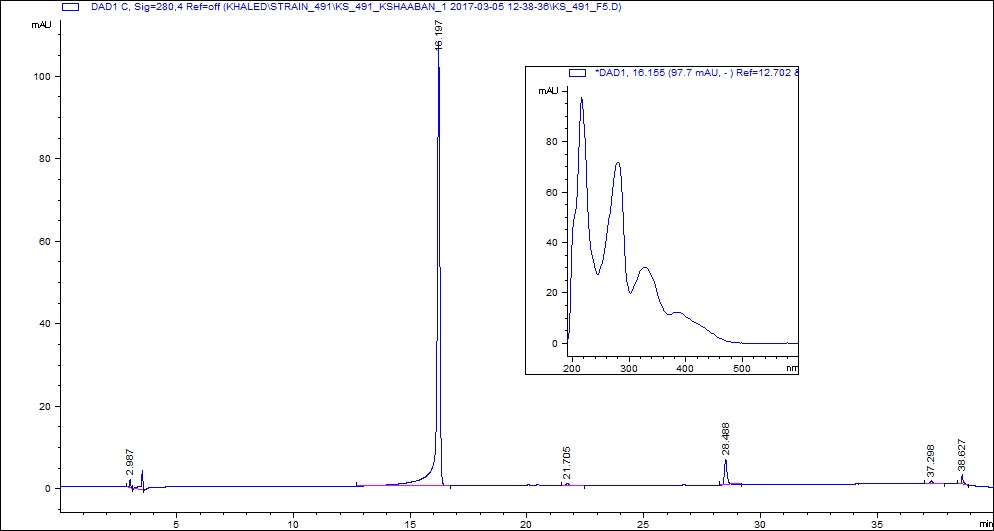

**Figure S10-**HPLC/UV analyses of 1-acetyl-β-carboline (**1**). HPLC-conditions: Detection wavelength 280 nm; solvent A: H_2_O/0.1% TFA; solvent B: acetonitrile; flow rate: 1.0 mL min^-1^; 0-35 min, 95-0% A (linear gradient); 35-40 min 0% A; 40-41 min 0-95% A (linear gradient); 41-45 min 95% A.

**[M + H]^+^**

**Figure S11-**HPLC/MS analyses of 1-acetyl-β-carboline (**1**). HPLC-conditions: Detection wavelength 254 nm; solvent A: H_2_O/0.1% Formic acid, solvent B: CH_3_CN/0.1% Formic acid; flow rate: 0.5 mL min^-1^; 0-4 min, 10% B; 4-22 min, 10-100% B; 22-27 min, 100% B; 27-29 min, 100%-10% B; 29-30 min, 10 % B.

**Figure S12-**^1^H NMR spectrum (DMSO-*d*_6_, 400 MHz) of 1-acetyl-β-carboline (**1**)

**Figure S13-**^13^C NMR spectrum (DMSO-*d*_6_, 100 MHz) of 1-acetyl-β-carboline (**1**)

**
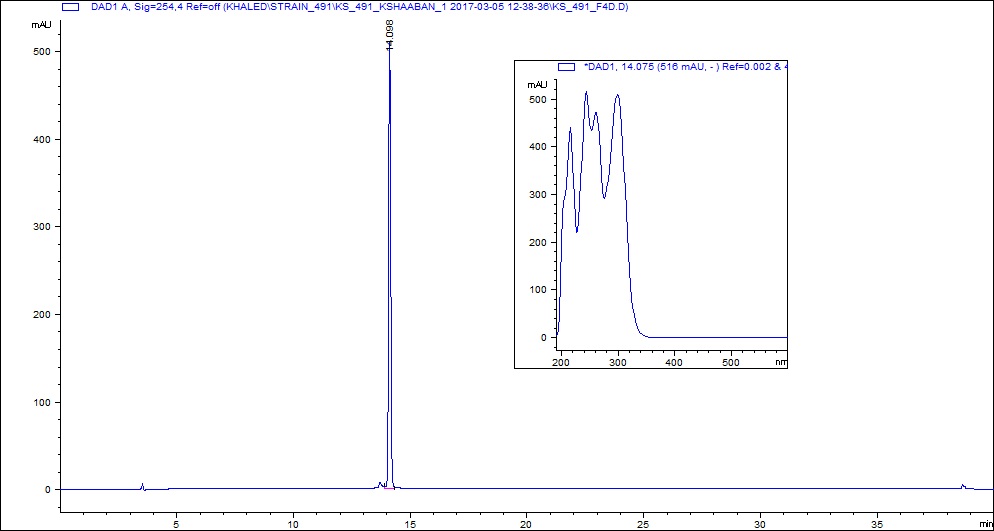
**

**Figure S14-**HPLC/UV analyses of indole-3-carbaldehyde (**2**). HPLC-conditions: Detection wavelength 254 nm; solvent A: H_2_O/0.1% TFA; solvent B: acetonitrile; flow rate: 1.0 mL min^-1^; 0-35 min, 95-0% A (linear gradient); 35-40 min 0% A; 40-41 min 0-95% A (linear gradient); 41-45 min 95% A.

**[M - H]**^−^

**[M + H]^+^**

**Figure S15-**HPLC/MS analyses of indole-3-carbaldehyde (**2**). HPLC-conditions: Detection wavelength 254 nm; solvent A: H_2_O/0.1% Formic acid, solvent B: CH_3_CN/0.1% Formic acid; flow rate: 0.5 mL min^-1^; 0-4 min, 10% B; 4-22 min, 10-100% B; 22-27 min, 100% B; 27-29 min, 100%-10% B; 29-30 min, 10 % B.

**Figure S16-**^1^H NMR spectrum (CD_3_OD, 400 MHz) of indole-3-carbaldehyde (**2**)

**Figure S17-**^13^C NMR spectrum (CD_3_OD, 100 MHz) of indole-3-carbaldehyde (**2**)

**
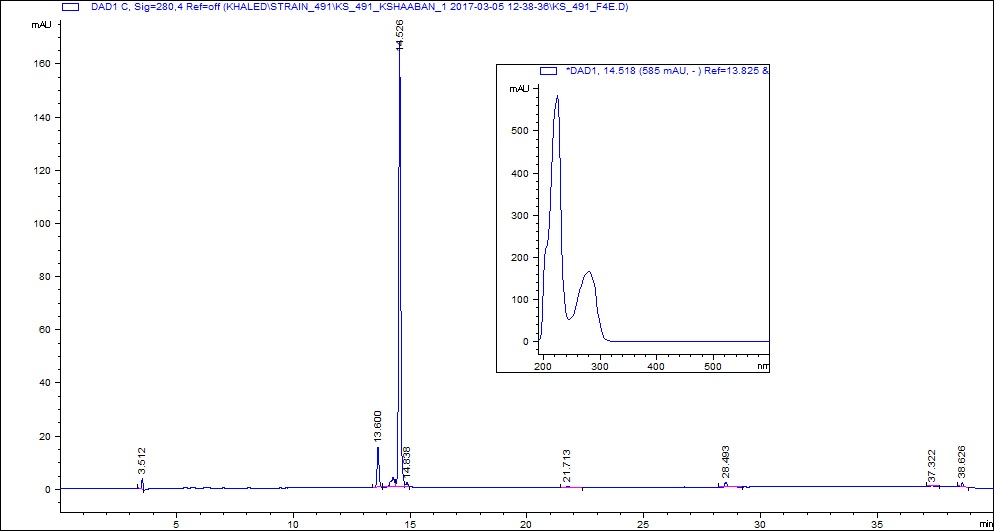
**

**Figure S18-**HPLC/UV analyses of tryptophol (**3**). HPLC-conditions: Detection wavelength 280 nm; solvent A: H_2_O/0.1% TFA; solvent B: acetonitrile; flow rate: 1.0 mL min^-1^; 0-35 min, 95-0% A (linear gradient); 35-40 min 0% A; 40-41 min 0-95% A (linear gradient); 41-45 min 95% A.

**[M + H]^+^**

**Figure S19-**HPLC/MS analyses of tryptophol (**3**). HPLC-conditions: Detection wavelength 254 nm; solvent A: H_2_O/0.1% Formic acid, solvent B: CH_3_CN/0.1% Formic acid; flow rate: 0.5 mL min^-1^; 0-4 min, 10% B; 4-22 min, 10-100% B; 22-27 min, 100% B; 27-29 min, 100%-10% B; 29-30 min, 10 % B.

**Figure S20-**^1^H NMR spectrum (CD_3_OD, 400 MHz) of tryptophol (**3**)

**Figure S21-**^13^C NMR spectrum (CD_3_OD, 100 MHz) of tryptophol (**3**)

**
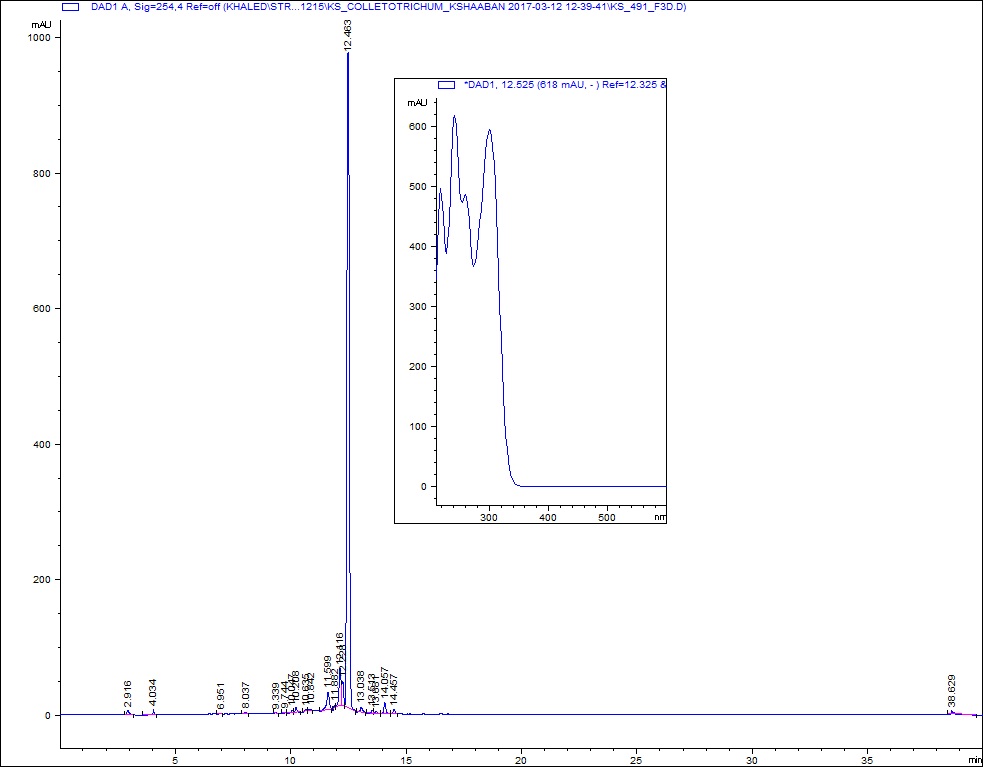
**

**Figure S22-**HPLC/UV analyses of 3-(hydroxyacetyl)-indole (**4**). HPLC-conditions: Detection wavelength 254 nm; solvent A: H_2_O/0.1% TFA; solvent B: acetonitrile; flow rate: 1.0 mL min^-1^; 0-35 min, 95-0% A (linear gradient); 35-40 min 0% A; 40-41 min 0-95% A (linear gradient); 41-45 min 95% A.

**[M - H]**^−^

**[M + H]^+^**

**Figure S23-**HPLC/MS analyses of3-(hydroxyacetyl)-indole (**4**). HPLC-conditions: Detection wavelength 254 nm; solvent A: H_2_O/0.1% Formic acid, solvent B: CH_3_CN/0.1% Formic acid; flow rate: 0.5 mL min^-1^; 0-4 min, 10% B; 4-22 min, 10-100% B; 22-27 min, 100% B; 27-29 min, 100%-10% B; 29-30 min, 10 % B.

**Figure S24-**^1^H NMR spectrum (CD_3_OD, 400 MHz) of 3-(hydroxyacetyl)-indole (**4**)

**Figure S25-**^13^C NMR spectrum (CD_3_OD, 100 MHz) of 3-(hydroxyacetyl)-indole (**4**)


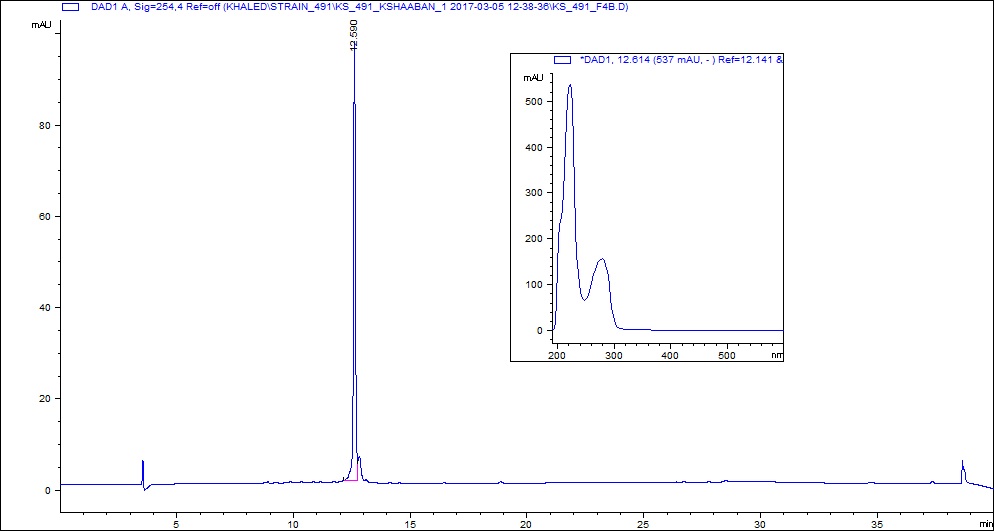

**Figure S26-**HPLC/UV analyses of brevianamide F (**5**). HPLC-conditions: Detection wavelength 254 nm; solvent A: H_2_O/0.1% TFA; solvent B: acetonitrile; flow rate: 1.0 mL min^-1^; 0-35 min, 95-0% A (linear gradient); 35-40 min 0% A; 40-41 min 0-95% A (linear gradient); 41-45 min 95% A.

**[M + H]^+^**

**[M - H]**^−^

**Figure S27-**HPLC/MS analyses ofbrevianamide F (**5**). HPLC-conditions: Detection wavelength 254 nm; solvent A: H_2_O/0.1% Formic acid, solvent B: CH_3_CN/0.1% Formic acid; flow rate: 0.5 mL min^-1^; 0-4 min, 10% B; 4-22 min, 10-100% B; 22-27 min, 100% B; 27-29 min, 100%-10% B; 29-30 min, 10 % B.

**Figure S28-**^1^H NMR spectrum (CD_3_OD, 400 MHz) of brevianamide F (**5**)

**Figure S29-**^13^C NMR spectrum (CD_3_OD, 100 MHz) of brevianamide F (**5**)

**
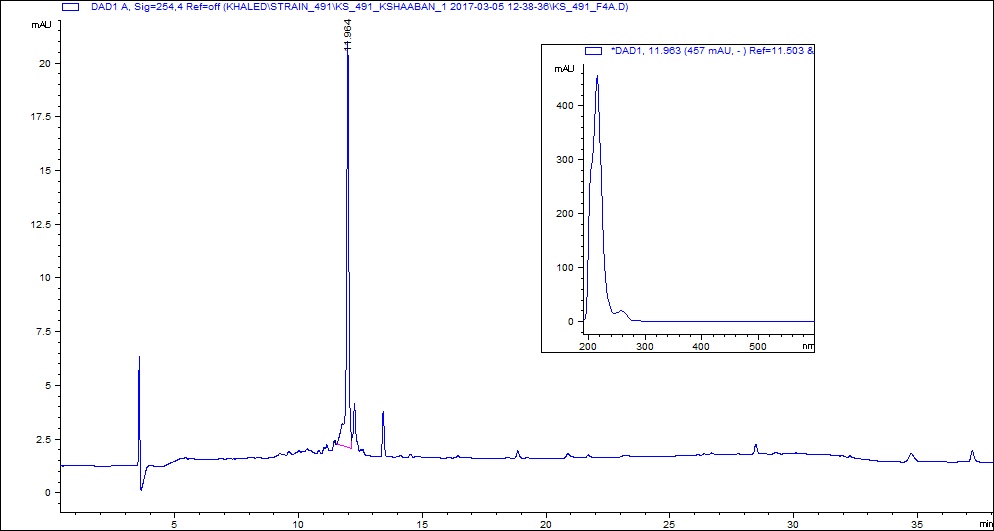
**

**Figure S30-**HPLC/UV analyses of cyclo-(l-Pro-l-Phe) (**6**). HPLC-conditions: Detection wavelength 254 nm; solvent A: H_2_O/0.1% TFA; solvent B: acetonitrile; flow rate: 1.0 mL min^-1^; 0-35 min, 95-0% A (linear gradient); 35-40 min 0% A; 40-41 min 0-95% A (linear gradient); 41-45 min 95% A.

**[M + H]^+^**

**Figure S31-**HPLC/MS analyses ofcyclo-(l-Pro-l-Phe) (**6**). HPLC-conditions: Detection wavelength 254 nm; solvent A: H_2_O/0.1% Formic acid, solvent B: CH_3_CN/0.1% Formic acid; flow rate: 0.5 mL min^-1^; 0-4 min, 10% B; 4-22 min, 10-100% B; 22-27 min, 100% B; 27-29 min, 100%-10% B; 29-30 min, 10 % B.

**Figure S32-**^1^H NMR spectrum (CD_3_OD, 400 MHz) of cyclo-(l-Pro-l-Phe) (**6**)

**Figure S33-**^13^C NMR spectrum (CD_3_OD, 100 MHz) of cyclo-(l-Pro-l-Phe) (**6**)

**
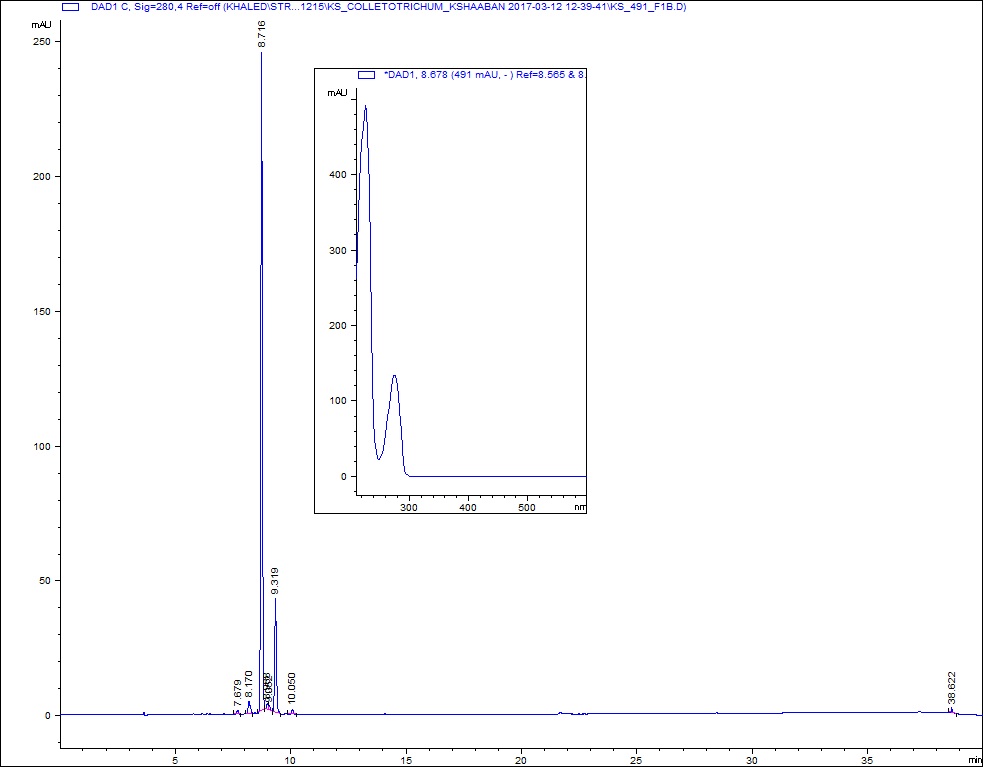
**

**Figure S34-**HPLC/UV analyses of cyclo-(l-Pro-l-Tyr) (**7**). HPLC-conditions: Detection wavelength 254 nm; solvent A: H_2_O/0.1% TFA; solvent B: acetonitrile; flow rate: 1.0 mL min^-1^; 0-35 min, 95-0% A (linear gradient); 35-40 min 0% A; 40-41 min 0-95% A (linear gradient); 41-45 min 95% A.

**[M - H]**^−^

**[M + H]^+^**

**Figure S35-**HPLC/MS analyses ofcyclo-(l-Pro-l-Tyr) (**7**). HPLC-conditions: Detection wavelength 254 nm; solvent A: H_2_O/0.1% Formic acid, solvent B: CH_3_CN/0.1% Formic acid; flow rate: 0.5 mL min^-1^; 0-4 min, 10% B; 4-22 min, 10-100% B; 22-27 min, 100% B; 27-29 min, 100%-10% B; 29-30 min, 10 % B.

**Figure S36-**^1^H NMR spectrum (CD_3_OD, 400 MHz) of cyclo-(l-Pro-l-Tyr) (**7**)

**Figure S37-**^13^C NMR spectrum (CD_3_OD, 100 MHz) of cyclo-(l-Pro-l-Tyr) (**7**)

**[M + H]^+^**

**Figure S38-**HPLC/MS analyses of cyclo-(l-Pro-l-Leu) (**8**). HPLC-conditions: Detection wavelength 254 nm; solvent A: H_2_O/0.1% Formic acid, solvent B: CH_3_CN/0.1% Formic acid; flow rate: 0.5 mL min^-1^; 0-4 min, 10% B; 4-22 min, 10-100% B; 22-27 min, 100% B; 27-29 min, 100%-10% B; 29-30 min, 10 % B.

**Figure S39-**^1^H NMR spectrum (CD_3_OD, 400 MHz) of cyclo-(l-Pro-l-Leu) (**8**)

**Figure S40-**^13^C NMR spectrum (CD_3_OD, 100 MHz) of cyclo-(l-Pro-l-Leu) (**8**)

**[M + H]^+^**

**Figure S41-**HPLC/MS analyses of cyclo-(l-Val-l-Phe) (**9**). HPLC-conditions: Detection wavelength 254 nm; solvent A: H_2_O/0.1% Formic acid, solvent B: CH_3_CN/0.1% Formic acid; flow rate: 0.5 mL min^-1^; 0-4 min, 10% B; 4-22 min, 10-100% B; 22-27 min, 100% B; 27-29 min, 100%-10% B; 29-30 min, 10 % B.

**Figure S42-**^1^H NMR spectrum (CD_3_OD, 400 MHz) of cyclo-(l-Val-l-Phe) (**9**)

**Figure S43-**^13^C NMR spectrum (CD_3_OD, 100 MHz) of cyclo-(l-Val-l-Phe) (**9**)

Table S1- Similarity percentage matrix for the gene 16S rRNA, of LGMB466 and 487- *Actinomadura* genus

|  |  | **[1]** | **[2]** | **[3]** | **[4]** | **[5]** | **[6]** | **[7]** | **[8]** | **[9]** | **[10]** | **[11]** | **[12]** | **[13]** | **[14]** |
| --- | --- | --- | --- | --- | --- | --- | --- | --- | --- | --- | --- | --- | --- | --- | --- |
| **[1]** | *Actinomadura alba* |  |  |  |  |  |  |  |  |  |  |  |  |  |  |
| **[2]** | *Actinomadura amylolytica* | 94,72 |  |  |  |  |  |  |  |  |  |  |  |  |  |
| **[3]** | *Actinomadura apis* | 93,79 | 93,94 |  |  |  |  |  |  |  |  |  |  |  |  |
| **[4]** | *Actinomadura atramentaria* | 93,01 | 94,29 | 94,79 |  |  |  |  |  |  |  |  |  |  |  |
| **[5]** | *Actinomadura bangladeshensis* | 94,29 | 95,65 | 97 | 95,79 |  |  |  |  |  |  |  |  |  |  |
| **[6]** | *Actinomadura cellulosilytica* | 94,58 | 98,07 | 94,08 | 94,29 | 95,44 |  |  |  |  |  |  |  |  |  |
| **[7]** | *Actinomadura chokoriensis* | 93,3 | 94,86 | 96,08 | 94,72 | 98,64 | 94,44 |  |  |  |  |  |  |  |  |
| **[8]** | *Actinomadura citrea* | 94,15 | 94,79 | 97,15 | 95,86 | 98,07 | 94,94 | 97 |  |  |  |  |  |  |  |
| **[9]** | *Actinomadura coerulea* | 93,58 | 94,51 | 96,72 | 96,01 | 97,65 | 94,58 | 96,58 | 98,79 |  |  |  |  |  |  |
| **[10]** | *Actinomadura cremea* | 92,94 | 94,01 | 98,72 | 94,72 | 96,93 | 94,22 | 96,08 | 97,22 | 96,79 |  |  |  |  |  |
| **[11]** | *Actinomadura darangshiensis* | 94,08 | 95,22 | 96,43 | 95,29 | 98,22 | 95,44 | 97,15 | 97,57 | 96,86 | 96,36 |  |  |  |  |
| **[12]** | *Actinomadura echinospora* | 95,44 | 97,43 | 94,94 | 94,22 | 95,58 | 97,22 | 94,58 | 95,36 | 94,86 | 94,08 | 95,22 |  |  |  |
| **[13]** | *Actinomadura fibrosa* | 94,94 | 95,08 | 96,93 | 95,86 | 97,08 | 94,94 | 96,01 | 97,43 | 97,29 | 96,01 | 96,15 | 95,72 |  |  |
| **[14]** | *Actinomadura flavalba* | 93,72 | 95,15 | 95,29 | 97,08 | 96,29 | 95,51 | 95,22 | 96,29 | 95,65 | 95,22 | 96,36 | 95,01 | 96,22 |  |
| **[15]** | *Actinomadura formosensis* | 93,51 | 94,51 | 96,86 | 95,44 | 97,43 | 94,51 | 96,5 | 97,93 | 97,36 | 96,93 | 97 | 94,65 | 96,65 | 95,44 |
| **[16]** | *Actinomadura geliboluensis* | 94,01 | 95,15 | 97,43 | 96,08 | 98,64 | 95,29 | 97,72 | 98 | 97,57 | 97,36 | 97,86 | 95,29 | 96,86 | 96,29 |
| **[17]** | *Actinomadura glauciflava* | 94,15 | 95,08 | 97,43 | 95,86 | 98,22 | 95,15 | 97,15 | 99,5 | 99,14 | 97,5 | 97,43 | 95,44 | 97,72 | 96,36 |
| **[18]** | *Actinomadura hallensis* | 94,58 | 93,79 | 94,94 | 94,22 | 95,93 | 94,15 | 94,94 | 95,22 | 94,79 | 94,94 | 95,22 | 94,08 | 94,44 | 94,72 |
| **[19]** | *Actinomadura keratinilytica* | 94,29 | 94,15 | 95,58 | 96,08 | 95,51 | 94,58 | 94,51 | 95,08 | 94,65 | 94,65 | 95,01 | 95,15 | 95,93 | 96,29 |
| **[20]** | *Actinomadura kijaniata* | 94,01 | 94,79 | 96,01 | 95,51 | 96,5 | 94,72 | 95,44 | 95,86 | 95,51 | 95,08 | 96,01 | 94,94 | 96,58 | 96,65 |
| **[21]** | *Actinomadura latina* | 93,94 | 94,72 | 96,43 | 95,08 | 97,72 | 94,86 | 96,79 | 96,93 | 96,22 | 96,22 | 97,57 | 94,65 | 96,08 | 95,86 |
| **[22]** | *Actinomadura luteofluorescens* | 93,58 | 94,51 | 96,65 | 95,72 | 97,65 | 94,51 | 96,58 | 98,64 | 99,14 | 96,72 | 96,79 | 94,86 | 97 | 95,72 |
| **[23]** | *Actinomadura macra* | 93,72 | 94,58 | 96,15 | 95,51 | 97,36 | 94,29 | 96,43 | 97,79 | 98,07 | 96,08 | 97 | 94,94 | 96,22 | 95,15 |
| **[24]** | *Actinomadura madurae* | 94,58 | 95,36 | 97,15 | 95,44 | 98,22 | 95,22 | 97,15 | 98,22 | 97,36 | 97 | 98,57 | 95,51 | 96,79 | 96,36 |

Table S1- Similarity percentage matrix for the gene 16S rRNA, of LGMB466 and 487- *Actinomadura* genus

|  | |  | | **[1]** | | **[2]** | | **[3]** | | **[4]** | | **[5]** | | **[6]** | | **[7]** | | **[8]** | | **[9]** | | **[10]** | | **[11]** | | **[12]** | | **[13]** | | **[14]** | |
| --- | --- | --- | --- | --- | --- | --- | --- | --- | --- | --- | --- | --- | --- | --- | --- | --- | --- | --- | --- | --- | --- | --- | --- | --- | --- | --- | --- | --- | --- | --- | --- |
| **[25]** | | *Actinomadura mexicana* | | 94,22 | | 94,94 | | 97,08 | | 95,72 | | 98 | | 95,01 | | 96,93 | | 99,36 | | 98,57 | | 97,08 | | 97,43 | | 95,08 | | 97,36 | | 96,36 | |
| **[26]** | | *Actinomadura namibiensis* | | 93,94 | | 94,72 | | 96,08 | | 95,36 | | 96,29 | | 94,79 | | 95,29 | | 95,72 | | 95,29 | | 95,22 | | 95,79 | | 95,01 | | 96,36 | | 96,5 | |
| **[27]** | | *Actinomadura meyerae* | | 93,94 | | 95,22 | | 97 | | 95,86 | | 98,93 | | 95,22 | | 97,86 | | 98,07 | | 97,86 | | 96,86 | | 97,79 | | 95,36 | | 96,79 | | 96,43 | |
| **[28]** | | *Actinomadura nitritigenes* | | 95,01 | | 95,29 | | 96,72 | | 95,86 | | 97,57 | | 95,51 | | 96,5 | | 97,22 | | 97 | | 95,79 | | 96,93 | | 96,22 | | 98,22 | | 96,65 | |
| **[29]** | | *Actinomadura miaoliensis* | | 93,94 | | 94,44 | | 95,44 | | 96,01 | | 96,01 | | 94,58 | | 95,01 | | 95,36 | | 94,94 | | 94,58 | | 95,36 | | 95,44 | | 96,29 | | 96,36 | |
| **[30]** | | *Actinomadura rifamycini* | | 92,65 | | 92,87 | | 98,43 | | 94,29 | | 95,79 | | 93,08 | | 94,72 | | 95,93 | | 96,36 | | 97,79 | | 95,44 | | 93,79 | | 95,86 | | 94,37 | |
| **[31]** | | *Actinomadura rayongensis* | | 92,94 | | 94,79 | | 95,08 | | 97,08 | | 96,08 | | 94,79 | | 94,94 | | 96,22 | | 95,79 | | 95,08 | | 95,01 | | 94,72 | | 96,08 | | 96,72 | |
| **[32]** | | *Actinomadura napierensis* | | 94,44 | | 95,15 | | 96,93 | | 95,58 | | 98,22 | | 95,01 | | 97,22 | | 97,57 | | 97 | | 96,86 | | 97,43 | | 95,01 | | 96,93 | | 96,58 | |
| **[33]** | | *Actinomadura rugatobispora* | | 92,87 | | 94,08 | | 94,79 | | 94,94 | | 96,58 | | 93,65 | | 95,79 | | 95,93 | | 96,29 | | 94,86 | | 96,08 | | 93,94 | | 95,29 | | 95,15 | |
| **[34]** | | *Actinomadura rubrobrunea* | | 93,22 | | 93,72 | | 94,65 | | 94,58 | | 94,65 | | 93,51 | | 93,79 | | 94,01 | | 93,51 | | 94,08 | | 94,22 | | 94,37 | | 94,58 | | 95,29 | |
| **[35]** | | *Actinomadura scrupuli* | | 95,22 | | 94,86 | | 94,65 | | 93,3 | | 95,15 | | 95,01 | | 94,15 | | 94,51 | | 94,44 | | 93,79 | | 95,01 | | 95,93 | | 95,51 | | 94,44 | |
| **[36]** | | *Actinomadura umbrina* | | 94,44 | | 97 | | 93,94 | | 93,79 | | 95,22 | | 97,5 | | 94,37 | | 94,65 | | 94,37 | | 94,08 | | 95,44 | | 96,65 | | 94,72 | | 95,15 | |
| **[37]** | | *Actinomadura sputi* | | 94,51 | | 94,44 | | 95,22 | | 94,44 | | 96,5 | | 94,22 | | 95,51 | | 95,44 | | 94,94 | | 95,22 | | 95,86 | | 94,37 | | 94,44 | | 95,22 | |
| **[38]** | | *Actinomadura viridilutea* | | 93,15 | | 93,65 | | 94,58 | | 94,51 | | 94,58 | | 93,44 | | 93,72 | | 93,94 | | 93,44 | | 93,87 | | 94,15 | | 94,29 | | 94,51 | | 95,22 | |
| **[39]** | | *Actinomadura sediminis* | | 92,44 | | 93,94 | | 97,15 | | 94,44 | | 96,79 | | 93,65 | | 95,86 | | 96,29 | | 95,86 | | 97,29 | | 96,08 | | 93,94 | | 95,36 | | 94,72 | |
| **[40]** | | *Actinomadura yumaensis* | | 93,79 | | 94,72 | | 95,44 | | 94,37 | | 96,79 | | 94,72 | | 96,01 | | 96,65 | | 96,29 | | 95,58 | | 96,08 | | 94,72 | | 95,72 | | 95,44 | |
| **[41]** | | *Actinomadura vinacea* | | 93,65 | | 95,08 | | 95,22 | | 95,01 | | 97 | | 94,65 | | 96,15 | | 96,65 | | 96,15 | | 95,29 | | 96,43 | | 95,01 | | 95,65 | | 95,22 | |
| **[42]** | | *Actinomadura xylanilytica* | | 94,86 | | 95,65 | | 96,22 | | 96,43 | | 97,86 | | 95,86 | | 96,79 | | 97,79 | | 97,36 | | 96,29 | | 97,08 | | 95,79 | | 98 | | 97,15 | |
| **[43]** | | *Actinomadura livida* | | 94,44 | | 94,86 | | 95,29 | | 94,51 | | 96,58 | | 95,01 | | 95,65 | | 96,22 | | 95,79 | | 95,22 | | 95,86 | | 94,86 | | 95,58 | | 95,15 | |
| **[44]** | | *Actinomadura hibisca* | | 93,37 | | 93,65 | | 96,15 | | 95,58 | | 96,22 | | 94,01 | | 95,22 | | 96,15 | | 95,79 | | 95,29 | | 95,72 | | 94,58 | | 96,93 | | 96,5 | |
| **[45]** | | *Actinomadura meridiana* | | 93,15 | | 94,29 | | 95,72 | | 94,44 | | 96,93 | | 94,44 | | 95,93 | | 97,08 | | 96,29 | | 95,79 | | 96,93 | | 94,51 | | 95,79 | | 94,86 | |
| **[46]** | | *Actinomadura fulvescens* | | 94,29 | | 94,72 | | 96,65 | | 95,22 | | 97,86 | | 94,65 | | 96,86 | | 97,36 | | 96,72 | | 95,79 | | 96,93 | | 95,86 | | 97,36 | | 95,93 | |
| **[47]** | | *Actinomadura chibensis* | | 93,3 | | 94,79 | | 95,22 | | 93,94 | | 96,72 | | 95,01 | | 95,86 | | 96,01 | | 95,58 | | 95,15 | | 96,01 | | 95,08 | | 95,08 | | 94,86 | |
| **[48]** | | *Actinomadura catellatispora* | | 92,37 | | 92,87 | | 93,22 | | 92,58 | | 94,65 | | 93,01 | | 93,65 | | 94,15 | | 93,72 | | 93,15 | | 93,79 | | 92,87 | | 93,51 | | 93,08 | |
| **[49]** | | *Actinomadura pelletieri* | | 93,08 | | 93,79 | | 95,58 | | 93,87 | | 96,08 | | 94,01 | | 95,15 | | 95,86 | | 95,36 | | 95,65 | | 95,65 | | 94,29 | | 94,79 | | 94,08 | |
| **[50]** | | *Actinomadura oligospora* | | 93,72 | | 94,22 | | 95,72 | | 95,36 | | 96,01 | | 94,08 | | 95,08 | | 95,65 | | 95,01 | | 94,65 | | 95,08 | | 95,08 | | 95,72 | | 96,01 | |
|  | |  | |  | |  | |  | |  | |  | |  | |  | |  | |  | |  | |  | |  | |  | |  | |
|  | |  | | **[1]** | | **[2]** | | **[3]** | | **[4]** | | **[5]** | | **[6]** | | **[7]** | | **[8]** | | **[9]** | | **[10]** | | **[11]** | | **[12]** | | **[13]** | | **[14]** | |
| **[51]** | | *Actinomadura rudentiformis* | | 94,58 | | 94,79 | | 96,86 | | 94,94 | | 97,36 | | 94,79 | | 96,36 | | 96,58 | | 96,15 | | 96,01 | | 97,43 | | 95,58 | | 96,93 | | 95,72 | |
| **[52]** | | *Actinomadura rupiterrae* | | 93,22 | | 94,22 | | 94,72 | | 94,58 | | 95,01 | | 94,44 | | 93,94 | | 94,37 | | 93,72 | | 93,79 | | 94,72 | | 94,65 | | 94,79 | | 95,22 | |
| **[53]** | | *Actinomadura verrucospora* | | 93,44 | | 94,51 | | 96,43 | | 95,72 | | 97,65 | | 94,51 | | 96,58 | | 98,36 | | 99,14 | | 96,5 | | 96,72 | | 94,86 | | 96,86 | | 95,44 | |
| **[54]** | | *Actinomadura viridis* | | 93,65 | | 95,01 | | 95,36 | | 94,86 | | 97 | | 94,79 | | 96,15 | | 96,72 | | 96,15 | | 95,44 | | 96,36 | | 94,86 | | 96,08 | | 95,29 | |
| **[55]** | | *Actinomadura syzygii* | | 93,15 | | 94,65 | | 95,72 | | 95,01 | | 97,65 | | 94,94 | | 96,65 | | 97 | | 96,58 | | 95,72 | | 96,86 | | 95,22 | | 96,15 | | 95,36 | |
| **[56]** | | *LGMB487* | | 94,94 | | 94,94 | | 96,93 | | 94,72 | | 97,22 | | 95,08 | | 96,29 | | 96,36 | | 95,93 | | 95,93 | | 97,36 | | 95,72 | | 96,93 | | 95,79 | |
| **[57]** | | *LGMB466* | | 95,22 | | 95,36 | | 97,15 | | 95,44 | | 97,43 | | 95,51 | | 96,5 | | 96,58 | | 96,15 | | 96,15 | | 97,43 | | 96,29 | | 97,65 | | 96,5 | |
| **[58]** | | *Streptomyces glauciniger* | | 89,59 | | 89,94 | | 90,09 | | 89,23 | | 90,01 | | 89,44 | | 89,3 | | 89,94 | | 89,66 | | 89,59 | | 89,8 | | 90,01 | | 90,73 | | 90,16 | |

Table S1- Similarity percentage matrix for the gene 16S rRNA, of LGMB466 and 487- *Actinomadura* genus

|  |  | **[15]** | **[16]** | **[17]** | **[18]** | **[19]** | **[20]** | **[21]** | **[22]** | **[23]** | **[24]** | **[25]** | **[26]** | **[27]** | **[28]** |
| --- | --- | --- | --- | --- | --- | --- | --- | --- | --- | --- | --- | --- | --- | --- | --- |
| **[16]** | *Actinomadura geliboluensis* | 98,5 |  |  |  |  |  |  |  |  |  |  |  |  |  |
| **[17]** | *Actinomadura glauciflava* | 97,93 | 98,15 |  |  |  |  |  |  |  |  |  |  |  |  |
| **[18]** | *Actinomadura hallensis* | 94,86 | 96,15 | 95,36 |  |  |  |  |  |  |  |  |  |  |  |
| **[19]** | *Actinomadura keratinilytica* | 95,01 | 95,86 | 95,22 | 94,94 |  |  |  |  |  |  |  |  |  |  |
| **[20]** | *Actinomadura kijaniata* | 95,15 | 96,01 | 96,01 | 94,01 | 95,79 |  |  |  |  |  |  |  |  |  |
| **[21]** | *Actinomadura latina* | 96,72 | 97,57 | 96,79 | 95,22 | 94,94 | 95,72 |  |  |  |  |  |  |  |  |
| **[22]** | *Actinomadura luteofluorescens* | 97,08 | 97,57 | 99,14 | 94,79 | 94,58 | 95,44 | 96,15 |  |  |  |  |  |  |  |
| **[23]** | *Actinomadura macra* | 96,65 | 97,22 | 97,79 | 94,79 | 94,44 | 94,86 | 96,36 | 97,93 |  |  |  |  |  |  |
| **[24]** | *Actinomadura madurae* | 97,29 | 98,15 | 97,93 | 95,44 | 95,22 | 96,22 | 97,65 | 97,36 | 97,22 |  |  |  |  |  |
| **[25]** | *Actinomadura mexicana* | 97,93 | 97,93 | 99,29 | 95,29 | 95,08 | 95,72 | 97,08 | 98,43 | 97,5 | 98,07 |  |  |  |  |
| **[26]** | *Actinomadura namibiensis* | 95,08 | 95,93 | 95,86 | 94,08 | 95,93 | 99,5 | 95,72 | 95,22 | 94,72 | 96,15 | 95,58 |  |  |  |
| **[27]** | *Actinomadura meyerae* | 97,5 | 98,72 | 98,22 | 95,86 | 95,44 | 96,29 | 97,36 | 97,86 | 97,22 | 98 | 98 | 96,15 |  |  |
| **[28]** | *Actinomadura nitritigenes* | 96,43 | 97,5 | 97,43 | 95,01 | 96,5 | 96,93 | 96,65 | 96,79 | 96,22 | 97,15 | 97,36 | 96,79 | 97,5 |  |
| **[29]** | *Actinomadura miaoliensis* | 95,08 | 96,08 | 95,51 | 94,65 | 98,64 | 96,22 | 95,36 | 94,86 | 94,79 | 95,44 | 95,36 | 96,29 | 95,93 | 97 |

Table S1- Similarity percentage matrix for the gene 16S rRNA, of LGMB466 and 487- *Actinomadura* genus

|  |  | **[15]** | **[16]** | **[17]** | **[18]** | **[19]** | **[20]** | **[21]** | **[22]** | **[23]** | **[24]** | **[25]** | **[26]** | **[27]** | **[28]** |
| --- | --- | --- | --- | --- | --- | --- | --- | --- | --- | --- | --- | --- | --- | --- | --- |
| **[30]** | *Actinomadura rifamycini* | 95,72 | 96,15 | 96,22 | 93,87 | 94,44 | 95,15 | 95,08 | 96,29 | 95,51 | 95,86 | 95,86 | 95,08 | 96,01 | 95,51 |
| **[31]** | *Actinomadura rayongensis* | 95,29 | 95,72 | 96,36 | 94,15 | 95,51 | 95,65 | 94,94 | 95,58 | 95,08 | 95,36 | 96,08 | 95,51 | 95,72 | 95,65 |
| **[32]** | *Actinomadura napierensis* | 97,15 | 97,93 | 97,57 | 95,72 | 95,36 | 96,36 | 97,86 | 96,93 | 96,65 | 97,79 | 97,57 | 96,36 | 97,86 | 97,29 |
| **[33]** | *Actinomadura rugatobispora* | 95,58 | 95,93 | 96,15 | 93,79 | 94,01 | 95,58 | 95,08 | 96,29 | 95,58 | 96,08 | 95,79 | 95,36 | 96,43 | 95,22 |
| **[34]** | *Actinomadura rubrobrunea* | 93,51 | 94,37 | 94,08 | 93,65 | 97,36 | 95,36 | 93,87 | 93,51 | 93,44 | 94,58 | 93,87 | 95,51 | 94,51 | 95,22 |
| **[35]** | *Actinomadura scrupuli* | 94,01 | 94,65 | 94,79 | 93,3 | 94,22 | 95,22 | 94,29 | 94,22 | 94,15 | 94,65 | 94,51 | 95,22 | 94,72 | 95,86 |
| **[36]** | *Actinomadura umbrina* | 94,15 | 94,94 | 94,79 | 93,65 | 93,65 | 94,58 | 94,72 | 94,29 | 94,22 | 95,08 | 94,58 | 94,51 | 95,22 | 95,15 |
| **[37]** | *Actinomadura sputi* | 94,94 | 96,22 | 95,51 | 98,29 | 94,72 | 95,01 | 95,51 | 94,94 | 94,94 | 95,93 | 95,29 | 95,01 | 96,22 | 95,15 |
| **[38]** | *Actinomadura viridilutea* | 93,44 | 94,29 | 94,01 | 93,58 | 97,29 | 95,29 | 93,87 | 93,44 | 93,37 | 94,51 | 93,79 | 95,44 | 94,44 | 95,15 |
| **[39]** | *Actinomadura sediminis* | 96,22 | 96,79 | 96,58 | 94,29 | 93,87 | 95,15 | 95,72 | 95,79 | 95,29 | 96,43 | 96,22 | 95,08 | 96,5 | 95,15 |
| **[40]** | *Actinomadura yumaensis* | 95,58 | 96,58 | 97 | 95,01 | 94,44 | 95,15 | 95,51 | 96,43 | 95,44 | 96,22 | 96,72 | 95,08 | 96,86 | 96,22 |
| **[41]** | *Actinomadura vinacea* | 96,22 | 96,5 | 96,72 | 94,94 | 94,58 | 95,65 | 95,58 | 96,22 | 95,58 | 96,58 | 96,65 | 95,51 | 96,65 | 96,01 |
| **[42]** | *Actinomadura xylanilytica* | 96,65 | 97,57 | 98 | 95,29 | 95,86 | 96,43 | 96,93 | 97,36 | 96,65 | 97,43 | 97,72 | 96,29 | 97,5 | 98,29 |
| **[43]** | *Actinomadura livida* | 95,65 | 96,36 | 96,36 | 95,65 | 95,15 | 94,65 | 95,58 | 95,79 | 95,36 | 95,93 | 96,29 | 94,58 | 96,22 | 95,72 |
| **[44]** | *Actinomadura hibisca* | 95,36 | 96,22 | 96,36 | 94,22 | 96,01 | 97,57 | 95,29 | 95,72 | 95,01 | 95,93 | 95,93 | 97,57 | 96,15 | 97 |
| **[45]** | *Actinomadura meridiana* | 96,79 | 97 | 96,86 | 93,94 | 94,08 | 94,65 | 95,93 | 96,08 | 95,86 | 96,5 | 96,86 | 94,58 | 96,58 | 95,72 |
| **[46]** | *Actinomadura fulvescens* | 97 | 97,65 | 97,29 | 94,86 | 95,58 | 96,22 | 96,5 | 96,65 | 96,15 | 96,93 | 97,22 | 96,08 | 97,5 | 97,65 |
| **[47]** | *Actinomadura chibensis* | 95,51 | 96,5 | 96,15 | 94,58 | 94,65 | 94,58 | 95,72 | 95,51 | 95,58 | 95,86 | 95,86 | 94,65 | 96,5 | 95,72 |
| **[48]** | *Actinomadura catellatispora* | 93,58 | 94,29 | 94,29 | 93,58 | 93,15 | 92,58 | 93,79 | 93,72 | 93,3 | 93,87 | 94,22 | 92,51 | 94,29 | 93,65 |
| **[49]** | *Actinomadura pelletieri* | 95,36 | 95,93 | 95,93 | 94,01 | 94,01 | 93,79 | 95,22 | 95,15 | 95,65 | 95,65 | 95,72 | 94,01 | 95,79 | 94,79 |
| **[50]** | *Actinomadura oligospora* | 95,08 | 95,86 | 95,58 | 93,94 | 96,15 | 96,43 | 95,15 | 94,86 | 94,86 | 95,36 | 95,58 | 96,5 | 95,79 | 96,22 |
| **[51]** | *Actinomadura rudentiformis* | 96,58 | 97,08 | 96,72 | 94,58 | 95,58 | 96,15 | 96,5 | 96,01 | 95,93 | 97,72 | 96,58 | 96,08 | 97,15 | 97,22 |
| **[52]** | *Actinomadura rupiterrae* | 93,65 | 94,65 | 94,29 | 93,58 | 94,86 | 95,51 | 94,44 | 93,65 | 93,44 | 95,22 | 94,29 | 95,58 | 94,79 | 95,08 |
| **[53]** | *Actinomadura verrucospora* | 97,15 | 97,5 | 98,72 | 94,72 | 94,44 | 95,29 | 96,08 | 99,29 | 97,93 | 97,29 | 98,15 | 95,08 | 97,86 | 96,79 |
| **[54]** | *Actinomadura viridis* | 96,22 | 96,5 | 96,72 | 95,08 | 94,51 | 95,44 | 95,65 | 96,22 | 95,51 | 96,58 | 96,65 | 95,29 | 96,58 | 95,93 |

Table S1- Similarity percentage matrix for the gene 16S rRNA, of LGMB466 and 487- *Actinomadura* genus

|  |  | **[15]** | **[16]** | **[17]** | **[18]** | **[19]** | **[20]** | **[21]** | **[22]** | **[23]** | **[24]** | **[25]** | **[26]** | **[27]** | **[28]** |
| --- | --- | --- | --- | --- | --- | --- | --- | --- | --- | --- | --- | --- | --- | --- | --- |
| **[55]** | *Actinomadura syzygii* | 96,65 | 97,65 | 97,15 | 94,58 | 95,36 | 95,44 | 96,43 | 96,5 | 96,36 | 96,65 | 96,72 | 95,36 | 97,43 | 96,65 |
| **[56]** | *LGMB487* | 95,86 | 96,79 | 96,5 | 94,58 | 95,51 | 96,22 | 96,79 | 95,86 | 95,79 | 97,29 | 96,36 | 96,08 | 96,79 | 97,5 |
| **[57]** | *LGMB466* | 96,08 | 97 | 96,72 | 94,79 | 96,22 | 96,79 | 96,93 | 96,08 | 96,01 | 97,5 | 96,58 | 96,65 | 97 | 98,36 |
| **[58]** | *Streptomyces glauciniger* | 89,51 | 89,8 | 90,09 | 87,52 | 89,8 | 90,66 | 89,44 | 89,44 | 89,23 | 90,01 | 89,87 | 90,66 | 89,66 | 90,51 |

Table S1- Similarity percentage matrix for the gene 16S rRNA, of LGMB466 and 487- *Actinomadura* genus

|  |  | **[29]** | **[30]** | **[31]** | **[32]** | **[33]** | **[34]** | **[35]** | **[36]** | **[37]** | **[38]** | **[39]** | **[40]** | **[41]** | **[42]** |
| --- | --- | --- | --- | --- | --- | --- | --- | --- | --- | --- | --- | --- | --- | --- | --- |
| [30] | Actinomadura rifamycini | 94,15 |  |  |  |  |  |  |  |  |  |  |  |  |  |
| [31] | Actinomadura rayongensis | 95,65 | 93,87 |  |  |  |  |  |  |  |  |  |  |  |  |
| [32] | Actinomadura napierensis | 95,93 | 95,65 | 95,51 |  |  |  |  |  |  |  |  |  |  |  |
| [33] | Actinomadura rugatobispora | 94,29 | 94,58 | 94,51 | 95,86 |  |  |  |  |  |  |  |  |  |  |
| [34] | Actinomadura rubrobrunea | 96,5 | 93,65 | 94,44 | 94,72 | 93,58 |  |  |  |  |  |  |  |  |  |
| [35] | Actinomadura scrupuli | 94,51 | 94,01 | 93,37 | 95,08 | 93,44 | 93,3 |  |  |  |  |  |  |  |  |
| [36] | Actinomadura umbrina | 94,08 | 93,22 | 94,08 | 95,08 | 93,65 | 92,87 | 94,94 |  |  |  |  |  |  |  |
| [37] | Actinomadura sputi | 94,72 | 94,15 | 94,37 | 96,15 | 94,72 | 94,58 | 93,72 | 93,94 |  |  |  |  |  |  |
| [38] | Actinomadura viridilutea | 96,43 | 93,58 | 94,37 | 94,65 | 93,58 | 99,43 | 93,22 | 92,8 | 94,51 |  |  |  |  |  |
| [39] | Actinomadura sediminis | 93,94 | 96,15 | 94,58 | 96,22 | 94,58 | 93,79 | 93,37 | 93,51 | 95,15 | 93,58 |  |  |  |  |
| [40] | Actinomadura yumaensis | 94,58 | 94,29 | 94,37 | 96,43 | 97 | 93,37 | 94,29 | 94,51 | 95,15 | 93,3 | 94,44 |  |  |  |
| [41] | Actinomadura vinacea | 94,94 | 94,29 | 95,08 | 96,08 | 98,15 | 93,79 | 94,15 | 94,44 | 95,29 | 93,72 | 94,79 | 98,15 |  |  |
| [42] | Actinomadura xylanilytica | 96,29 | 95,01 | 96,36 | 97,72 | 95,65 | 94,72 | 95,29 | 95,51 | 95,29 | 94,65 | 95,58 | 96,5 | 96,29 |  |
| [43] | Actinomadura livida | 95,01 | 94,15 | 94,58 | 96,22 | 96,36 | 93,87 | 94,51 | 94,65 | 95,51 | 93,79 | 94,15 | 97,57 | 97,43 | 96,15 |
| [44] | Actinomadura hibisca | 96,5 | 94,94 | 95,58 | 96,01 | 95,29 | 95,08 | 94,51 | 93,79 | 94,58 | 95,01 | 94,72 | 95,72 | 95,79 | 96,86 |
| [45] | Actinomadura meridiana | 94,08 | 94,79 | 94,72 | 95,93 | 94,86 | 93,01 | 93,87 | 94,22 | 94,15 | 92,94 | 95,36 | 95,36 | 95,44 | 95,86 |
| [46] | Actinomadura fulvescens | 95,93 | 95,44 | 95,15 | 97,15 | 96,01 | 94,72 | 95,08 | 94,29 | 95,15 | 94,65 | 95,36 | 96,22 | 96,5 | 97,43 |
| [47] | Actinomadura chibensis | 95,08 | 94,01 | 94,15 | 96,36 | 94,72 | 93,87 | 94,08 | 95,15 | 94,79 | 93,79 | 94,29 | 96,29 | 95,58 | 96,01 |
| [48] | Actinomadura catellatispora | 93,01 | 92,01 | 92,72 | 94,15 | 94,01 | 91,87 | 92,44 | 92,58 | 93,44 | 91,8 | 92,08 | 95,22 | 95,08 | 94,08 |

Table S1- Similarity percentage matrix for the gene 16S rRNA, of LGMB466 and 487- *Actinomadura* genus

|  |  | **[29]** | **[30]** | **[31]** | **[32]** | **[33]** | **[34]** | **[35]** | **[36]** | **[37]** | **[38]** | **[39]** | **[40]** | **[41]** | **[42]** |
| --- | --- | --- | --- | --- | --- | --- | --- | --- | --- | --- | --- | --- | --- | --- | --- |
| [49] | *Actinomadura pelletieri* | 93,94 | 94,29 | 94,08 | 95,29 | 94,58 | 92,94 | 93,44 | 93,72 | 94,51 | 92,87 | 94,65 | 94,86 | 94,58 | 95,08 |
| [50] | *Actinomadura oligospora* | 96,15 | 94,51 | 95,29 | 95,79 | 95,08 | 95,15 | 94,37 | 93,3 | 94,37 | 95,08 | 93,87 | 95,01 | 95,29 | 95,79 |
| [51] | *Actinomadura rudentiformis* | 95,79 | 95,65 | 94,58 | 96,72 | 95,93 | 94,86 | 94,72 | 94,79 | 95,08 | 94,79 | 95,51 | 95,93 | 96,15 | 96,65 |
| [52] | *Actinomadura rupiterrae* | 95,22 | 93,51 | 94,58 | 94,44 | 94,29 | 94,37 | 93,65 | 93,65 | 94,22 | 94,29 | 93,3 | 94,58 | 94,94 | 94,44 |
| [53] | *Actinomadura verrucospora* | 94,72 | 96,08 | 95,44 | 96,86 | 96,29 | 93,37 | 94,37 | 94,29 | 94,86 | 93,3 | 95,58 | 96,29 | 96,22 | 97,15 |
| [54] | *Actinomadura viridis* | 95,01 | 94,29 | 95,29 | 96,08 | 97,72 | 93,44 | 94,29 | 94,44 | 95,08 | 93,37 | 94,94 | 98 | 99,36 | 96,43 |
| [55] | *Actinomadura syzygii* | 96,01 | 94,51 | 95,01 | 96,86 | 95,65 | 94,01 | 94,01 | 94,79 | 94,86 | 93,94 | 95,29 | 96,08 | 96,08 | 97 |
| [56] | *LGMB487* | 96,15 | 95,58 | 94,51 | 97,15 | 95,36 | 94,58 | 95,79 | 95,15 | 95,08 | 94,51 | 94,79 | 95,79 | 95,65 | 96,93 |
| [57] | *LGMB466* | 96,86 | 95,79 | 95,22 | 97,43 | 95,29 | 95,29 | 96,22 | 95,58 | 95,29 | 95,22 | 95,01 | 95,72 | 95,58 | 97,72 |
| [58] | *Streptomyces glauciniger* | 90,09 | 89,37 | 89,44 | 90,23 | 88,73 | 90,3 | 90,51 | 89,44 | 88,09 | 90,16 | 88,87 | 88,8 | 88,66 | 90,37 |

Table S1- Similarity percentage matrix for the gene 16S rRNA, of LGMB466 and 487- *Actinomadura* genus

|  |  | **[43]** | **[44]** | **[45]** | **[46]** | **[47]** | **[48]** | **[49]** | **[50]** | **[51]** | **[52]** | **[53]** | **[54]** | **[55]** | **[56]** | **[57]** | **[58]** |
| --- | --- | --- | --- | --- | --- | --- | --- | --- | --- | --- | --- | --- | --- | --- | --- | --- | --- |
| [43] | *Actinomadura livida* | 0 |  |  |  |  |  |  |  |  |  |  |  |  |  |  |  |
| [44] | *Actinomadura hibisca* | 95,15 |  |  |  |  |  |  |  |  |  |  |  |  |  |  |  |
| [45] | *Actinomadura meridiana* | 95,58 | 95,22 |  |  |  |  |  |  |  |  |  |  |  |  |  |  |
| [46] | *Actinomadura fulvescens* | 96,43 | 97,5 | 96,79 |  |  |  |  |  |  |  |  |  |  |  |  |  |
| [47] | *Actinomadura chibensis* | 96,15 | 95,44 | 96,08 | 96,29 |  |  |  |  |  |  |  |  |  |  |  |  |
| [48] | *Actinomadura catellatispora* | 97,65 | 93,08 | 93,51 | 94,37 | 94,15 |  |  |  |  |  |  |  |  |  |  |  |
| [49] | *Actinomadura pelletieri* | 95,36 | 94,58 | 96,65 | 95,58 | 96,22 | 93,3 |  |  |  |  |  |  |  |  |  |  |
| [50] | *Actinomadura oligospora* | 95,29 | 96,58 | 95,01 | 96,65 | 95,36 | 93,22 | 94,79 |  |  |  |  |  |  |  |  |  |
| [51] | *Actinomadura rudentiformis* | 95,86 | 97,15 | 96,43 | 98,64 | 96,08 | 93,79 | 95,79 | 95,93 |  |  |  |  |  |  |  |  |
| [52] | *Actinomadura rupiterrae* | 94,72 | 95,79 | 94,08 | 95,01 | 94,44 | 92,72 | 93,65 | 96,72 | 95,72 |  |  |  |  |  |  |  |
| [53] | *Actinomadura verrucospora* | 95,79 | 95,72 | 96,15 | 96,79 | 95,51 | 93,72 | 95,15 | 94,86 | 96,15 | 93,65 |  |  |  |  |  |  |
| [54] | *Actinomadura viridis* | 97,5 | 95,86 | 95,58 | 96,43 | 95,58 | 95,15 | 94,51 | 95,01 | 96,01 | 94,86 | 96,22 |  |  |  |  |  |
| [55] | *Actinomadura syzygii* | 96,01 | 96,5 | 96,79 | 97,29 | 98,57 | 94,01 | 96,58 | 95,51 | 96,86 | 94,58 | 96,5 | 96,01 |  |  |  |  |

Table S1- Similarity percentage matrix for the gene 16S rRNA, of LGMB466 and 487- *Actinomadura* genus

|  |  | **[43]** | **[44]** | **[45]** | **[46]** | **[47]** | **[48]** | **[49]** | **[50]** | **[51]** | **[52]** | **[53]** | **[54]** | **[55]** | **[56]** | **[57]** | **[58]** |
| --- | --- | --- | --- | --- | --- | --- | --- | --- | --- | --- | --- | --- | --- | --- | --- | --- | --- |
| [56] | *LGMB487* | 96,01 | 96,5 | 95,58 | 97,57 | 95,86 | 93,94 | 95,36 | 96,5 | 98 | 95,65 | 95,86 | 95,65 | 96,29 |  |  |  |
| [57] | *LGMB466* | 95,79 | 96,5 | 95,44 | 97,29 | 95,72 | 93,72 | 95,22 | 96,29 | 97,72 | 95,44 | 95,93 | 95,58 | 96,15 | **98,86** |  |  |
| [58] | *Streptomyces glauciniger* | 89,02 | 89,87 | 89,23 | 90,16 | 88,73 | 87,02 | 88,37 | 89,44 | 89,94 | 89,23 | 89,51 | 88,73 | 89,16 | 90,37 | 90,8 | 0 |

Table S2- Similarity percentage matrix for the gene 16S rRNA, of LGMB491 - *Aeromicrobium* genus

|  |  | **[1]** | **[2]** | **[3]** | **[4]** | **[5]** | **[6]** | **[7]** | **[8]** | **[9]** | **[10]** | **[11]** | **[12]** | **[13]** | **[14]** |
| --- | --- | --- | --- | --- | --- | --- | --- | --- | --- | --- | --- | --- | --- | --- | --- |
| **[1]** | *Aeromicrobium alkaliterrae* |  |  |  |  |  |  |  |  |  |  |  |  |  |  |
| **[2]** | *Aeromicrobium erythreum* | 97,6 |  |  |  |  |  |  |  |  |  |  |  |  |  |
| **[3]** | *Aeromicrobium fastidiosum* | 97,6 | 98,13 |  |  |  |  |  |  |  |  |  |  |  |  |
| **[4]** | *Aeromicrobium flavum* | 96,33 | 96,33 | 96,03 |  |  |  |  |  |  |  |  |  |  |  |
| **[5]** | *Aeromicrobium ginsengisoli* | 98,43 | 97,38 | 97,75 | 96,86 |  |  |  |  |  |  |  |  |  |  |
| **[6]** | *Aeromicrobium halocynthiae* | 97,38 | 97,46 | 96,11 | 96,11 | 97,68 |  |  |  |  |  |  |  |  |  |
| **[7]** | *Aeromicrobium marinum* | 98,13 | 96,93 | 97,46 | 96,26 | 99,1 | 97,38 |  |  |  |  |  |  |  |  |
| **[8]** | *Aeromicrobium massiliense* | 96,26 | 96,26 | 95,88 | 95,66 | 95,96 | 95,06 | 95,21 |  |  |  |  |  |  |  |
| **[9]** | *Aeromicrobium panaciterrae* | 97,68 | 96,78 | 97,01 | 96,93 | 98,95 | 96,93 | 98,2 | 95,96 |  |  |  |  |  |  |
| **[10]** | *Aeromicrobium tamlense* | 96,33 | 96,48 | 96,11 | 98,88 | 96,71 | 96,41 | 96,41 | 95,43 | 96,63 |  |  |  |  |  |
| **[11]** | *Aeromicrobium camelliae* | 96,63 | 96,78 | 96,41 | 95,36 | 95,88 | 95,88 | 95,43 | 95,58 | 95,28 | 95,73 |  |  |  |  |
| **[12]** | *Nocardioides albus* | 91,84 | 91,77 | 92,37 | 92,07 | 91,99 | 91,17 | 91,39 | 92,81 | 92,22 | 92,14 | 92,14 |  |  |  |
| **[13]** | *Aeromicrobium ponti* | 97,83 | 98,2 | 96,78 | 95,66 | 97,23 | 97,31 | 97,23 | 95,73 | 96,48 | 96,11 | 95,96 | 91,32 |  |  |
| **[14]** | *LGMB491* | 98,2 | 98,35 | 97,08 | 96,41 | 97,9 | 97,9 | 97,75 | 95,81 | 97,16 | 96,18 | 95,88 | 91,17 | **99,25** |  |

Table S3- Similarity percentage matrix for the gene 16S rRNA, of LGMB471 - *Microbacterium*sp.

|  |  | **[1]** | **[2]** | **[3]** | **[4]** | **[5]** | **[6]** | **[7]** | **[8]** |
| --- | --- | --- | --- | --- | --- | --- | --- | --- | --- |
| **[1]** | *Agrococcus jenensis* | 0 | 93,42 | 93 | 91,67 | 93,21 | 93,11 | 92,9 | 92,08 |
| **[2]** | *LGMB471* | 93,42 | 0 | 98,66 | 97,63 | 99,07 | 98,97 | 98,87 | 97,94 |
| **[3]** | *Microbacterium liquefaciens* | 93 | 98,66 | 0 | 97,84 | 99,59 | 99,49 | 98,77 | 98,25 |
| **[4]** | *Microbacterium luteolum* | 91,67 | 97,63 | 97,84 | 0 | 98,25 | 98,15 | 98,15 | 97,74 |
| **[5]** | *Microbacterium maritypicum* | 93,21 | 99,07 | 99,59 | 98,25 | 0 | 99,9 | 99,18 | 98,66 |
| **[6]** | *Microbacterium oxydans* | 93,11 | 98,97 | 99,49 | 98,15 | 99,9 | 0 | 99,07 | 98,56 |
| **[7]** | *Microbacterium paraoxydans* | 92,9 | 98,87 | 98,77 | 98,15 | 99,18 | 99,07 | 0 | 98,46 |
| **[8]** | *Microbacterium saperdae* | 92,08 | 97,94 | 98,25 | 97,74 | 98,66 | 98,56 | 98,46 | 0 |

Table S4- Similarity percentage matrix for the gene 16S rRNA, of LGMB461 and LGMB465 - *Microbispora* genus

|  |  | **[1]** | **[2]** | **[3]** | **[4]** | **[5]** | **[6]** | **[7]** | **[8]** | **[9]** | **[10]** | **[11]** | **[12]** | **[13]** | **[14]** |
| --- | --- | --- | --- | --- | --- | --- | --- | --- | --- | --- | --- | --- | --- | --- | --- |
| [1] | *LGMB461* |  |  |  |  |  |  |  |  |  |  |  |  |  |  |
| [2] | *LGMB465* | **99,84** |  |  |  |  |  |  |  |  |  |  |  |  |  |
| [3] | *Microbispora chromogenes* | 97,63 | 97,78 |  |  |  |  |  |  |  |  |  |  |  |  |
| [4] | *Microbispora indica* | 99,37 | 99,53 | 97,63 |  |  |  |  |  |  |  |  |  |  |  |
| [5] | *Microbispora karnatakensis* | 99,21 | 99,37 | 97,15 | 99,21 |  |  |  |  |  |  |  |  |  |  |
| [6] | *Microbispora parva* | 98,58 | 98,73 | 97,31 | 98,58 | 98,73 |  |  |  |  |  |  |  |  |  |
| [7] | *Microbispora thermodiastatica* | 97,47 | 97,63 | 95,89 | 97,47 | 97,63 | 97,63 |  |  |  |  |  |  |  |  |
| [8] | *Microbispora thermorosea* | 96,04 | 96,2 | 97,31 | 96,04 | 96,2 | 95,89 | 96,68 |  |  |  |  |  |  |  |
| [9] | *Microbispora amethystogenes* | 98,42 | 98,58 | 96,99 | 98,73 | 98,58 | 98,89 | 97,47 | 95,41 |  |  |  |  |  |  |
| [10] | *Microbispora corallina* | 97,94 | 98,1 | 97,78 | 97,94 | 98,1 | 97,78 | 96,84 | 97,15 | 97,31 |  |  |  |  |  |
| [11] | *Microbispora mesophila* | 92,25 | 92,41 | 91,46 | 92,56 | 92,09 | 91,61 | 91,3 | 90,82 | 91,77 | 92,56 |  |  |  |  |
| [12] | *Microbispora aerata* | 98,1 | 98,26 | 96,52 | 98,1 | 98,26 | 98,26 | 99,21 | 96,84 | 98,1 | 97,47 | 91,61 |  |  |  |
| [13] | *Microbispora rosea* | 98,73 | 98,89 | 96,99 | 99,37 | 98,58 | 97,94 | 97,31 | 95,57 | 98,1 | 97,31 | 92,25 | 97,78 |  |  |
| [14] | *Microbispora siamensis* | 96,68 | 96,84 | 98,1 | 96,68 | 96,84 | 96,68 | 95,57 | 96,68 | 96,04 | 97,15 | 90,35 | 96,2 | 96,04 |  |
| [15] | *LGMB250* | 99,68 | 99,84 | 97,94 | 99,68 | 99,21 | 98,58 | 97,47 | 96,04 | 98,73 | 97,94 | 92,56 | 98,1 | 99,05 | 96,68 |
| [16] | *LGMB251* | 99,53 | 99,68 | 97,78 | 99,53 | 99,05 | 98,42 | 97,31 | 96,04 | 98,58 | 97,78 | 92,56 | 97,94 | 98,89 | 96,52 |
| [17] | *LGMB252* | 69,78 | 69,94 | 67,88 | 69,62 | 69,62 | 68,67 | 68,04 | 67,09 | 68,83 | 68,04 | 64,08 | 68,67 | 69,15 | 67,72 |
| [18] | *LGMB253* | 69,94 | 70,09 | 68,35 | 69,78 | 69,78 | 69,15 | 68,2 | 67,25 | 69,15 | 68,2 | 64,4 | 68,83 | 69,3 | 67,88 |
| [19] | *LGMB255* | 99,21 | 99,37 | 97,47 | 99,21 | 98,73 | 98,1 | 96,99 | 95,57 | 98,26 | 97,47 | 92,25 | 97,63 | 98,58 | 96,2 |
| [20] | *LGMB256* | 97,47 | 97,63 | 99,21 | 97,47 | 96,99 | 96,68 | 95,73 | 97,15 | 96,52 | 97,63 | 91,14 | 96,36 | 96,84 | 97,63 |
| [21] | *LGMB257* | 95,09 | 95,25 | 96,99 | 95,09 | 94,62 | 94,62 | 93,67 | 95,25 | 94,15 | 95,57 | 89,4 | 94,3 | 94,46 | 95,41 |
| [22] | *LGMB258* | 99,05 | 99,21 | 96,99 | 98,73 | 98,58 | 97,94 | 96,84 | 95,41 | 97,78 | 97,31 | 91,61 | 97,47 | 98,1 | 96,04 |
| [23] | *LGMB259* | **99,84** | **100** | 97,78 | 99,53 | 99,37 | 98,73 | 97,63 | 96,2 | 98,58 | 98,1 | 92,41 | 98,26 | 98,89 | 96,84 |
| [24] | *LGMB260* | 99,68 | 99,84 | 97,94 | 99,68 | 99,21 | 98,58 | 97,47 | 96,04 | 98,73 | 97,94 | 92,56 | 98,1 | 99,05 | 96,68 |
| [25] | *LGMB261* | 99,53 | 99,68 | 97,78 | 99,53 | 99,05 | 98,42 | 97,31 | 95,89 | 98,58 | 97,78 | 92,41 | 97,94 | 98,89 | 96,52 |
| [26] | *Actinomadura echinospora* | 93,83 | 93,99 | 93,35 | 93,99 | 93,51 | 93,67 | 93,51 | 92,72 | 93,83 | 93,99 | 93,35 | 93,99 | 93,51 | 92,09 |

Table S4- Similarity percentage matrix for the gene 16S rRNA, of LGMB461 and LGMB465 - *Microbispora* genus

|  |  | **[15]** | **[16]** | **[17]** | **[18]** | **[19]** | **[20]** | **[21]** | **[22]** | **[23]** | **[24]** | **[25]** | **[26]** |
| --- | --- | --- | --- | --- | --- | --- | --- | --- | --- | --- | --- | --- | --- |
| [16] | LGMB251 | 99,84 |  |  |  |  |  |  |  |  |  |  |  |
| [17] | LGMB252 | 69,94 | 70,09 |  |  |  |  |  |  |  |  |  |  |
| [18] | LGMB253 | 70,09 | 70,25 | 99,21 |  |  |  |  |  |  |  |  |  |
| [19] | LGMB255 | 99,53 | 99,37 | 69,62 | 69,78 |  |  |  |  |  |  |  |  |
| [20] | LGMB256 | 97,78 | 97,63 | 67,72 | 67,88 | 97,31 |  |  |  |  |  |  |  |
| [21] | LGMB257 | 95,41 | 95,41 | 65,98 | 66,3 | 94,94 | 97,63 |  |  |  |  |  |  |
| [22] | LGMB258 | 99,05 | 98,89 | 69,3 | 69,46 | 98,58 | 97,78 | 95,41 |  |  |  |  |  |
| [23] | LGMB259 | 99,84 | 99,68 | 69,94 | 70,09 | 99,37 | 97,63 | 95,25 | 99,21 |  |  |  |  |
| [24] | LGMB260 | 100 | 99,84 | 69,94 | 70,09 | 99,53 | 97,78 | 95,41 | 99,05 | 99,84 |  |  |  |
| [25] | LGMB261 | 99,84 | 99,68 | 69,78 | 69,94 | 99,37 | 97,63 | 95,25 | 98,89 | 99,68 | 99,84 |  |  |
| [26] | *Actinomadura echinospora* | 94,15 | 93,99 | 64,4 | 64,72 | 93,83 | 93,04 | 91,14 | 93,35 | 93,99 | 94,15 | 93,99 |  |

Table S5- Similarity percentage matrix for the gene 16S rRNA, of LGMB485 - *Micrococcus* genus

|  |  | **[1]** | **[2]** | **[3]** | **[4]** | **[5]** | **[6]** | **[7]** | **[8]** | **[9]** | **[10]** | **[11]** |
| --- | --- | --- | --- | --- | --- | --- | --- | --- | --- | --- | --- | --- |
| [1] | *LGMB 485* |  |  |  |  |  |  |  |  |  |  |  |
| [2] | *Micrococcus flavus* | 98,75 |  |  |  |  |  |  |  |  |  |  |
| [3] | *Micrococcus antarticus* | 99,17 | 97,92 |  |  |  |  |  |  |  |  |  |
| [4] | *Micrococcus lylae* | 98,96 | 97,71 | 98,12 |  |  |  |  |  |  |  |  |
| [5] | *Micrococcus aloeverae* | 100 | 98,75 | 99,17 | 98,96 |  |  |  |  |  |  |  |
| [6] | *Micrococcus cohnii* | 99,17 | 97,92 | 99,17 | 98,54 | 99,17 |  |  |  |  |  |  |
| [7] | *Micrococcus luteus* | 99,58 | 98,33 | 98,75 | 98,54 | 99,58 | 98,75 |  |  |  |  |  |
| [8] | *Micrococcus yunnanensis* | 100 | 98,75 | 99,17 | 98,96 | 100 | 99,17 | 99,58 |  |  |  |  |
| [9] | *Micrococcus terreus* | 97,92 | 97,08 | 97,92 | 97,71 | 97,92 | 98,33 | 97,92 | 97,92 |  |  |  |
| [10] | *Micrococcus endophyticus* | 99,79 | 98,54 | 98,96 | 98,75 | 99,79 | 98,96 | 99,38 | 99,79 | 97,71 |  |  |
| [11] | *Citricoccus parietis* | 97,71 | 96,46 | 97,71 | 97,5 | 97,71 | 97,71 | 97,29 | 97,71 | 97,71 | 97,5 | 0 |

|  |  | **[1]** | **[2]** | **[3]** | **[4]** | **[5]** | **[6]** | **[7]** | **[8]** | **[9]** | **[10]** | **[11]** | **[12]** |
| --- | --- | --- | --- | --- | --- | --- | --- | --- | --- | --- | --- | --- | --- |
| **[1]** | *LGMB 482* |  |  |  |  |  |  |  |  |  |  |  |  |
| **[2]** | *Sphaerisporangium melleum* | 99,4 |  |  |  |  |  |  |  |  |  |  |  |
| **[3]** | *Sphaerisporangium cinnabarium* | 98,89 | 98,49 |  |  |  |  |  |  |  |  |  |  |
| **[4]** | *Sphaerisporangium aureirubrum* | 96,18 | 95,98 | 96,68 |  |  |  |  |  |  |  |  |  |
| **[5]** | *Sphaerisporangium rufum* | 97,48 | 97,18 | 98,19 | 96,28 |  |  |  |  |  |  |  |  |
| **[6]** | *Sphaerisporangium album* | 96,48 | 96,38 | 96,98 | 96,48 | 97,69 |  |  |  |  |  |  |  |
| **[7]** | *Sphaerisporangium flaviroseum* | 96,98 | 97,18 | 97,89 | 96,68 | 97,18 | 97,38 |  |  |  |  |  |  |
| **[8]** | *Sphaerisporangium krabiense* | 97,79 | 98,19 | 97,69 | 96,18 | 97,38 | 97,08 | 97,08 |  |  |  |  |  |
| **[9]** | *Sphaerisporangium rubeum* | 96,18 | 96,18 | 96,68 | 98,29 | 96,78 | 96,78 | 96,38 | 96,28 |  |  |  |  |
| **[10]** | *Sphaerisporangium siamense* | 97,59 | 97,38 | 96,88 | 95,98 | 97,38 | 97,28 | 96,18 | 98,09 | 96,08 |  |  |  |
| **[11]** | *Sphaerisporangium viridalbum* | 97,69 | 98,09 | 96,98 | 95,77 | 96,78 | 96,28 | 97,59 | 97,28 | 96,18 | 97,28 |  |  |
| **[12]** | *Planobispora rosea* | 95,37 | 95,77 | 95,37 | 93,76 | 94,67 | 94,16 | 94,87 | 95,67 | 94,27 | 94,37 | 95,67 |  |

Table S6- Similarity percentage matrix for the gene 16S rRNA, of LGMB482 - *Sphaerisporangium* genus

Table S7- Similarity percentage matrix for the gene 16S rRNA, of LGMB483 - *Streptomyces* genus

|  |  | **[1]** | **[2]** | **[3]** | **[4]** | **[5]** | **[6]** | **[7]** | **[8]** | **[9]** | **[10]** | **[11]** | **[12]** | **[13]** | **[14]** |
| --- | --- | --- | --- | --- | --- | --- | --- | --- | --- | --- | --- | --- | --- | --- | --- |
| **[1]** | *LGMB483* |  |  |  |  |  |  |  |  |  |  |  |  |  |  |
| **[2]** | *Streptomyces prasinosporus* | 97,7 |  |  |  |  |  |  |  |  |  |  |  |  |  |
| **[3]** | *Streptomyces hyderabadensis* | 97,84 | 97,12 |  |  |  |  |  |  |  |  |  |  |  |  |
| **[4]** | *Streptomyces parvulus* | 98,63 | 97,7 | 98,85 |  |  |  |  |  |  |  |  |  |  |  |
| **[5]** | *Streptomyces spinoverrucosus* | 98,56 | 97,92 | 98,13 | 98,78 |  |  |  |  |  |  |  |  |  |  |
| **[6]** | *Streptomyces lomondensis* | 98,99 | 97,84 | 98,63 | 99,21 | 98,71 |  |  |  |  |  |  |  |  |  |
| **[7]** | *Streptomyces coerulescens* | 99,28 | 97,63 | 98,06 | 98,99 | 98,78 | 99,21 |  |  |  |  |  |  |  |  |
| **[8]** | *Streptomyces bellus* | 99,28 | 97,63 | 98,06 | 98,99 | 98,78 | 99,21 | 100 |  |  |  |  |  |  |  |
| **[9]** | *Streptomyces lusitanus* | 99,57 | 98,06 | 98,2 | 98,99 | 98,99 | 99,35 | 99,28 | 99,28 |  |  |  |  |  |  |
| **[10]** | *Streptomyces thermocarboxydus* | 99,86 | 97,7 | 97,84 | 98,63 | 98,56 | 98,99 | 99,28 | 99,28 | 99,57 |  |  |  |  |  |
| **[11]** | *Streptomyces speibonae* | 98,71 | 98,06 | 98,13 | 98,63 | 98,35 | 98,71 | 98,78 | 98,78 | 98,99 | 98,71 |  |  |  |  |
| **[12]** | *Streptomyces longispororuber* | 99,07 | 98,13 | 98,06 | 98,99 | 98,49 | 99,07 | 99,21 | 99,21 | 99,35 | 99,07 | 99,42 |  |  |  |
| **[13]** | *Streptomyces viridodiastaticus* | 98,78 | 97,63 | 98,35 | 98,71 | 98,35 | 98,71 | 99,14 | 99,14 | 99,07 | 98,78 | 99,07 | 99,42 |  |  |
| **[14]** | *Streptomyces albogriseolus* | 98,78 | 97,63 | 98,35 | 98,71 | 98,35 | 98,71 | 99,14 | 99,14 | 99,07 | 98,78 | 99,07 | 99,42 | 100 |  |
| **[15]** | *Streptomyces erythrogriseus* | 98,56 | 98,06 | 97,7 | 98,49 | 98,35 | 98,49 | 98,92 | 98,92 | 98,85 | 98,56 | 98,99 | 99,21 | 99,21 | 99,21 |
| **[16]** | *Streptomyces griseoincarnatus* | 98,56 | 98,06 | 97,7 | 98,49 | 98,35 | 98,49 | 98,92 | 98,92 | 98,85 | 98,56 | 98,99 | 99,21 | 99,21 | 99,21 |
| **[17]** | *Streptomyces griseosporeus* | 98,42 | 97,77 | 97,56 | 98,27 | 98,27 | 98,2 | 98,42 | 98,42 | 98,56 | 98,42 | 98,2 | 98,49 | 98,2 | 98,2 |
| **[18]** | *Streptomyces chromofuscus* | 98,2 | 98,63 | 97,99 | 98,42 | 98,63 | 98,56 | 98,42 | 98,42 | 98,56 | 98,2 | 98,78 | 98,56 | 98,2 | 98,2 |
| **[19]** | *Streptomyces glomeratus* | 98,06 | 97,92 | 97,92 | 98,35 | 98,13 | 98,49 | 98,2 | 98,2 | 98,42 | 98,06 | 98,63 | 98,49 | 98,27 | 98,27 |
| **[20]** | *Streptomyces chiangmaiensis* | 98,13 | 97,77 | 97,92 | 98,56 | 98,2 | 98,63 | 98,27 | 98,27 | 98,35 | 98,13 | 98,49 | 98,42 | 98,06 | 98,06 |
| **[21]** | *Streptomyces lannensis* | 97,77 | 97,41 | 97,56 | 98,06 | 97,84 | 97,99 | 97,63 | 97,63 | 97,99 | 97,77 | 98,35 | 98,13 | 97,77 | 97,77 |
| **[22]** | *Streptomyces leeuwenhoekii* | 98,27 | 97,48 | 98,13 | 98,63 | 98,42 | 98,35 | 98,2 | 98,2 | 98,63 | 98,27 | 98,49 | 98,42 | 98,35 | 98,35 |
| **[23]** | *Streptomyces mexicanus* | 97,77 | 97,77 | 97,92 | 98,2 | 98,27 | 98,2 | 97,92 | 97,92 | 98,06 | 97,77 | 98,2 | 97,92 | 97,77 | 97,77 |
| **[24]** | *Streptomyces albus subsp albus* | 95,61 | 96,12 | 95,54 | 95,4 | 95,4 | 95,54 | 95,26 | 95,26 | 95,69 | 95,54 | 95,69 | 95,69 | 96,05 | 96,05 |
| **[25]** | *Streptomyces somaliensis* | 98,13 | 97,41 | 97,41 | 98,06 | 97,99 | 97,99 | 97,92 | 97,92 | 98,2 | 98,06 | 98,2 | 97,92 | 97,77 | 97,77 |

Table S7- Similarity percentage matrix for the gene 16S rRNA, of LGMB483 - *Streptomyces* genus

|  |  | **[15]** | **[16]** | **[17]** | **[18]** | **[19]** | **[20]** | **[21]** | **[22]** | **[23]** | **[24]** | **[25]** |
| --- | --- | --- | --- | --- | --- | --- | --- | --- | --- | --- | --- | --- |
| **[15]** | *Streptomyces erythrogriseus* |  |  |  |  |  |  |  |  |  |  |  |
| **[16]** | *Streptomyces griseoincarnatus* | 100 |  |  |  |  |  |  |  |  |  |  |
| **[17]** | *Streptomyces griseosporeus* | 97,99 | 97,99 |  |  |  |  |  |  |  |  |  |
| **[18]** | *Streptomyces chromofuscus* | 98,13 | 98,13 | 98,2 |  |  |  |  |  |  |  |  |
| **[19]** | *Streptomyces glomeratus* | 98,2 | 98,2 | 98,42 | 98,71 |  |  |  |  |  |  |  |
| **[20]** | *Streptomyces chiangmaiensis* | 97,99 | 97,99 | 98,06 | 98,99 | 98,63 |  |  |  |  |  |  |
| **[21]** | *Streptomyces lannensis* | 97,7 | 97,7 | 97,7 | 98,35 | 98,42 | 98,63 |  |  |  |  |  |
| **[22]** | *Streptomyces leeuwenhoekii* | 98,27 | 98,27 | 98,35 | 98,35 | 98,49 | 98,56 | 98,35 |  |  |  |  |
| **[23]** | *Streptomyces mexicanus* | 97,7 | 97,7 | 97,92 | 98,99 | 98,56 | 98,35 | 97,77 | 98,42 |  |  |  |
| **[24]** | *Streptomyces albus subsp albus* | 96,05 | 96,05 | 95,9 | 95,97 | 95,76 | 95,54 | 95,11 | 95,97 | 96,62 |  |  |
| **[25]** | *Streptomyces somaliensis* | 97,7 | 97,7 | 98,56 | 98,2 | 98,27 | 98,13 | 97,77 | 98,56 | 98,13 | 95,9 |  |

Table S8- Similarity percentage matrix for the gene 16S rRNA, of LGMB479–*Williamsia* genus

|  |  | **[1]** | **[2]** | **[3]** | **[4]** | **[5]** | **[6]** | **[7]** | **[8]** | **[9]** | **[10]** | **[11]** |
| --- | --- | --- | --- | --- | --- | --- | --- | --- | --- | --- | --- | --- |
| **[1]** | *Williamsia deligens* | 0 | 95,25 | 95,17 | 95,77 | 98,14 | 95,99 | 97,47 | 97,55 | 95,99 | 97,4 | 91,16 |
| **[2]** | *Williamsia faeni* | 95,25 | 0 | 98,22 | 97,99 | 96,06 | 98,22 | 95,77 | 94,28 | 94,06 | 94,28 | 91,9 |
| **[3]** | *Williamsia limnetica* | 95,17 | 98,22 | 0 | 98,74 | 96,29 | 98,96 | 96,58 | 94,87 | 94,95 | 94,87 | 92,12 |
| **[4]** | *Williamsia marianensis* | 95,77 | 97,99 | 98,74 | 0 | 96,88 | 99,78 | 96,58 | 95,1 | 95,02 | 95,02 | 91,6 |
| **[5]** | *Williamsia maris* | 98,14 | 96,06 | 96,29 | 96,88 | 0 | 97,1 | 99,33 | 96,66 | 96,51 | 96,51 | 91,83 |
| **[6]** | *Williamsia muralis* | 95,99 | 98,22 | 98,96 | 99,78 | 97,1 | 0 | 96,81 | 95,32 | 95,17 | 95,25 | 91,83 |
| **[7]** | *Williamsia phyllosphaerae* | 97,47 | 95,77 | 96,58 | 96,58 | 99,33 | 96,81 | 0 | 96,58 | 96,66 | 96,43 | 92,35 |
| **[8]** | *Williamsia serinedens* | 97,55 | 94,28 | 94,87 | 95,1 | 96,66 | 95,32 | 96,58 | 0 | 95,77 | 99,85 | 92,05 |
| **[9]** | *Williamsia sterculiae* | 95,99 | 94,06 | 94,95 | 95,02 | 96,51 | 95,17 | 96,66 | 95,77 | 0 | 95,77 | 92,05 |
| **[10]** | *LGMB479* | 97,4 | 94,28 | 94,87 | 95,02 | 96,51 | 95,25 | 96,43 | 99,85 | 95,77 | 0 | 92,05 |
| **[11]** | *Mycobacterium tuberculosis* | 91,16 | 91,9 | 92,12 | 91,6 | 91,83 | 91,83 | 92,35 | 92,05 | 92,05 | 92,05 | 0 |

Table S9. Dry weight obtained from extractsof endophytic actinomycetes in two media (SG and R5A) and two temperatures (28 °C and 36 °C).

|  | **SG** | | **R5A** | |
| --- | --- | --- | --- | --- |
|  | **28 °C** | **36 °C** | **28 °C** | **36 °C** |
| **LGMB461**  ***Microbispora* sp.** | 326.00 | 9.00 | 43.00 | 11.00 |
| **LGMB465**  ***Microbispora* sp.** | 17.00 | 48.00 | 19.00 | 13.00 |
| **LGMB466 *Actinomadura* sp.** | 12.00 | 13.00 | 17.00 | 65.00 |
| **LGMB471 *Microbacterium* sp.** | 11.00 | 25.00 | 12.00 | 17.00 |
| **LGMB479**  ***Williamsia* sp.** | 139.00 | 11.00 | 9.00 | 8.00 |
| **LGMB482 *Sphaerisporangium* sp.** | 12.00 | 14.00 | 16.00 | 20.00 |
| **LGMB483**  ***Streptomyces* sp.** | 130.00 | 4.00 | 92.00 | 199.00 |
| **LGMB485**  ***Micrococcus* sp.** | 8.00 | 16.00 | 146.00 | 41.00 |
| **LGMB487 *Actinomadura* sp.** | 29.00 | 151.00 | 21.00 | 86.00 |
| **LGMB491 *Aeromicrobium* sp.** | 10.00 | 63.00 | 71.00 | 174.00 |

Values in mg
